# Disulfidptosis-related long non-coding RNA signature predicts the prognosis, tumor microenvironment, immunotherapy, and antitumor drug options in colon adenocarcinoma

**DOI:** 10.1007/s10495-024-02011-x

**Published:** 2024-08-08

**Authors:** Kang Wang, Jing Yu, Qihuan Xu, Yuanhong Peng, Haibin Li, Yan Lu, Manzhao Ouyang

**Affiliations:** 1 Department of Gastrointestinal Surgery, Shunde Hospital, Southern Medical University (The First People’s Hospital of Shunde Foshan), Shunde, Foshan, Guangdong Province 528300 China; 2 The Second School of Clinical Medicine, Southern Medical University, Guangzhou, Guangdong Province 510080 China; 3 Guangdong Medical University, Zhanjiang, Dongguan 523808 China; 4 GCP Center, Shunde Hospital, Southern Medical University, The First People’s Hospital of Shunde Foshan), Foshan, Guangdong 528300 China

**Keywords:** Disulfidptosis, Colon adenocarcinoma, LncRNA, Immune microenvironment, Immunotherapy, Prognosis

## Abstract

This study aims to investigate the role and prognostic significance of long non-coding RNAs (lncRNAs) associated with disulfidptosis in colon adenocarcinoma (COAD). The TCGA database’s clinical data and transcriptome profiles were employed. Analysis of previous studies identified 10 disulfidptosis-related genes (DRGs). We used these genes to construct a signature that could independently and accurately predict the prognosis of patients with COAD. The Kaplan-Meier (K-M) curve analysis showed that the lower-risk group had a better prognosis. With the help of multivariate Cox regression analysis, the risk score produced from the patient’s signature might independently predict the outcomes. Utilizing a nomogram, the receiver operating characteristic (ROC) curve, and principal component analysis (PCA), the signature’s predictive ability was also confirmed. It’s interesting to note that immunotherapy, especially PD-1 immune checkpoint suppression, was more likely to benefit low-risk patients. The IC50 levels for certain anticancer agents were lower in the high-risk group. Finally, qRT-PCR analyses in colon cancer cell lines revealed elevated levels of lncRNAs CASC9, ZEB1-AS1, ATP2A1-AS1, SNHG7, AL683813.1, and AP003555.1, and reduced levels of FAM160A1-DT and AC112220.2, compared to normal cell lines. This signature offers insights into prognosis, tumor microenvironment, and options for immunotherapy and antitumor drugs in patients with COAD.

**Supplementary Information:**

The online version contains supplementary material available at 10.1007/s10495-024-02011-x.

## Introduction

According to the 2020 GLOBOCAN data from IARC, colon adenocarcinoma (COAD) had an estimated global incidence of 1,148,515 cases, which places it as the fifth most common kind of cancer worldwide. COAD was also the fifth leading cause of mortality, resulting in 576,858 deaths in 2020 [1]. This disease primarily presents as a specific subtype of colon cancer. In addition to surgery, radiation, chemotherapy, targeted therapy, immunotherapy, and other modalities are available as COAD treatment options [2]. The effectiveness of the treatment for COAD determined by factors like disease stage, patient health, and tumor type [3]. Despite advancements in diagnosis and treatment, patients with COAD often experience recurrences and metastases, with a significantly reduced 5-year survival rate [4]. Molecular pathways underpinning COAD development must be studied, and biomarkers must be identified, to improve diagnosis, treatment, and prognosis of the disease.

Recent research has focused a lot of emphasis on a kind of cell death called disulfidptosis [5]. Elevated expression of SLC7A11, coupled with glucose deprivation, leads to intracellular disulfide accumulation that triggers disulfidptosis. Disulfidptosis, in contrast to ferroptosis and apoptosis, influences the cytoskeleton’s actin response to disulfide stress. Previous research emphasizes how blocking glucose transporters may affect the induction of cellular death and control of tumor development, highlighting the relevance of disulfidptosis in cancer therapy. Long non-coding RNAs (lncRNAs), which are essential for the initiation and development of cancer, can be blamed for both tumor promotion and tumor suppression due to their dysregulated production. LncRNAs are novel biomarkers and show great potential as therapeutic targets in cancer [6–8]. For example, the genes LNC01567, BANCR, SLCO4A1-AS1, and Linc00152 are implicated in the development, spread, and metastasis of colon cancer and are linked to chemotherapeutic treatment resistance [9–12]. Further research into the involvement of lncRNAs in disulfidptosis and its underlying mechanisms can help uncover new therapeutic targets for this condition.

This study analyzed publicly available databases to examine the expression and mutations of disulfidptosis-related lncRNAs (DRLs) in COAD. Through classification and prognostic analysis of DRLs, we developed a prognostic risk model incorporating eight lncRNAs. To look into potential reasons for prognosis variations, we carried out an immune infiltration study, immunological checkpoint analysis, and drug susceptibility analysis. Furthermore, the findings were validated by examining the levels of these lncRNAs in clinical samples obtained from COAD patients at Shunde Hospital of Southern Medical University. These findings highlight the function of disulfidptosis in COAD and provide crucial information for creating individualized, precise treatments.

## Materials and methods

### Data download and screening of DRLs

High-throughput RNA sequencing data and clinicopathological information for 476 COAD tissues and 41 normal colon tissues were downloaded from the Cancer Genome Atlas (TCGA) database (https://portal.gdc.cancer.gov/). Data for both mRNA and lncRNA gene expression were normalized. Clinical information included age, gender, staging (T, N, and M), AJCC stage, survival status, and survival time. Ten disulfidptosis-related genes (DRGs) were obtained from the published studies [5]. We employed the Pearson correlation coefficient to identify lncRNAs linked to the expression of DRGs. A criterion of *r* > 0.4 for the correlation coefficient was used as a screening criterion and a significance level of *P* < 0.001. Normalized data was analyzed using the “limma” package and visualized using the “ggalluvial” package.

### Establishment of the DRLs prognostic model

Participants were randomly assigned to training and testing groups using the COAD data. Using a screening criterion of *P* < 0.05 for prognostic significance, univariate Cox regression analysis was used to identify 27 potential candidate DRLs within the training group. In order to avoid overfitting, the least absolute shrinkage and selection operator (LASSO) was used to choose the most pertinent predictors. Eight DRLs were found using multivariate Cox regression analysis. Using these eight DRLs, for predicting the onset of COAD in patients, we developed a risk signature model. Using the following formula, the risk score was determined:

$$\:\text{R}\text{i}\text{s}\text{k}\:\:\text{S}\text{c}\text{o}\text{r}\text{e}\:=\sum\:_{i=1}^{N}\left(Expi\text{*}\text{C}\text{o}\text{e}\text{f}\:\right)$$

N stands for the number of lncRNAs associated with prognosis in this risk signature, Expi for each lncRNA’s expression, and Coef for the regression coefficient calculated using multivariate Cox regression analysis. Data for eight DRLs were used for the correlation study, and a correlation heatmap was created to show the results. Both the training and testing groups used the prognostic model to compute the risk score. Based on the median value of the risk score, the samples were divided into two groups: high-risk and low-risk. The risk curves were plotted and visualized with the R “pheatmap” tool. The “survminer” and “timeROC” packages were used to perform receiver operating characteristic (ROC) analysis and Kaplan-Meier (K-M) survival analysis on the samples.

### Construction and validation of a predictive nomogram

Predictive nomograms were developed by employing the “survival” and “RMS” packages. These nomograms include risk ratings and clinicopathological traits to estimate the likelihood that patients with COAD will survive at 1, 3, and 5 years. To assess the relationship between the expected and actual survival rates, calibration curves were employed.

### Principal component analysis (PCA)

PCA is a common method for data analysis. It transforms the raw data into a set of linearly independent representations of each dimension through linear transformation, which can be used to extract the main feature components of the data and is often used for dimensionality reduction of high-dimensional data [13]. The R “scatterplot3” tool looked at how high-risk and low-risk categories differed from one another.

### Functional enrichment analysis

We employed the “edgeR” package to assess the differentially expressed genes (DEGs) between the high-risk and low-risk groups, utilizing the threshold of (| log2 (fold change) |> 1 and FDR < 0.05) for selection [14]. For the aim of conducting functional enrichment analysis, the “clusterProfiler” package was used to analyze data from the Gene Ontology (GO) database and the Kyoto Encyclopedia of Genes and Genomes (KEGG) database. The GO database details how the cellular component (CC), biological process (BP), and molecular function (MF) work biologically [15]. The KEGG database (https://www.kegg.jp/) was employed to analyze biological pathway information, as it integrates data from genomic, chemical, and functional systems [16]. The “limma” package was used to determine the gene set enrichment analysis (GSEA) score.

### Tumor mutation burden (TMB)

The tumor genome’s TMB value is the number of mutations per million bases [17]. TMB analysis offers important insights into the mechanisms behind the growth and progression of tumors. Comparison of mutation frequencies across patients helps in identifying potential novel therapeutic targets. The TCGA database was used to gather COAD tumor mutation data, and the “maftools” R tool was used for TMB analysis and display of the top 15 genes with the most mutations.

### Tumor immunotherapy analysis

The “estimate” package was utilized to gauge the infiltration of immune cells in the tumor tissue. We used the single-sample GSEA (ssGSEA) algorithm with the package “gsva” to calculate infiltration scores for 29 immune-related pathways and 23 immune cell types. Based on these scores, a heatmap was generated to visualize variations in immune function. The tumour immune dysfunction and exclusion (TIDE) score was collected from the TIDE network (http://tide.dfci.harvard.edu). It offers a prediction meter for cancer patients’ prognosis and their reaction to immunotherapy.

#### Drug sensitivity analysis

The “pRRophetic” package was utilized to predict the half maximal inhibitory concentration (IC50) of common antitumor drugs for colon cancer, and evaluate drugs sensitivity in distinct risk groups. Differences between the two groups were analyzed by Wilcoxon Signed Rank Test.

### Cell culture

Human intestinal epithelial cells NCM460, colon cancer cells LOVO, and HT-29 were cultured with RPMI-1640 medium. Human colon cancer cells SW480, SW620, HCT116, RKO, and Caco-2 were cultured with Dulbecco’s modifified Eagle medium medium. Both media were supplemented with 100 U/mL penicillin and streptomycin as well as 10% fetal bovine serum in a humidifified atmosphere of 5% CO_2_ at 37℃.

### Quantitative real-time polymerase chain reaction (qRT-PCR)

From the Gastroenterology Surgery Specimen Bank of Shunde Hospital, Southern Medical University, sixteen pairs of colon cancer samples and nearby tissues were collected. The Mabio Total RNA Extraction Kit (RNT412-02) extracted total RNA from these samples. The RNA was isolated and subsequently subjected to reverse transcription to generate complementary DNA (cDNA) employing the Vazyme reverse transcription kit (R222-01). Using a Bio-Rad CFX96 equipment, the Vazyme ChamQ Universal SYBR qPCR Master Mix kit (Q711-03) was used to quantify the amounts of lncRNAs.

### Statistical analysis

The R program (version 4.2.2; https://www.r-project.org/) was used to perform Cox regression, Pearson correlation coefficient, and K-M survival analyses. Utilizing the software GraphPad Prism (version 9.0.0), the groups were compared, along with an analysis of variance (ANOVA) statistical test. P-values < 0.05 are indicative of statistical significance.

## Results

### Identification of DRLs with a prognostic value for COAD

**Fig. 1:**
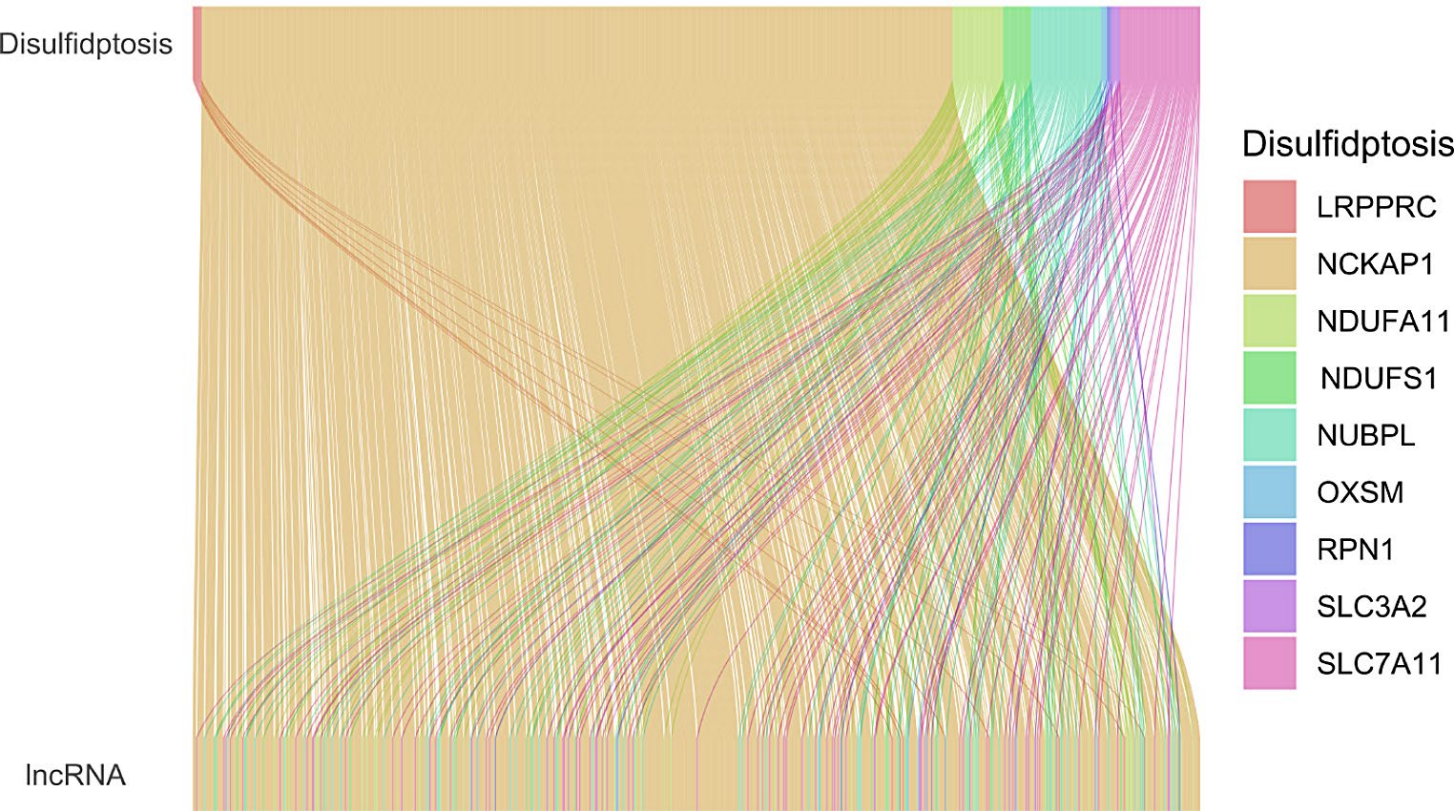
The relationships between DRGs and DRLs in the Sankey diagram

**Fig. 2:**
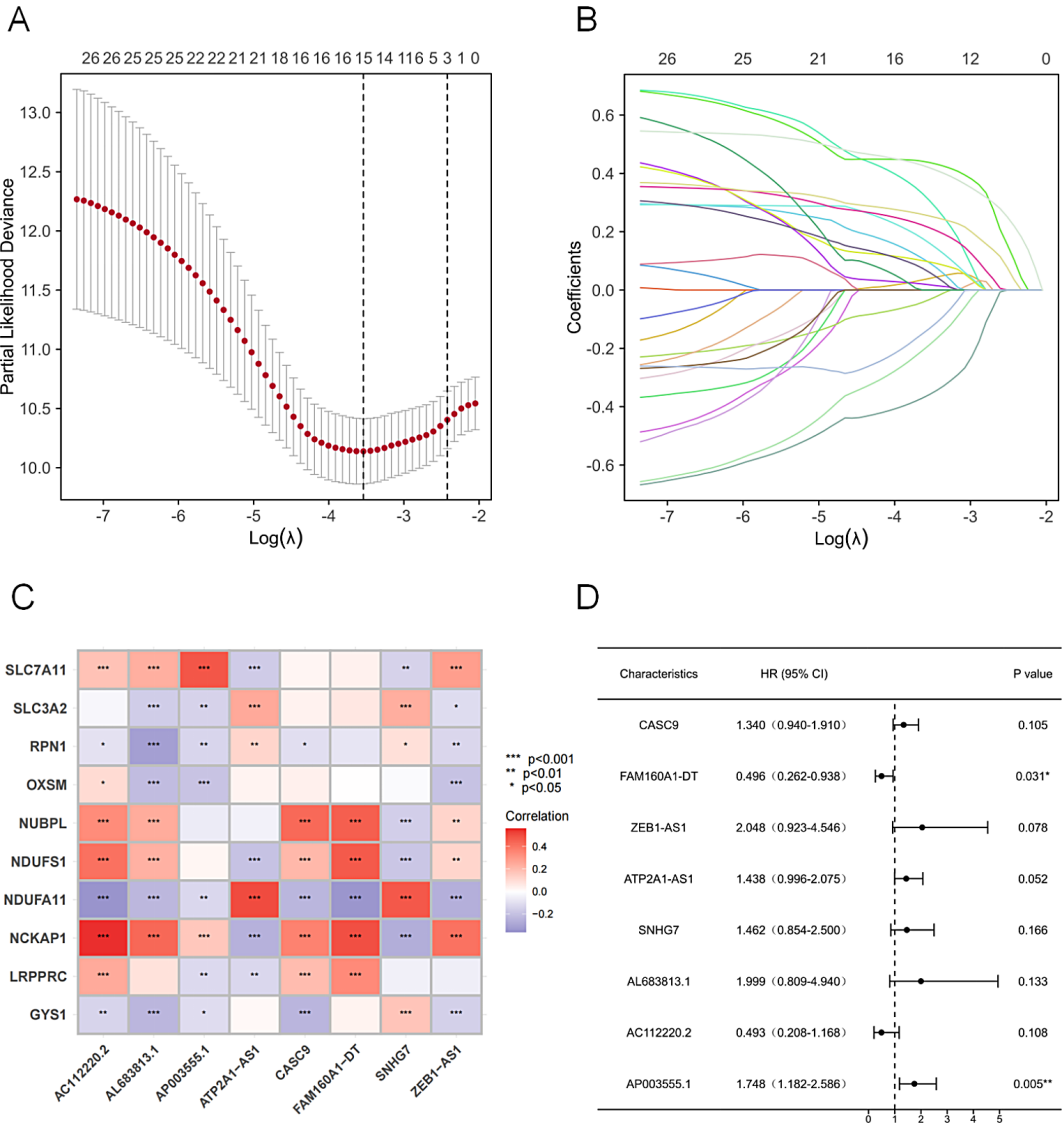
The DRLs with prognostic signifificance were selected. (**A**) The confifidence intervals for each lambda were shown. Dotted vertical lines were drawn at the optimal values by using the minimum criteria. (**B**) Partial likelihood deviance for different numbers of variables. The horizontal axis represents the log value of the independent variable lambda, and the vertical axis represents the coeffificient of the independent variable. (**C**) The correlation between 8 prognostic DRLs and 10 DRGs in the TCGA-COAD cohort. The color of each unit shows the degree of correlation. (**D**) Multivariate Cox regression analysis showed 8 DRLs. P > = 0.05, *P < 0.05, **P < 0.01, ***P < 0.001

The study flow is depicted in Figure S1. Transcript data for 16,876 lncRNAs and 10 DRGs (Table S2) were extracted from the COAD sequences of the TCGA database. Co-expression analysis was done to find lncRNAs that are linked to the DRGs. Based on the criteria of |Pearson R| >0.4 and a p-value < 0.001, we could identify a total of 802 DRLs (Fig. 1) (Table S3). Among these, GYS1 did not have any associated lncRNA. A total of 448 COAD patients were randomly assigned to either the training set (*N* = 224) or the validation set (*N* = 224). The clinical characteristics of the two groups are shown in (Table 1), and the detailed differences between the two groups were not significant. The link between the DRLs and patient survival was investigated by univariate Cox regression analysis of the training set. A total of 27 DRLs exhibited significant prognostic value (Table S4). The LASSO regression analysis then identified and selected 15 lncRNAs linked to disulfidptosis (Fig. 2A, B). Finally, using a multivariate Cox regression analysis to investigate the potential connection between eight DRLs and the clinical outcome of patients diagnosed with COAD. The correlation between these eight DRLs and the DRGs was visualized in a heatmap (Fig. 2C). Among the eight DRLs, namely CASC9, ZEB1-AS1, ATP2A1-AS1, SNHG7, AL683813.1, AP003555.1, FAM160A1-DT, and AC112220.2, six exhibited a hazard ratio (HR) > 1 and were considered poor predictors of prognosis, while the other two, FAM160A1-DT and AC112220.2, were potentially protective indicators (Fig. 2D).

**Table 1:** The clinical characteristics of colon cancer patients in the training and validation group

Characteristics	Training group No.	%	Validation group No.	%	*P*-value
**Age**	-	-	-	-	-
<=65	88	39.29	96	42.86	>0.05
> 65	136	60.71	128	57.14	-
**Gender**	-	-	-	-	-
Female	108	48.21	106	47.32	>0.05
Male	116	51.79	118	52.68	-
**AJCC Stage**	-	-	-	-	-
I	46	20.54	29	12.95	>0.05
II	85	37.95	91	40.62	-
III	55	24.55	69	30.8	-
IV	32	14.29	30	13.39	-
unknown	6	2.68	5	2.23	-
**T stage**	-	-	-	-	-
T1	6	2.68	5	2.23	>0.05
T2	47	20.98	29	12.95	-
T3	147	65.62	158	70.54	-
T4	24	10.71	32	14.29	-
**N stage**	-	-	-	-	-
N0	141	62.95	125	55.8	>0.05
N1	43	19.2	59	26.34	-
N2	40	17.86	40	17.86	-
**M stage**	-	-	-	-	-
M0	162	72.32	168	75	>0.05
M1	32	14.29	30	13.39	-
unknow	30	13.39	26	11.61	-

### Construction and validation of the prognostic model

The prognostic value of the eight DRLs in patients with COAD was investigated using a prognostic model constructed using correlation coefficients from multivariate COX regression. The RiskScore was calculated as follows:

$$\eqalign{{\rm{RiskScore}} & {\rm{ = CASC9 * 0}}{\rm{.35 - FAM160A1 - DT * 0}}{\rm{.43 }} \cr & {\rm{ + ZEB1 - AS1 * 0}}{\rm{.51 + ATP2A1 - AS1 * 0}}{\rm{.35 }} \cr & {\rm{ + SNHG7 * 0}}{\rm{.49 + AL683813}}{\rm{.1 * 0}}{\rm{.74 }} \cr & {\rm{ - AC112220}}{\rm{.2 * 0}}{\rm{.58 + AP003555}}{\rm{.1 * 0}}{\rm{.54}}{\rm{.}} \cr}$$

**Fig. 3:**
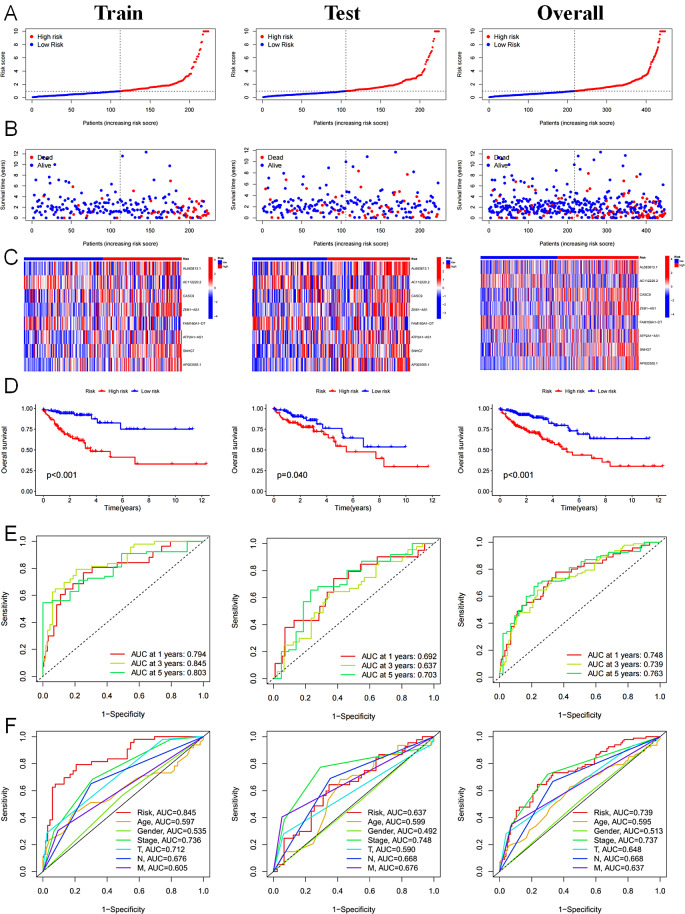
Evaluation of prognosis prediction ability of signature. (**A**) Risk scores in the high-risk and low-risk groups. (**B**) Survival status of patients with COAD in high-risk and low-risk groups. (**C**) Expression of the 8 DRLs. (**D**) K-M survival analysis of the high-risk group and the low-risk group based on the TCGA dataset. (E, F) ROC curve of the signature. *P* < 0.05, statistically signifificant

**Fig. 4:**
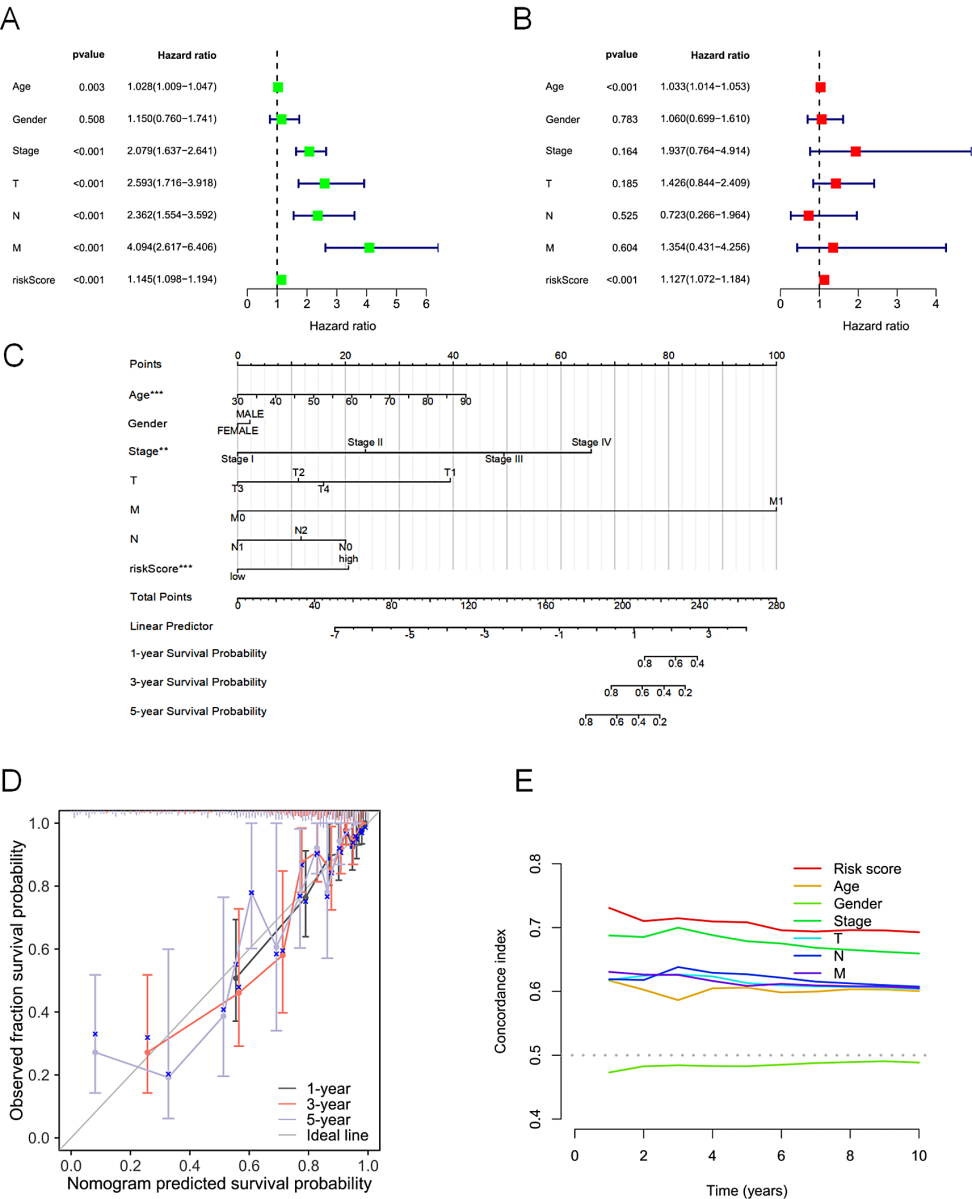
The verifification of the independent prognostic ability and clinical predictive ability of the signature. Forest plots of univariate (**A**) and multivariate (**B**) Cox regression analyses revealed that risk score could be an independent prognostic factor. (**C**) The nomogram combining risk scores and clinical variables could predict the probability of survival based on the overall score. (**D**) The calibration curves for predicting patients’ OS at 1-year, 3-year, and 5-year. The diagonal line (gray) represents the ideal case. (**E**) The C-index curves for assessing the discrimination ability of risk score and other clinical factors at each time point. P > = 0.05, *P < 0.05, **P < 0.01, ***P < 0.001

Risk scores were computed for both the training and validation sets for each individual patient. Subsequently, the patients were categorized into high-risk and low-risk groups based on the median risk score, which served as the threshold (Fig. 3A). The group classified as high-risk had a greater mortality rate compared to the group classified as low-risk (Fig. 3B). Additionally, significant variations in the expression levels of DRLs within the signature were seen between the two groups (Fig. 3C). The low-risk group demonstrated elevated levels of two lncRNAs, FAM160A1-DT and AC112220.2, that acted as protective factors. In contrast, six lncRNAs that were risk factors were present at elevated levels in the high-risk group (Fig. 3C). The K-M survival curve showed a worse outcome for the high-risk group (Fig. 4D). In addition, the ROC curve’s area under the curve (AUC) measurements successfully illustrated the signature’s accuracy. In the provided training set, the AUC was calculated for three different time periods: 1-year, 3-year, and 5-year. The AUC values obtained for these time points were 0.794, 0.845, and 0.803, respectively. Similarly, in the validation set, the respective AUC values at 1, 3, and 5 years were 0.692, 0.637, and 0.703 (Fig. 4E). The AUC for the risk score in the complete dataset was 0.739, which was greater than the AUC values for age, sex, and stage (Fig. 4F). The results mentioned above suggest that the risk score obtained from the prognostic model can serve as a reliable prognostic indicator. In addition, it was observed that individuals classified in the high-risk group had an adverse prognosis in the COAD.

### The risk score could function as an independent prognostic factor

In this study, both univariate and multivariate Cox regression analyses were used to evaluate the predictive value of risk scores of DRLs in patients with COAD. The findings revealed a significant association between overall survival (OS) in patients with COAD and the risk score of DRLs, suggesting the possibility for the risk score is intended to function as an independent prognostic factor (Fig. 4A, B). A nomogram was developed by incorporating clinicopathological characteristics and risk scores to aid in the clinical prediction of patient survival rates (Fig. 4C). The predictive ability of the signature was demonstrated to be highly accurate in predicting patient outcomes, as seen by the well-calibrated curves for overall survival at 1, 3, and 5 years (Fig. 4D). Additionally, the signature demonstrated a higher C-index than any other risk factor, providing further evidence of its superior predictive performance (Fig. 4E). Therefore, combining risk scores and clinical variables can significantly assist in predicting outcomes for patients with COAD.

### Clinical characteristic subgroups and PCA

**Fig. 5:**
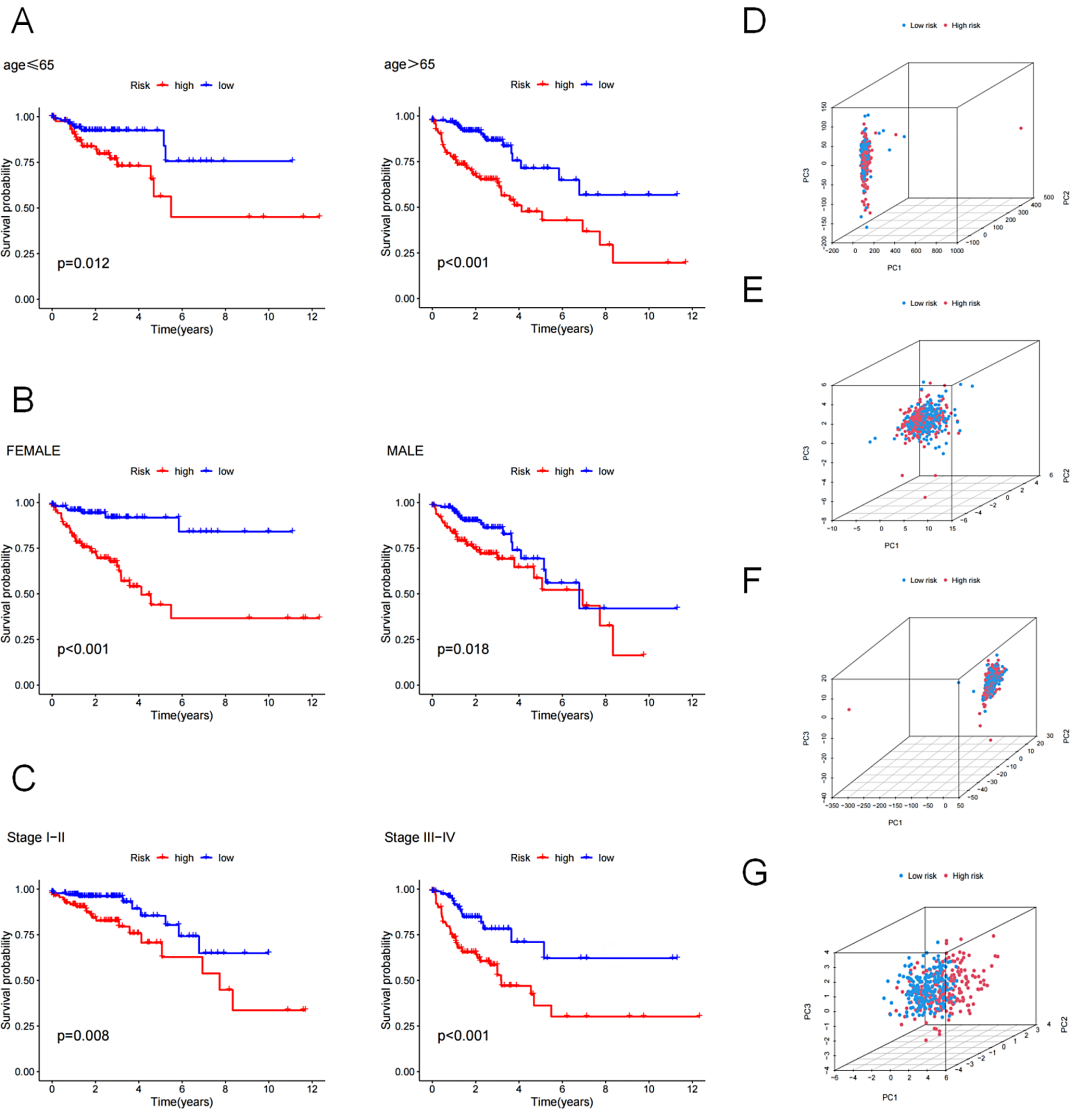
The subgroups survival analysis stratified by age (**A**), gender (**B**), and stage (**C**). The distribution of samples based on the expression of all genes (**D**), the 10 DRGs (**E**), all lncRNAs (**F**), and the eight DRLs (**G**). *P* < 0.05, statistically signifificant

To investigate any possible influence of clinical features on the predictive power of prognostic signature, we grouped patients based on different clinical features and performed survival analyses. The prognostic signatures were not affected by age, gender or stage (Fig. 5A, B, C). PCA was further performed in all patients based on the expression levels of all genes, the ten DRGs, all lncRNAs or the eight DRLs. We failed to obtain clearly differentiated sample grouping from the previous three features (Fig. 5D, E, F). However, based on the expression of eight DRLs on the prognostic signature, patients with COAD could be categorized into two groups (Fig. 5G). As a result, patients with varying clinical characteristics can be sorted into high-risk and low-risk categories using this prognostic signature.

### Exploring differences of biological functions and pathways

**Fig. 6:**
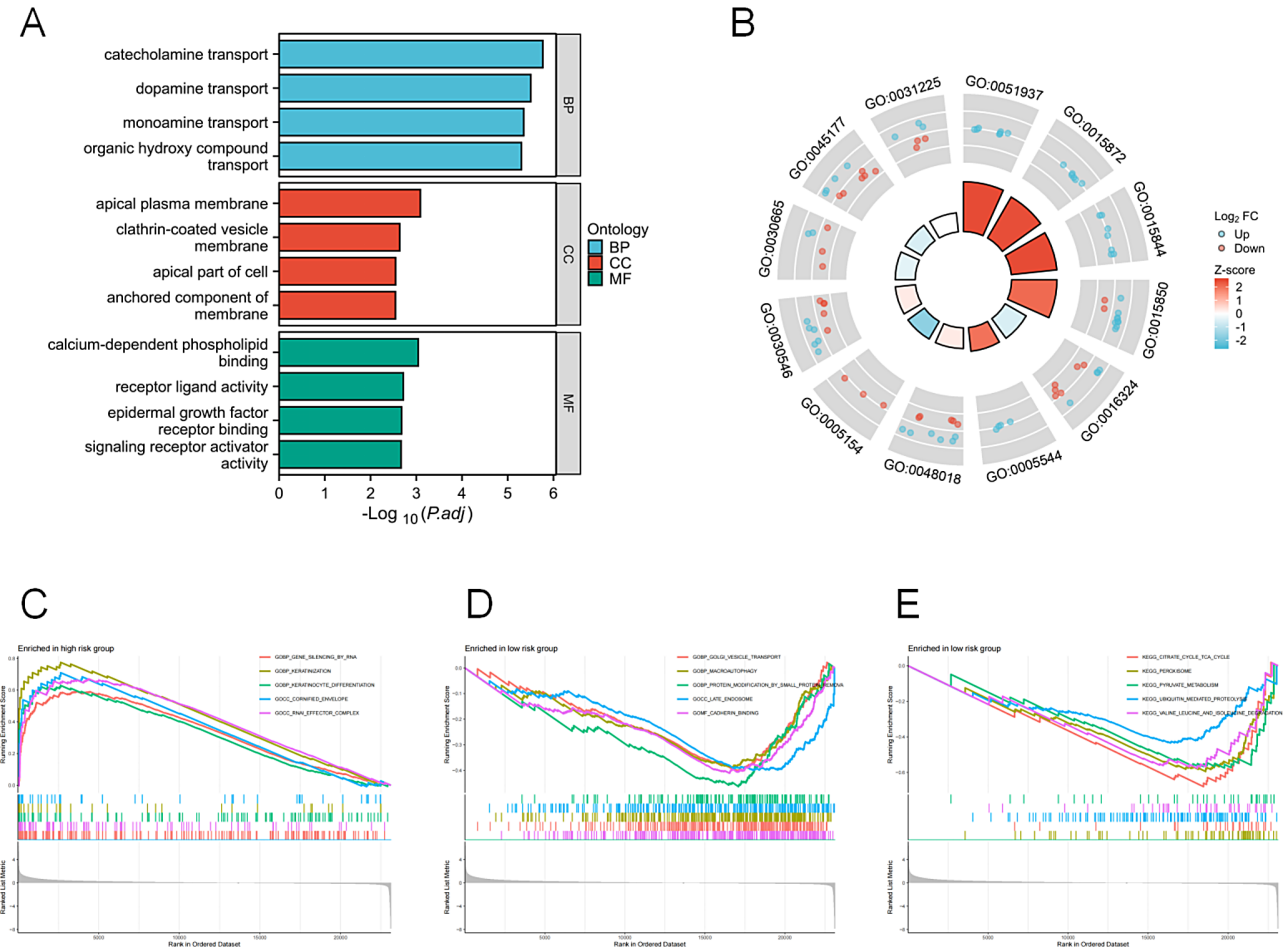
The differences of biological functions and pathways in high-risk and low-risk groups. (**A**) GO and KEGG enrichment analysis of DEGs. (**B**) The expression of DEGs. The GSEA score of high-risk group (**C**) and low-risk group (**D**,**E**)

The study involved the analysis of gene expression difference in both the high-risk and low-risk groups and discovered 257 DEGs (Table S5) to evaluate differences in biological function. The GO functional enrichment and KEGG analysis of DEGs showed the enrichment of these genes in the BP involved in catecholamine transport, dopamine transport, monoamine transport, and organic hydroxy compound transport (Fig. 6A), with no obvious pathway enrichment. The circle diagram further shows the regulation of DEGs (Fig. 6B). A GSEA was conducted to identify various cellular fractions, biological activities, and molecular pathways in both the high-risk and low-risk groups. In the high-risk group, the GENE SILENCING BY RNA, KERATINIZATION and KERATINOCYTE DIFFERENTIATION were upregulated (Fig. 6C), while in the low-risk group, GOLGI VESICLE TRANSPORT, MACROAUTOPHAGY and PROTEIN MODIFICATION BY SMALL PROTEIN REMOVA were upregulated (Fig. 6D). Meanwhile, the low-risk group also identified increases in the pathways for CITRATE CYCLE TCA CYCLE, PEROXISOME, PYRUVATE METABOLISM, UBIQUITIN MEDIATED PROTEOLYSIS and VALINE LEUCINE AND ISOLEUCINE DEGRADATION (Fig. 6E).

### Tumor mutation burden

**Fig. 7:**
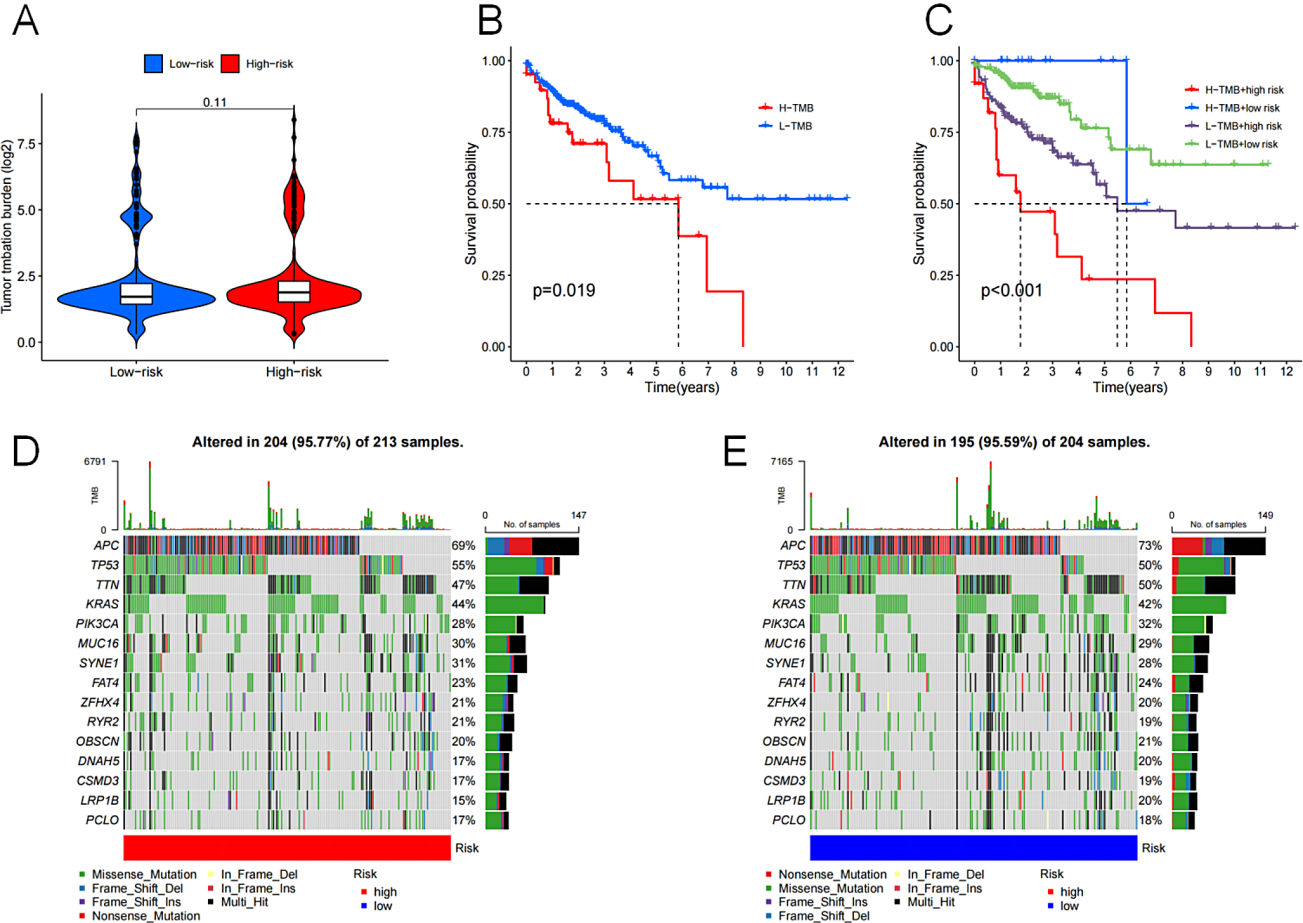
The TMB and genetic mutation analysis of COAD patients. (**A**) The TMB scores of high-risk and low-risk groups. (**B**) K–M survival analysis based on the TMB score. (**C**) K–M survival analysis based on the risk score and the TMB score. The waterfall graphs of colon cancer mutated genes in high-risk group (**D**) and in low-risk group (**E**). *P* < 0.05, statistically signifificant

The TMB of the COAD patient cohorts with high-risk and low-risk did not significantly differ (Fig. 7A). Nevertheless, we identified an association between higher TMB and a notably poorer prognosis among these patients. Importantly, a more accurate prognosis prediction was achieved by combining the TMB score and risk score (Fig. 7B). Patients with high TMB and high-risk ratings had the worst prognosis, while individuals with low TMB and low-risk ratings demonstrated a somewhat improved prognosis with time (Fig. 7C). Genetic mutation analysis revealed variations in the frequency of mutations in colon cancer genes in the two groups [18], including *APC* (69 vs. 73%), *TP53* (55 vs. 50%), *KRAS* (44 vs. 42%), and *PIK3CA* (28 vs. 32%) (Fig. 7D). These findings highlight the significance of disulfidptosis in COAD development and progression.

### Analysis of COAD immunotherapy

**Fig. 8:**
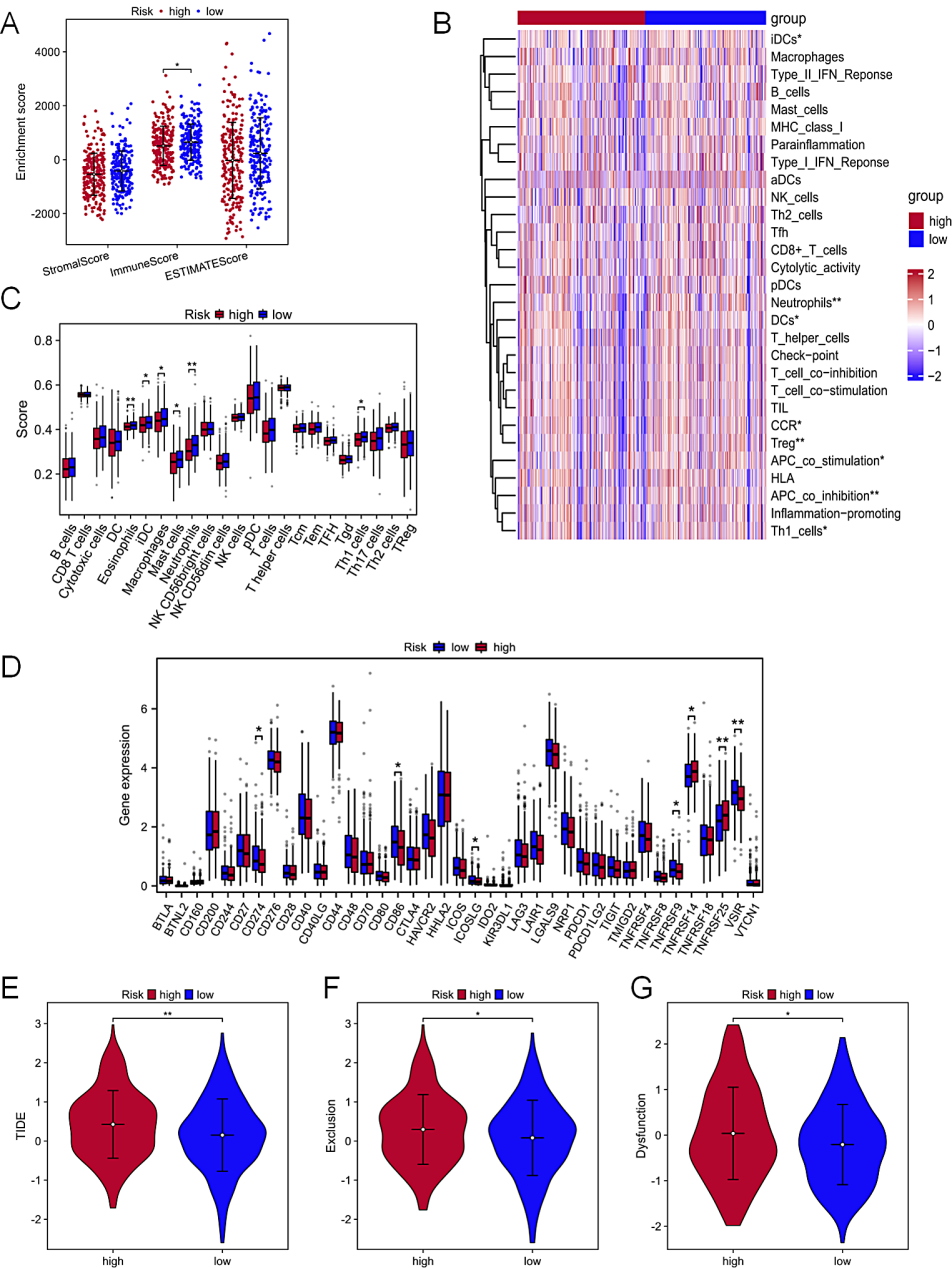
The immunotherapy analysis of patients with COAD. (**A**) ESTIMAT analysis about tumor microenvironment. The ssGSEA scores of 29 immune-related functions (**B**) and 23 immune cells (**C**). Immune checkpoints differences between two risk groups (**D**). TIDE score (**E**), T cells exclusion score (**F**), T cells dysfunction score (**G**). P > = 0.05, *P < 0.05, **P < 0.01, ***P < 0.001

The use of immune checkpoint inhibitors has altered cancer treatment, and the tumor immune microenvironment is essential to the development and growth of cancers. Using expression data, our ESTIMATE analysis, which calculates the number of stromal and immune cells in malignant tumor tissues showed that the low-risk group had higher infiltration of immune cell levels (Fig. 8A). Further exploration of immune-related functions could significantly differentiate immune status between the high-risk and low-risk groups. Specifically, the low-risk group showed higher levels of APC-co-inhibition, Treg, Neutrophils, CCR, Th1-cells, APC-co-stimulation, DCs, and iDCs (Fig. 8B). Conversely, the high-risk group exhibited a reduced abundance of immune cells like Eosinophils, iDC, and macrophages (Fig. 8C). Immune checkpoint gene expression analysis revealed that, except for TNFRSF14 and TNFRSF25, most differentially expressed genes were highly expressed in the low-risk group (Fig. 8D). The high-risk group also had higher TIDE scores (Fig. 8E), indicating a greater probability of tumor immunological exclusion (Fig. 8F) and immune dysfunction (Fig. 8G). Remarkably, the tendency of patients in the low-risk group to evade the immune system was lower, and such patients also displayed superior responses to immunotherapy. These findings strongly highlight the close association between disulfidptosis and tumor immunotherapy.

### The significance of the model in antitumor drug options

**Fig. 9:**
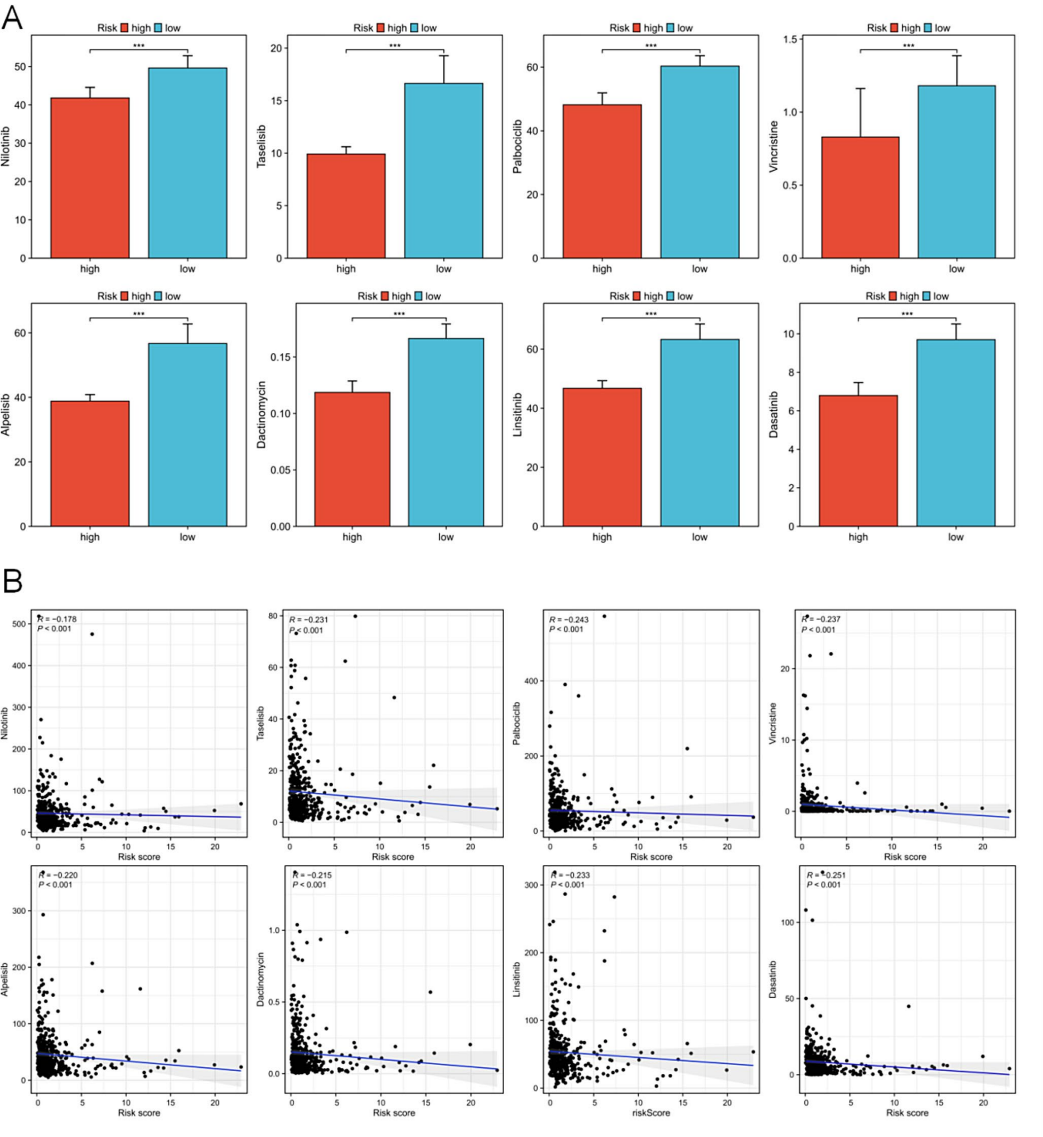
The analysis of drug sensitivity in patients with COAD. Drug sensitivity (**A**) and correlation (**B**) analysis of nilotinib, taselisib, palbociclib, vincristine, alpelisib, dactinomycin, linsitinib and dasatinib. P > = 0.05, *P < 0.05, **P < 0.01, ***P < 0.001

We evaluated the effectiveness of many anticancer medications, including nilotinib, taselisib, palbociclib, vincristine, alpelisib, dactinomycin, linsitinib, and dasatinib, on COAD patients with varying risk scores. Distinguishable variations in drug sensitivity were seen between the high-risk and low-risk groups (Fig. 9A). Particularly, patients in the high-risk group showed greater sensitivity to all eight anticancer medicines tested than those in the low-risk group (Fig. 9B). These findings imply that the current risk model could provide valuable insights for guiding clinical treatment strategies and resistance prevention in patients with COAD.

#### Validation of expression of the eight DRLs

**Fig. 10:**
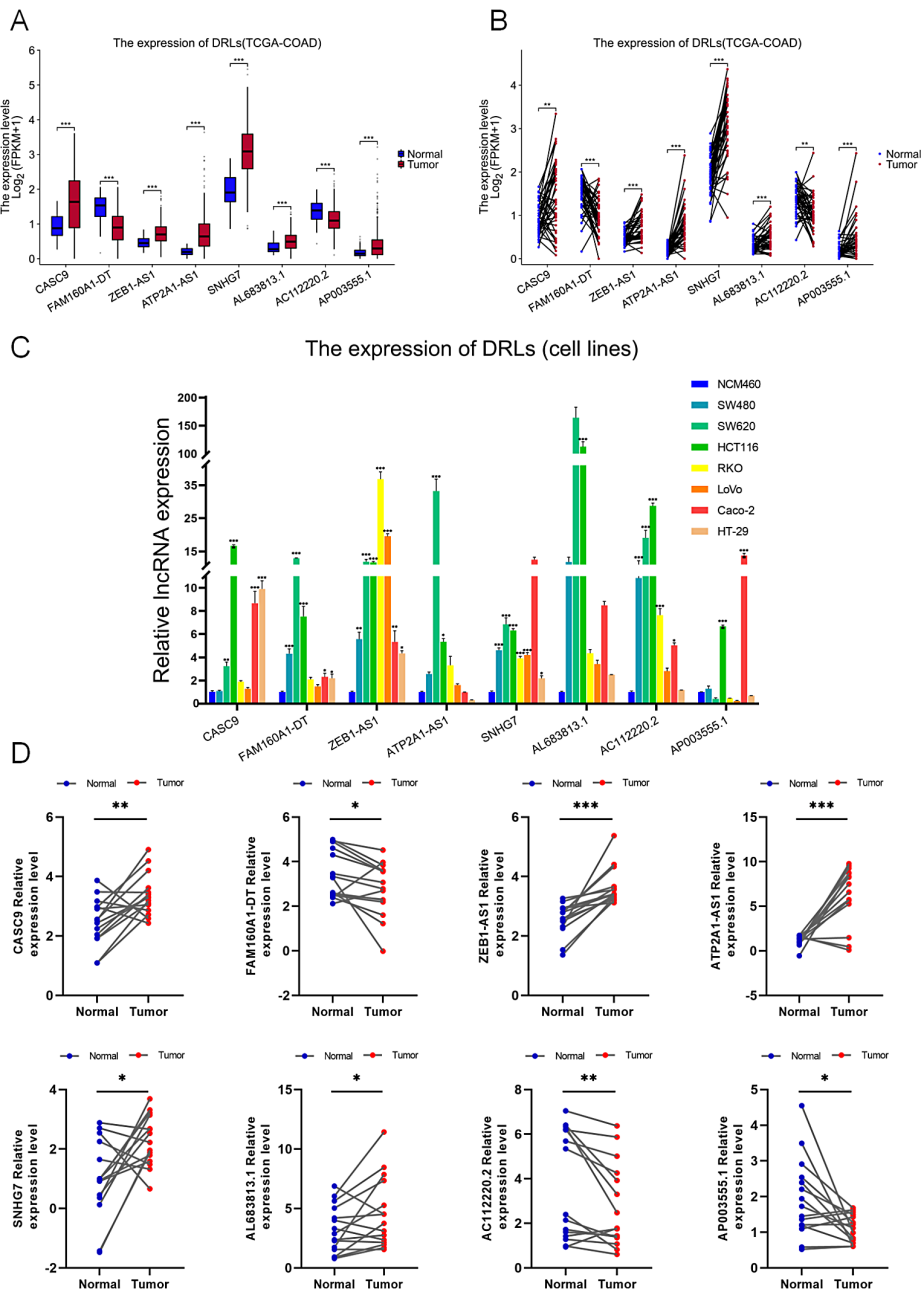
The expression analysis of eight DRLs in cell lines and human tissues. Expression levels of CASC9, FAM160A1-DT, ZEB1-AS1, ATP2A1-AS1, SNHG7, AL683813.1, AC112220.2 and AP003555.1 in the TCGA-COAD data (**A**, **B**), eight cell lines (**C**), and 16 pairs of colon cancer tissue samples (**D**). P > = 0.05, *P < 0.05, **P < 0.01, ***P < 0.001

Using the data from TCGA-COAD database, we first analyzed the expression levels of eight DRLs in patients with COAD (Fig. 10A), comprising 41 paired samples (Fig. 10B). The analysis showed high expression of CASC9, ZEB 1-AS1, ATP2A1-AS1, SNHG7, AL683813.1 and AP003555.1 in colon cancer tissues, while FAM160A1-DT, AC112220.2 showed higher expression in normal tissues than in cancer tissues. The expression of eight DRLs was further verified in colon cancer cell lines (SW480, SW620, HCT116, RKO, Caco-2, LOVO, and HT-29) (Fig. 10C). The results showed that all eight DRLs were upregulated in colon cancer cell lines compared to human normal enterocyte NCM460 cell line, especially in HCT116 cell line. Finally, further assessment of our findings using 16 paired clinical patient samples revealed consistent results with the TCGA dataset (Fig. 10D). These assessments collectively demonstrate the significant variations in the expression of the eight DRLs in COAD, suggesting their potential involvement in regulating the onset and progression of the disease. However, the results obtained from our previous analysis of the tumor-immune microenvironment in the high-risk and low-risk cohorts revealed that patients belonging to the low-risk group exhibited a greater degree of immunological connection, and the differential expression of two protective factors FAM160A1-DT and AC112220.2 tumour immunological microenvironment may impact cell lines and tissues.

## Discussion

The process of cellular death plays a crucial role in the biological progression and the preservation of internal environmental stability. Targeting cell death-related pathways has become a significant focus in cancer therapy to effectively eliminate cancer cells. Disulfidptosis is a recently discovered entirely new mode of cell death. It differs from recognized modes, including apoptosis, necroptosis, pyroptosis, autophagy, ferroptosis, and cuproptosis [19]. SLC7A11 high expression, glucose starvation, and aberrant disulfide bonds are the three signature characteristics of disulfidptosis. Firstly, glucose deprivation leads to nicotinamide adenine dinucleotide phosphate hydrogen (NADPH) depletion [20]. Then, high uptake of extracellular cysteine and excessive accumulation of intracellular cysteine, exacerbating disulfide bond stress in cell metabolism [21, 22]. Finally, abnormal disulfide bond formation in actin cytoskeletal proteins, further leading to disulfidoptosis [5]. Understanding and characterizing mechanisms of disulfidoptosis will deepen our knowledge of cellular homeostasis and open new possibilities for cancer treatment [23]. Some tumors, including lung adenocarcinoma and cervical cancer, have established disulfideptosis-related signatures [24, 25]. Disulfidoptosis-related prognostic signatures may be potential biomarkers for predicting clinical outcomes and treatment effectiveness in patients with cancer. Limited research on disulfideptosis-related prognosis in colon cancer necessitates the development of a risk signature to guide the prognosis and treatment of COAD.

We developed an eight-DRL risk signature as a biomarker for the diagnosis, prognosis, and choice of immunotherapy and chemotherapy strategies for COAD. The Pearson correlation analysis was conducted on the TCGA-COAD dataset to investigate the association between lncRNAs and 10 DRGs. Several analysis were performed on a pool of 802 lncRNAs molecules using univariate Cox regression, LASSO regression, and multivariate Cox regression and the presence of eight DRLs was identified and confirmed (CASC9, FAM160A1-DT, ZEB1-AS1, ATP2A1-AS1, SNHG7, AL683813.1, AC112220.2, AP003555.1). These DRLs were then used to construct the risk signature model. The prognostic value of the risk signature derived from these DRLs was assessed using K-M, ROC, and calibration curves in patients with COAD. This signature is an independent prognostic factor with significant clinical implications.

Prior studies have revealed a strong correlation of CASC9 with hepatocellular carcinoma, breast cancer, osteosarcoma, and pancreatic cancer progression [26–29] and its role in enhancing tumor resistance to gefitinib and gemcitabine [30–32]. However, Liu and Zhang et al. noted contrasting findings and reported that CASC9 silencing suppressed colon cancer cell proliferation and invasion [33, 34]. The risk signature for cuproptosis in colon cancer patients includes ZEB1-AS1 and SNHG7 [35, 36], while ATP2A1-AS1 was incorporated into the lncRNA-based prognosis prediction model for cuproptosis in patients with cervical cancer [37]. SNHG7 has also been implicated in developing drug resistance in COAD [38], such as promoting anlotinib resistance in colon cancer [39]. Studies on AP003555.1 primarily examined the link between ferroptosis and colon cancer, concentrating on the potential role of SLC7A11-mediated cystine absorption in inhibiting ferroptosis [5]. A literature search of DRLs in the current model revealed close association of several lncRNAs with colon cancer prognosis, cuproptosis, and ferroptosis, suggesting that disulfidptosis, cuproptosis, and ferroptosis may be connected in people with colorectal carcinoma, thus warranting further investigation. Multiple DRLs have also been implicated in antitumor drug resistance. Our risk model predicted that the high-risk group would be more sensitive to several anticancer medications, including Nilotinib, Taselisib and Palbociclib. These findings highlight the potential of DRLs in advancing research on understanding tumor drug resistance and guiding the selection of antitumor medications for patients with colon cancer. Furthermore, the lack of studies on FAM160A1-DT, AL683813.1, and AC112220.2 presents opportunities for future research on these entities in the context of COAD.

The tumor microenvironment depends significantly on the immune environment, crucial in driving disease progression and modulating the response to cancer treatment. Immunotherapy, an emerging treatment modality, is often employed for patients with advanced cancer or metastasis [40]. We compared immune cell infiltration and related pathways involved in immune function between two distinct groups (high-risk and low-risk) of patients. The immune infiltration traits were more strongly expressed in the low-risk patient group. Particularly, elevated expression of CD274 (PD-L1) and CD86 among the 39 immune checkpoint-related genes was found in the low-risk group. In the tumor microenvironment, the immune checkpoint pathway, including programmed cell death 1 (PDCD1, PD-1), is essential for inhibiting T cell-mediated antitumor immune system responses. A variety of malignancies, particularly colorectal cancer with significant microsatellite instability, have shown improved clinical outcomes when treated with inhibitors targeting CD274 (PD-L1) or PDCD1 (PD-1) proteins [41]. Kennedy et al. demonstrated that CD86 is a key target of CTLA-4 immune regulation and showed that a deficiency in CTLA-4-mediated CD86 transendocytosis is associated with autoimmunity [42]. The TIDE score showed that the low-risk group responded to immunotherapy more favorably than the high-risk group. The subclass mapping algorithm further validated these findings. The findings suggested a potential correlation between the expression of and immune cell infiltration. The low-risk group showed increased Th1 cell infiltration. Some studies have reported that the imbalance of Th1 cell expression can lead to abnormal secretion of related cytokines, which can enhance tumor immune evasion function and ultimately promote tumor growth, metastasis and progression [43]. Treg cells, another immune cell known to modulate multiple immune responses, also demonstrated comparable levels of infiltration in low-risk group. In recent searches, the heterogeneity of Treg cells in colon cancer has been highlighted. Elevated Treg cell infiltration has been linked to the suppression of tumor growth and a favorable prognosis [44]. According to the above studies, immune checkpoint therapy may benefit low-risk persons more than high-risk ones. Furthermore, immune cell infiltration and heterogeneity in colon cancer provide therapeutic opportunities. In summary, our proposed risk model can guide patients in making informed decisions regarding immunotherapy.

Analysis of the burden of gene mutations in patients with cancer provides insights into tumor onset, progression, and potential therapeutic targets. Although there was no obvious distinction between the two groups’ total TMB, there were substantial differences in the frequency of mutations in certain genes linked to colorectal cancer, such as *APC*, *TP53*, *KRAS*, and *PIK3CA*. It may be possible to use *PIK3CA* gene mutations in colorectal cancer as molecular biomarkers to predict how well adjuvant aspirin therapy would work [45]. A clinical trial based on 76 colorectal cancer patients also confirmed that Adagrasib (an oral small molecule inhibitor of mutant KRAS G12C protein) had antitumor activity in patients with metastatic colorectal cancer [46]. In evaluating the effect of TMB on OS, our study revealed that low-risk patients with low TMB score had best the prognosis, whereas high-risk patients with high TMB score had the worst prognosis. Thus, the therapeutic and predictive significance of mutations in tumor genes in different patients should not be ignored.

In this study, we created and verified a prognostic signature using DRLs, which showed excellent accuracy in assessing prognosis and treatment outcomes in patients with COAD. Our research has certain limitations too. The signature is constructed using a public database and lacks clinical actual sample data. Additionally, due to the scarcity of data on lncRNAs expression and clinical data in other databases, the validation of the model was limited to a single database. To examine the molecular pathways connecting DRLs to the prognostic signature, more functional experiments are required.

## Conclusion

In conclusion, our study identified eight DRLs and used these genes to construct a signature that can independently and accurately predict the prognosis of COAD patients. By further combining signature with the clinical characteristics of patients, it can help clinicians to achieve clinical stratification management of patients. Moreover, this signature can not only predict immunotherapy in COAD patients, but also guide their selection of antitumor agents.

## Electronic supplementary material

Below is the link to the electronic supplementary material.

Supplementary Material 1

Supplementary Material 2

Supplementary Material 3

Supplementary Material 4

Supplementary Material 5

Supplementary Material 6

## Data Availability

No datasets were generated or analysed during the current study.

## References

[1] Sung H, Ferlay J, Siegel RL et al (2021) Global Cancer statistics 2020: GLOBOCAN estimates of incidence and Mortality Worldwide for 36 cancers in 185 countries. CA Cancer J Clin 71(3):209–249. 10.3322/caac.21660 33538338 10.3322/caac.21660

[2] Dekker E, Tanis PJ, Vleugels JLA, Kasi PM, Wallace MB (2019) Colorectal cancer. Lancet 394(10207):1467–1480. 10.1016/S0140-6736(19)32319-0 31631858 10.1016/S0140-6736(19)32319-0

[3] Stintzing S (2014) Management of colorectal cancer. F1000Prime Rep 6:108. 10.12703/P6-108. Published 2014 Nov 4 25580262 10.12703/P6-108 PMC4229728

[4] Biller LH, Schrag D (2021) Diagnosis and treatment of metastatic colorectal Cancer: a review. JAMA 325(7):669–685. 10.1001/jama.2021.0106 33591350 10.1001/jama.2021.0106

[5] Liu X, Nie L, Zhang Y et al (2023) Actin cytoskeleton vulnerability to disulfide stress mediates disulfidptosis. Nat Cell Biol 25(3):404–414. 10.1038/s41556-023-01091-2 36747082 10.1038/s41556-023-01091-2 PMC10027392

[6] Xu YC, Liang CJ, Zhang DX et al (2017) LncSHRG promotes hepatocellular carcinoma progression by activating HES6. Oncotarget 8(41):70630–70641 Published 2017 Aug 3. 10.18632/oncotarget.19906 29050307 10.18632/oncotarget.19906 PMC5642582

[7] Zhu P, Wang Y, Wu J et al (2016) LncBRM initiates YAP1 signalling activation to drive self-renewal of liver cancer stem cells. Nat Commun. ;7:13608. Published 2016 Dec 1. 10.1038/ncomms13608 10.1038/ncomms13608 PMC5146280 27905400

[8] Xu Y, Leng K, Li Z et al (2017) The prognostic potential and carcinogenesis of long non-coding RNA TUG1 in human cholangiocarcinoma. Oncotarget 8(39):65823–65835 Published 2017 Jul 22. 10.18632/oncotarget.19502 29029475 10.18632/oncotarget.19502 PMC5630375

[9] Yu X, Mi L, Dong J, Zou J (2017) Long intergenic non-protein-coding RNA 1567 (LINC01567) acts as a sponge against microRNA-93 in regulating the proliferation and tumorigenesis of human colon cancer stem cells. BMC Cancer 17(1):716 Published 2017 Nov 6. 10.1186/s12885-017-3731-5 29110645 10.1186/s12885-017-3731-5 PMC5674857

[10] Shen X, Bai Y, Luo B, Zhou X (2017) Upregulation of lncRNA BANCR associated with the lymph node metastasis and poor prognosis in colorectal cancer. Biol Res 50(1):32. 10.1186/s40659-017-0136-5. Published 2017 Oct 2 28969673 10.1186/s40659-017-0136-5 PMC5625712

[11] Yu J, Han Z, Sun Z, Wang Y, Zheng M, Song C (2018) LncRNA SLCO4A1-AS1 facilitates growth and metastasis of colorectal cancer through β-catenin-dependent wnt pathway. J Exp Clin Cancer Res 37(1):222. 10.1186/s13046-018-0896-y. Published 2018 Sep 10 30201010 10.1186/s13046-018-0896-y PMC6131861

[12] Yue B, Liu C, Sun H et al (2018) A positive feed-Forward Loop between LncRNA-CYTOR and Wnt/β-Catenin signaling promotes metastasis of Colon cancer. Mol Ther 26(5):1287–1298. 10.1016/j.ymthe.2018.02.024 29606502 10.1016/j.ymthe.2018.02.024 PMC5993983

[13] Mi JX, Zhang YN, Lai Z, Li W, Zhou L, Zhong F (2019) Principal component analysis based on nuclear norm minimization. Neural Netw 118:1–16. 10.1016/j.neunet.2019.05.020 31228720 10.1016/j.neunet.2019.05.020

[14] Robinson MD, McCarthy DJ, Smyth GK (2010) edgeR: a Bioconductor package for differential expression analysis of digital gene expression data. Bioinformatics 26(1):139–140. 10.1093/bioinformatics/btp616 19910308 10.1093/bioinformatics/btp616 PMC2796818

[15] Ashburner M, Ball CA, Blake JA, Botstein D, Butler H, Cherry JM et al (2000) Gene ontology: tool for the unification of biology. The gene ontology consortium. Nat Genet 25(1):25–29. 10.1038/75556 10802651 10.1038/75556 PMC3037419

[16] Kanehisa M, Goto S (2000) KEGG: kyoto encyclopedia of genes and genomes. Nucleic Acids Res 28(1):27–30. 10.1093/nar/28.1.27 10592173 10.1093/nar/28.1.27 PMC102409

[17] Samstein RM, Lee CH, Shoushtari AN et al (2019) Tumor mutational load predicts survival after immunotherapy across multiple cancer types. Nat Genet 51(2):202–206. 10.1038/s41588-018-0312-8 30643254 10.1038/s41588-018-0312-8 PMC6365097

[18] Cancer Genome Atlas Network (2012) Comprehensive molecular characterization of human colon and rectal cancer. Nature 487(7407):330–337 Published 2012 Jul 18. 10.1038/nature11252 22810696 10.1038/nature11252 PMC3401966

[19] Tsvetkov P, Coy S, Petrova B, Dreishpoon M, Verma A, Abdusamad M, Rossen J, Joesch-Cohen L, Humeidi R, Spangler RD et al (2022) Copper induces cell death by targeting lipoylated TCA cycle proteins. Science 375(6586):1254–1261. 10.1126/science.abf0529 35298263 10.1126/science.abf0529 PMC9273333

[20] Xia N, Guo X, Guo Q, Gupta N, Ji N, Shen B, Xiao L, Feng Y (2022) Metabolic flexibilities and vulnerabilities in the pentose phosphate pathway of the zoonotic pathogen Toxoplasma Gondii. PLoS Pathog 18(9):e1010864. 10.1371/journal.ppat.1010864 36121870 10.1371/journal.ppat.1010864 PMC9521846

[21] Koppula P, Zhuang L, Gan B (2021) Cystine transporter SLC7A11/xCT in cancer: ferroptosis, nutrient dependency, and cancer therapy. Protein Cell 12(8):599–620. 10.1007/s13238-020-00789-5 33000412 10.1007/s13238-020-00789-5 PMC8310547

[22] Machesky LM (2023) Deadly actin collapse by disulfidptosis. Nat Cell Biol 25(3):375–376. 10.1038/s41556-023-01100-4 36918690 10.1038/s41556-023-01100-4

[23] Tang D, Kang R, Berghe TV, Vandenabeele P, Kroemer G (2019) The molecular machinery of regulated cell death. Cell Res 29(5):347–364. 10.1038/s41422-019-0164-5 30948788 10.1038/s41422-019-0164-5 PMC6796845

[24] Yang Z, Cao S, Wang F, Du K, Hu F Characterization and prognosis of Biological Microenvironment in Lung Adenocarcinoma through a disulfidptosis-related lncRNAs signature. Genet Res (Camb). 2023;2023:6670514. Published 2023 Aug 4. 10.1155/2023/6670514 10.1155/2023/6670514 PMC10421709 37575978

[25] Liu L, Liu J, Lyu Q et al (2023) Disulfidptosis-associated LncRNAs index predicts prognosis and chemotherapy drugs sensitivity in cervical cancer. Sci Rep 13(1):12470. 10.1038/s41598-023-39669-3. Published 2023 Aug 1 37528124 10.1038/s41598-023-39669-3 PMC10394072

[26] Yao J, Fu J, Liu Y, Qu W, Wang G, Yan Z (2021) LncRNA CASC9 promotes proliferation, migration and inhibits apoptosis of hepatocellular carcinoma cells by down-regulating miR-424-5p. Ann Hepatol 23:100297. 10.1016/j.aohep.2020.100297 33346094 10.1016/j.aohep.2020.100297

[27] Fan SJ, Cui Y, Li YH et al (2022) LncRNA CASC9 activated by STAT3 promotes the invasion of breast cancer and the formation of lymphatic vessels by enhancing H3K27ac-activated SOX4. Kaohsiung J Med Sci 38(9):848–857. 10.1002/kjm2.12573 35860965 10.1002/kjm2.12573 PMC11896238

[28] Qiu H, Yang D, Li X, Feng F (2022) LncRNA CASC9 promotes cell proliferation and invasion in osteosarcoma through targeting miR-874-3p/SOX12 axis. J Orthop Surg Res. ;17(1):460. Published 2022 Oct 20. 10.1186/s13018-022-03340-w 10.1186/s13018-022-03340-w PMC9585709 36266695

[29] Zhou J, Song G, Su M, Zhang H, Yang T, Song Z (2023) Long noncoding RNA CASC9 promotes pancreatic cancer progression by acting as a ceRNA of mir-497-5p to upregulate expression of CCND1. Environ Toxicol 38(6):1251–1264. 10.1002/tox.23761 36947456 10.1002/tox.23761

[30] Bing Z, Han J, Zheng Z, Liang N (2021) FOXO3-induced oncogenic lncRNA CASC9 enhances gefitinib resistance of non-small-cell lung cancer through feedback loop. Life Sci 287:120012. 10.1016/j.lfs.2021.120012 34619168 10.1016/j.lfs.2021.120012

[31] Chen Z, Chen Q, Cheng Z et al (2020) Long non-coding RNA CASC9 promotes gefitinib resistance in NSCLC by epigenetic repression of DUSP1. Cell Death Dis 11(10):858. 10.1038/s41419-020-03047-y. Published 2020 Oct 14 33056982 10.1038/s41419-020-03047-y PMC7560854

[32] Zhang Z, Chen L, Zhao C et al (2023) CASC9 potentiates gemcitabine resistance in pancreatic cancer by reciprocally activating NRF2 and the NF-κB signaling pathway. Cell Biol Toxicol 39(4):1549–1560. 10.1007/s10565-022-09746-w 35913601 10.1007/s10565-022-09746-w

[33] Liu HZ, Shan TD, Han Y, Liu XS (2021) Silencing long non-coding RNA CASC9 inhibits colorectal cancer cell proliferation by acting as a competing endogenous RNA of miR-576-5p to regulate AKT3 [published correction appears in Cell Death Discov. ;7(1):185]. Cell Death Discov. 2020;6(1):115. Published 2020 Oct 31. 10.1038/s41420-020-00352-5 10.1038/s41420-020-00352-5 PMC7603495 33298846

[34] Zhang H, Wang J, Yu T, Wang J, Lu J, Yu Z (2022) Silencing LncRNA CASC9 inhibits proliferation and invasion of colorectal cancer cells by MiR-542-3p/ILK. PLoS ONE 17(4):e0265901 Published 2022 Apr 15. 10.1371/journal.pone.0265901 35427373 10.1371/journal.pone.0265901 PMC9012350

[35] Li C, Zhang K, Gong Y et al (2023) Based on cuproptosis-related lncRNAs, a novel prognostic signature for colon adenocarcinoma prognosis, immunotherapy, and chemotherapy response. Front Pharmacol 14:1200054 Published 2023 Jun 12. 10.3389/fphar.2023.1200054 37377924 10.3389/fphar.2023.1200054 PMC10291194

[36] Tang Q, Hu X, Guo Q, Shi Y, Liu L, Ying G (2022) Discovery and Validation of a Novel Metastasis-related lncRNA prognostic signature for Colorectal Cancer. Front Genet 13:704988. 10.3389/fgene.2022.704988. Published 2022 May 19 35664303 10.3389/fgene.2022.704988 PMC9162157

[37] Feng Q, Wang J, Cui N, Liu X, Wang H (2021) Autophagy-related long non-coding RNA signature for potential prognostic biomarkers of patients with cervical cancer: a study based on public databases. Ann Transl Med 9(22):1668. 10.21037/atm-21-5156 34988177 10.21037/atm-21-5156 PMC8667135

[38] Huang J, Jiang S, Liang L et al (2022) Analysis of PANoptosis-Related LncRNA-miRNA-mRNA Network Reveals LncRNA SNHG7 Involved in Chemo-Resistance in Colon Adenocarcinoma. Front Oncol. ;12:888105. Published 2022 May 12. 10.3389/fonc.2022.888105 10.3389/fonc.2022.888105 PMC9133343 35646635

[39] Pan D, Chen K, Chen P, Liu Y, Wu Y, Huang J (2023) Downregulation of LncRNA SNHG7 sensitizes colorectal Cancer cells to resist Anlotinib by regulating miR-181a-5p/GATA6. Gastroenterol Res Pract 2023:6973723 Published 2023 Jan 14. 10.1155/2023/6973723 36691432 10.1155/2023/6973723 PMC9867592

[40] Constantinidou A, Alifieris C, Trafalis DT (2019) Targeting programmed cell death – 1 (PD-1) and Ligand (PD-L1): a new era in cancer active immunotherapy. Pharmacol Ther 194:84–106. 10.1016/j.pharmthera.2018.09.008 30268773 10.1016/j.pharmthera.2018.09.008

[41] Hamada T, Cao Y, Qian ZR et al (2017) Aspirin Use and Colorectal Cancer Survival according to Tumor CD274 (programmed cell death 1 Ligand 1) expression status. J Clin Oncol 35(16):1836–1844. 10.1200/JCO.2016.70.7547 28406723 10.1200/JCO.2016.70.7547 PMC5455595

[42] Kennedy A, Waters E, Rowshanravan B et al (2022) Differences in CD80 and CD86 transendocytosis reveal CD86 as a key target for CTLA-4 immune regulation. Nat Immunol 23(9):1365–1378. 10.1038/s41590-022-01289-w 35999394 10.1038/s41590-022-01289-w PMC9477731

[43] Mirsepasi-Lauridsen HC, Vallance BA, Krogfelt KA, Petersen AM (2019) Escherichia coli Pathobionts Associated with Inflammatory Bowel Disease. Clin Microbiol Rev 32(2):e00060–e00018 Published 2019 Jan 30. 10.1128/CMR.00060-18 30700431 10.1128/CMR.00060-18 PMC6431131

[44] Li L, Liu X, Sanders KL et al (2019) TLR8-Mediated metabolic control of human Treg function: a mechanistic target for Cancer Immunotherapy. Cell Metab 29(1):103–123e5. 10.1016/j.cmet.2018.09.020 30344014 10.1016/j.cmet.2018.09.020 PMC7050437

[45] Liao X, Lochhead P, Nishihara R et al (2012) Aspirin use, tumor PIK3CA mutation, and colorectal-cancer survival. N Engl J Med 367(17):1596–1606. 10.1056/NEJMoa1207756 23094721 10.1056/NEJMoa1207756 PMC3532946

[46] Yaeger R, Weiss J, Pelster MS et al (2023) Adagrasib with or without Cetuximab in Colorectal Cancer with Mutated KRAS G12C. N Engl J Med 388(1):44–54. 10.1056/NEJMoa2212419 36546659 10.1056/NEJMoa2212419 PMC9908297

