# Supplementary material for: Disulfidptosis-related long non-coding RNA signature predicts the prognosis, tumor microenvironment, immunotherapy, and antitumor drug options in colon adenocarcinoma
Source: Apoptosis. 2024 Aug 8;29(11-12):2074–90. doi: 10.1007/s10495-024-02011-x (PMC11550253; doi:10.1007/s10495-024-02011-x)
Supplement: Supplementary file 2 — Supplementary Material 2 [file 10495_2024_2011_MOESM2_ESM.docx]

GYS1

NDUFS1

OXSM

LRPPRC

NDUFA11

NUBPL

NCKAP1

RPN1

SLC3A2

SLC7A11
