# Supplementary material for: Disulfidptosis-related long non-coding RNA signature predicts the prognosis, tumor microenvironment, immunotherapy, and antitumor drug options in colon adenocarcinoma
Source: Apoptosis. 2024 Aug 8;29(11-12):2074–90. doi: 10.1007/s10495-024-02011-x (PMC11550253; doi:10.1007/s10495-024-02011-x)
Supplement: Supplementary file 3 — Supplementary Material 3 [file 10495_2024_2011_MOESM3_ESM.docx]

Disulfidptosis lncRNA cor pvalue Regulation

NDUFA11 PXN-AS1 0.415909746 2.46E-21 postive

NCKAP1 AC068790.6 0.478804686 1.20E-28 postive

NCKAP1 AC005674.1 0.418844439 1.21E-21 postive

NCKAP1 CCDC18-AS1 0.401874085 6.68E-20 postive

NCKAP1 BACH1-IT1 0.433443493 3.19E-23 postive

SLC7A11 BACH1-IT1 0.403972499 4.12E-20 postive

NDUFA11 PITPNA-AS1 0.704629611 1.23E-72 postive

NCKAP1 AC246817.1 0.418355746 1.36E-21 postive

NCKAP1 AC068282.1 0.419221726 1.11E-21 postive

NCKAP1 AC084357.2 0.457325317 5.61E-26 postive

NCKAP1 LNCSRLR 0.512414857 3.17E-33 postive

NCKAP1 NFYC-AS1 0.420135894 8.85E-22 postive

NCKAP1 RTCA-AS1 0.528180235 1.49E-35 postive

RPN1 AC092301.1 -0.417479319 1.69E-21 negative

NCKAP1 LINC01719 0.413492504 4.40E-21 postive

NCKAP1 AC083900.1 0.413801566 4.09E-21 postive

NUBPL AC063960.1 0.406208619 2.45E-20 postive

NCKAP1 AC036108.3 0.446066886 1.19E-24 postive

NCKAP1 AC009704.2 0.407823455 1.68E-20 postive

NCKAP1 AC016074.2 0.459271366 3.27E-26 postive

SLC7A11 AC087893.2 0.405218438 3.09E-20 postive

SLC7A11 AC006460.2 0.450460855 3.66E-25 postive

NDUFA11 AC233992.3 0.450870004 3.28E-25 postive

NCKAP1 AC018647.2 0.406771209 2.15E-20 postive

NCKAP1 AL353708.3 0.44137288 4.10E-24 postive

NCKAP1 AL355472.3 0.432386491 4.18E-23 postive

NCKAP1 HCG25 0.427555745 1.41E-22 postive

NCKAP1 PITRM1-AS1 0.416174083 2.31E-21 postive

NCKAP1 MIR4435-2HG 0.431366688 5.41E-23 postive

SLC7A11 MIR4435-2HG 0.47239125 7.87E-28 postive

NCKAP1 AC022306.2 0.458782035 3.75E-26 postive

NCKAP1 AL354696.1 0.418460996 1.33E-21 postive

NCKAP1 AC093675.2 0.402399281 5.92E-20 postive

NCKAP1 AL360270.1 0.449330671 4.96E-25 postive

NCKAP1 AC046134.2 0.485309391 1.71E-29 postive

SLC7A11 AL606834.1 0.405637319 2.80E-20 postive

NCKAP1 CASC15 0.460954301 2.05E-26 postive

NDUFA11 Z84492.1 0.521690515 1.40E-34 postive

NCKAP1 AL109923.1 0.438986403 7.65E-24 postive

NDUFS1 AC097448.1 0.426415961 1.88E-22 postive

NCKAP1 AC097448.1 0.573848093 4.90E-43 postive

NCKAP1 AC134407.1 0.404092396 4.01E-20 postive

NCKAP1 MCPH1-AS1 0.452321137 2.21E-25 postive

SLC7A11 LINC01655 0.402114535 6.32E-20 postive

NCKAP1 AC008883.3 0.428253967 1.19E-22 postive

NCKAP1 FKBP14-AS1 0.452153004 2.31E-25 postive

NDUFA11 AL096870.12 0.677397115 3.35E-65 postive

NCKAP1 LINC02035 0.47547754 3.20E-28 postive

NCKAP1 AP000873.2 0.448865606 5.62E-25 postive

NCKAP1 LINC01572 0.469321167 1.91E-27 postive

NCKAP1 LURAP1L-AS1 0.436428489 1.48E-23 postive

NCKAP1 AL031118.1 0.411538984 7.00E-21 postive

NCKAP1 AC012360.1 0.44257553 2.99E-24 postive

NCKAP1 ATP11A-AS1 0.407158876 1.96E-20 postive

NCKAP1 C3orf35 0.403322346 4.79E-20 postive

NCKAP1 DIAPH2-AS1 0.429617362 8.42E-23 postive

NCKAP1 LINC00578 0.409874627 1.04E-20 postive

NCKAP1 AC138207.4 0.451028707 3.14E-25 postive

NCKAP1 RNF139-AS1 0.447975589 7.14E-25 postive

NCKAP1 AL355075.6 0.440398567 5.30E-24 postive

NDUFA11 DCST1-AS1 0.411006007 7.95E-21 postive

NCKAP1 AC072028.1 0.478303985 1.39E-28 postive

NCKAP1 RERG-IT1 0.446181248 1.15E-24 postive

NUBPL SP2-AS1 0.427958418 1.28E-22 postive

NCKAP1 SP2-AS1 0.536179178 8.79E-37 postive

NCKAP1 AC016949.1 0.43301959 3.55E-23 postive

NCKAP1 AC004893.2 0.402246316 6.14E-20 postive

NCKAP1 AP002336.2 0.434373868 2.52E-23 postive

NCKAP1 AL157838.1 0.405341339 3.00E-20 postive

NCKAP1 ALKBH3-AS1 0.406844646 2.11E-20 postive

NUBPL AC004816.1 0.444314671 1.89E-24 postive

NCKAP1 AC018926.1 0.420400718 8.29E-22 postive

NCKAP1 AC007684.2 0.428916177 1.00E-22 postive

SLC7A11 DARS-AS1 0.418924579 1.19E-21 postive

NCKAP1 C8orf44 0.490805178 3.18E-30 postive

NCKAP1 AC092338.1 0.403892442 4.20E-20 postive

SLC7A11 AC010198.2 0.449595307 4.62E-25 postive

NDUFA11 SNHG32 0.615335925 6.25E-51 postive

NCKAP1 AC068790.3 0.433558149 3.10E-23 postive

NCKAP1 AC090425.3 0.456877579 6.35E-26 postive

SLC7A11 AC039056.2 0.486842119 1.07E-29 postive

NCKAP1 AP000593.3 0.416029368 2.39E-21 postive

NCKAP1 AL359880.1 0.454040244 1.38E-25 postive

NUBPL SATB2-AS1 0.491628505 2.47E-30 postive

NCKAP1 AC023389.1 0.416022416 2.40E-21 postive

NCKAP1 AC073641.1 0.486079417 1.35E-29 postive

NCKAP1 AC023494.1 0.461661621 1.68E-26 postive

NCKAP1 AC026401.2 0.407540155 1.80E-20 postive

NCKAP1 AC124016.2 0.454502315 1.22E-25 postive

NCKAP1 AC008121.2 0.411483585 7.10E-21 postive

NCKAP1 AL031666.2 0.403003828 5.15E-20 postive

NCKAP1 AP001001.1 0.447282271 8.59E-25 postive

NCKAP1 AC114956.1 0.434350196 2.53E-23 postive

NCKAP1 AC027279.1 0.441193166 4.30E-24 postive

NCKAP1 AC012360.3 0.480777562 6.66E-29 postive

NCKAP1 PRANCR 0.413907786 3.98E-21 postive

SLC7A11 VAC14-AS1 0.409494089 1.14E-20 postive

NCKAP1 TMCC1-AS1 0.429853177 7.93E-23 postive

SLC7A11 LINC00862 0.423929909 3.48E-22 postive

NUBPL AC124067.4 0.447863471 7.35E-25 postive

NCKAP1 SNHG14 0.468550786 2.38E-27 postive

NCKAP1 AL359878.1 0.44129602 4.19E-24 postive

NCKAP1 MKLN1-AS 0.420733905 7.64E-22 postive

NUBPL CASC19 0.408422078 1.46E-20 postive

NCKAP1 CASC19 0.420225639 8.65E-22 postive

NCKAP1 AC018926.3 0.412797518 5.19E-21 postive

NCKAP1 SEMA6A-AS1 0.408384127 1.47E-20 postive

NCKAP1 AP000487.1 0.422492808 4.96E-22 postive

NCKAP1 PLCG1-AS1 0.437565395 1.11E-23 postive

NCKAP1 AC084824.5 0.423306579 4.06E-22 postive

NCKAP1 LINC01811 0.427902674 1.30E-22 postive

NCKAP1 CCNT2-AS1 0.484218353 2.37E-29 postive

NCKAP1 MORF4L2-AS1 0.449909617 4.24E-25 postive

NCKAP1 ARHGAP15-AS1 0.433110505 3.47E-23 postive

SLC7A11 AC079313.1 0.404067643 4.03E-20 postive

NCKAP1 AC004765.1 0.412183255 6.01E-21 postive

NCKAP1 AL138756.1 0.42424618 3.22E-22 postive

NCKAP1 AL683813.1 0.436898587 1.31E-23 postive

NCKAP1 AP000254.2 0.434264837 2.59E-23 postive

NDUFA11 AC026471.3 0.452635545 2.03E-25 postive

NCKAP1 AL035411.3 0.429891602 7.86E-23 postive

NCKAP1 AC007128.2 0.411376356 7.28E-21 postive

NCKAP1 SLC2A9-AS1 0.479807251 8.90E-29 postive

NCKAP1 AC092652.1 0.401785495 6.82E-20 postive

NCKAP1 AP000813.1 0.525038093 4.44E-35 postive

NCKAP1 AL592430.1 0.426538916 1.82E-22 postive

SLC3A2 AC126696.3 0.405278883 3.04E-20 postive

NCKAP1 AC025165.5 0.476560807 2.33E-28 postive

NCKAP1 EIF1AX-AS1 0.403621682 4.47E-20 postive

SLC7A11 AL360219.1 0.402089936 6.36E-20 postive

NCKAP1 ZBTB20-AS4 0.408201589 1.54E-20 postive

NCKAP1 AC120193.1 0.430724485 6.37E-23 postive

NCKAP1 TTC3-AS1 0.432876358 3.69E-23 postive

NCKAP1 AC011603.2 0.402698301 5.53E-20 postive

NDUFA11 AGAP2-AS1 0.627687781 1.63E-53 postive

NCKAP1 DUBR 0.526265534 2.90E-35 postive

NCKAP1 CFAP44-AS1 0.426940052 1.65E-22 postive

NCKAP1 PSMD6-AS2 0.417517059 1.67E-21 postive

NCKAP1 HCG18 0.517039064 6.78E-34 postive

NCKAP1 AC093382.1 0.443880113 2.12E-24 postive

NDUFS1 AC112220.2 0.412663265 5.36E-21 postive

NCKAP1 AC112220.2 0.557840885 2.79E-40 postive

NCKAP1 AL110115.1 0.419680209 9.89E-22 postive

NCKAP1 AL080317.2 0.465119241 6.34E-27 postive

NCKAP1 AP000977.1 0.419212585 1.11E-21 postive

NCKAP1 MIR4453HG 0.456437235 7.17E-26 postive

NCKAP1 SEC24B-AS1 0.543682134 5.77E-38 postive

NCKAP1 AC007823.1 0.50663008 2.12E-32 postive

SLC7A11 AL513327.1 0.417066936 1.86E-21 postive

NCKAP1 AC079804.3 0.506084223 2.53E-32 postive

SLC7A11 AC125437.1 0.473324376 6.00E-28 postive

NCKAP1 AL390195.1 0.421378652 6.53E-22 postive

SLC7A11 AC005224.3 0.456578817 6.89E-26 postive

NDUFA11 PPP1R14B-AS1 0.532545117 3.21E-36 postive

NDUFA11 AC010913.1 0.403586368 4.51E-20 postive

NCKAP1 AL354892.3 0.418386262 1.35E-21 postive

NCKAP1 AL136169.1 0.483610072 2.85E-29 postive

NCKAP1 AL022157.1 0.468026029 2.77E-27 postive

RPN1 AUXG01000058.1 -0.427728779 1.35E-22 negative

NUBPL WASL-DT 0.474301705 4.51E-28 postive

NCKAP1 WASL-DT 0.469146782 2.01E-27 postive

NCKAP1 AC083806.2 0.408359288 1.48E-20 postive

NCKAP1 AL731537.1 0.462461494 1.34E-26 postive

NCKAP1 AL157932.1 0.511471559 4.34E-33 postive

LRPPRC EMSLR 0.412084415 6.15E-21 postive

NCKAP1 SAP30L-AS1 0.412221869 5.96E-21 postive

OXSM MAPKAPK5-AS1 0.549100791 7.72E-39 postive

SLC7A11 CYTOR 0.497375323 4.10E-31 postive

NCKAP1 AC107214.1 0.432955805 3.61E-23 postive

NCKAP1 EDIL3-DT 0.434437063 2.47E-23 postive

NCKAP1 TPT1-AS1 0.40725503 1.92E-20 postive

NCKAP1 AL391684.1 0.452290175 2.23E-25 postive

NCKAP1 AC073332.1 0.466647499 4.10E-27 postive

NCKAP1 USP3-AS1 0.400005944 1.02E-19 postive

NCKAP1 AP002812.3 0.413334702 4.57E-21 postive

NCKAP1 AL158195.1 0.441130061 4.37E-24 postive

NCKAP1 LINC01355 0.462359763 1.38E-26 postive

NCKAP1 AC090739.1 0.450625711 3.50E-25 postive

NCKAP1 AC011815.1 0.487310895 9.28E-30 postive

NCKAP1 FRMD6-AS1 0.412899267 5.07E-21 postive

NCKAP1 A2M-AS1 0.446283886 1.12E-24 postive

NCKAP1 ZNF451-AS1 0.431723989 4.94E-23 postive

NCKAP1 AC026356.1 0.48900152 5.54E-30 postive

SLC7A11 AC026356.1 0.405081973 3.19E-20 postive

SLC7A11 AC008771.1 0.404274649 3.84E-20 postive

NCKAP1 AL158163.1 0.446845533 9.65E-25 postive

NCKAP1 AC092376.3 0.467827606 2.93E-27 postive

NCKAP1 AC023424.2 0.420927937 7.29E-22 postive

NCKAP1 AL365436.2 0.424808012 2.80E-22 postive

NCKAP1 AC007216.4 0.416222124 2.29E-21 postive

SLC7A11 SLC7A11-AS1 0.436680384 1.39E-23 postive

NCKAP1 AC108519.1 0.449784815 4.39E-25 postive

NCKAP1 N4BP2L2-IT2 0.451033536 3.13E-25 postive

NCKAP1 AC146507.3 0.442907452 2.74E-24 postive

NDUFA11 C5orf66-AS1 0.430314708 7.06E-23 postive

NUBPL CASC9 0.4330903 3.49E-23 postive

NCKAP1 AC096921.2 0.441612669 3.85E-24 postive

SLC7A11 AC107021.1 0.408437569 1.46E-20 postive

NCKAP1 ANKRD10-IT1 0.494793601 9.21E-31 postive

NCKAP1 ZEB1-AS1 0.408576481 1.41E-20 postive

NCKAP1 AP002907.1 0.412143935 6.07E-21 postive

NCKAP1 AL353801.1 0.414739366 3.26E-21 postive

NCKAP1 AC078883.1 0.49038996 3.61E-30 postive

NCKAP1 AC120053.1 0.466868147 3.85E-27 postive

NCKAP1 LINC02156 0.489448939 4.83E-30 postive

NDUFS1 OTUD6B-AS1 0.42936407 8.97E-23 postive

LRPPRC OTUD6B-AS1 0.418751808 1.24E-21 postive

NUBPL OTUD6B-AS1 0.534969167 1.36E-36 postive

NCKAP1 OTUD6B-AS1 0.603283851 1.62E-48 postive

NCKAP1 AC009299.2 0.490697247 3.29E-30 postive

NCKAP1 AC006230.1 0.435818603 1.74E-23 postive

NCKAP1 C2orf27A 0.54464667 4.04E-38 postive

NCKAP1 ZNF460-AS1 0.418397524 1.35E-21 postive

NDUFA11 AC018761.2 0.507645166 1.52E-32 postive

NCKAP1 AL137246.1 0.451537212 2.73E-25 postive

NCKAP1 AC005856.1 0.441835555 3.64E-24 postive

NCKAP1 AC022306.3 0.458482364 4.07E-26 postive

NUBPL REPIN1-AS1 0.445466803 1.39E-24 postive

NCKAP1 AL591848.3 0.406873802 2.10E-20 postive

NCKAP1 AC009032.1 0.431384851 5.39E-23 postive

NCKAP1 AC020891.3 0.412365634 5.76E-21 postive

NUBPL AC234917.3 0.416937256 1.92E-21 postive

SLC3A2 AC234917.3 0.442739781 2.87E-24 postive

NCKAP1 AC021851.1 0.512152582 3.46E-33 postive

NDUFS1 AC006008.1 0.422356546 5.13E-22 postive

NCKAP1 AC006008.1 0.484654594 2.08E-29 postive

NCKAP1 AC093297.2 0.470162016 1.50E-27 postive

NCKAP1 LINC01359 0.434761757 2.28E-23 postive

NUBPL LINC00654 0.439301856 7.05E-24 postive

NCKAP1 LINC00654 0.42111149 6.97E-22 postive

NUBPL LINC01184 0.472487137 7.65E-28 postive

NCKAP1 LINC01184 0.421544728 6.27E-22 postive

SLC7A11 AP006259.1 0.420876122 7.38E-22 postive

NCKAP1 AC105285.1 0.475884444 2.84E-28 postive

NCKAP1 AL445223.1 0.430439374 6.84E-23 postive

SLC7A11 MSC-AS1 0.405316027 3.02E-20 postive

NUBPL AC093599.2 0.426344134 1.91E-22 postive

NDUFA11 AL445524.1 0.408146654 1.56E-20 postive

NCKAP1 PSPC1-AS2 0.43411531 2.69E-23 postive

NCKAP1 AC007681.1 0.480218191 7.87E-29 postive

NCKAP1 KLHL7-DT 0.40324246 4.88E-20 postive

NDUFA11 AC018645.3 -0.490603207 3.38E-30 negative

NCKAP1 AC018645.3 0.526987539 2.26E-35 postive

NCKAP1 AC012467.2 0.529347254 9.90E-36 postive

NDUFS1 FAM160A1-DT 0.469496687 1.82E-27 postive

NUBPL FAM160A1-DT 0.458731982 3.80E-26 postive

NCKAP1 FAM160A1-DT 0.494841943 9.08E-31 postive

NCKAP1 AC107081.3 0.450813041 3.33E-25 postive

NCKAP1 AL049552.1 0.429722119 8.20E-23 postive

SLC7A11 ARNTL2-AS1 0.422679616 4.74E-22 postive

NCKAP1 AL358072.1 0.446607315 1.03E-24 postive

NCKAP1 ITGA9-AS1 0.446933742 9.43E-25 postive

NUBPL AC010148.1 0.413705292 4.18E-21 postive

NCKAP1 AC010148.1 0.442481184 3.07E-24 postive

NUBPL GASAL1 0.402513123 5.77E-20 postive

SLC3A2 GASAL1 0.449987341 4.16E-25 postive

NDUFA11 AC137630.3 0.402181784 6.23E-20 postive

NCKAP1 ARHGEF35-AS1 0.463402899 1.03E-26 postive

NCKAP1 AL135902.1 0.441631418 3.84E-24 postive

NCKAP1 AL031667.3 0.49054304 3.45E-30 postive

NCKAP1 RBM26-AS1 0.543966132 5.19E-38 postive

NCKAP1 AC092802.3 0.440463982 5.21E-24 postive

NCKAP1 AL731566.2 0.476066014 2.69E-28 postive

NDUFS1 NNT-AS1 0.41123364 7.53E-21 postive

NDUFA11 NNT-AS1 -0.408958242 1.29E-20 negative

NUBPL NNT-AS1 0.518323362 4.40E-34 postive

NCKAP1 NNT-AS1 0.5729629 7.02E-43 postive

NCKAP1 AC108471.3 0.411470177 7.12E-21 postive

NUBPL AL355922.4 0.432504487 4.05E-23 postive

NCKAP1 UBA6-AS1 0.445466392 1.39E-24 postive

NCKAP1 AL031666.1 0.43634756 1.51E-23 postive

NCKAP1 AC104564.5 0.437545595 1.11E-23 postive

NCKAP1 AC114956.2 0.485437441 1.64E-29 postive

NCKAP1 AC048341.1 0.427472595 1.44E-22 postive

NCKAP1 AC008453.2 0.40084819 8.46E-20 postive

NCKAP1 AC112503.2 0.473630164 5.49E-28 postive

NCKAP1 AC005165.1 0.454009252 1.40E-25 postive

NCKAP1 AC008124.1 0.577850694 9.49E-44 postive

SLC7A11 AC137932.2 0.445817999 1.27E-24 postive

NCKAP1 AL354696.2 0.519811655 2.66E-34 postive

NCKAP1 AC103591.4 0.553697887 1.36E-39 postive

NCKAP1 AL109614.1 0.488567511 6.32E-30 postive

NCKAP1 MIR17HG 0.499769781 1.92E-31 postive

NDUFA11 AC067852.2 0.485956168 1.40E-29 postive

SLC7A11 MAP3K5-AS1 0.429144623 9.48E-23 postive

NCKAP1 AC073333.1 0.465694912 5.38E-27 postive

NCKAP1 AC095055.1 0.476619418 2.29E-28 postive

NCKAP1 LINC00412 0.444466151 1.82E-24 postive

NCKAP1 AL590064.1 0.428948075 9.96E-23 postive

NCKAP1 AC011405.1 0.419905947 9.36E-22 postive

SLC7A11 RHOQ-AS1 0.410688118 8.57E-21 postive

NUBPL LINC01091 0.406973185 2.05E-20 postive

NCKAP1 AC087521.2 0.400108582 1.00E-19 postive

NCKAP1 ADAMTS9-AS1 0.445105681 1.53E-24 postive

NCKAP1 ENTPD1-AS1 0.450792897 3.34E-25 postive

NCKAP1 AC019330.1 0.451376794 2.86E-25 postive

NCKAP1 LINC00571 0.538254113 4.17E-37 postive

NCKAP1 AL512656.1 0.417954266 1.50E-21 postive

NCKAP1 AC048344.4 0.475173861 3.49E-28 postive

SLC7A11 AC048344.4 0.406348909 2.37E-20 postive

NCKAP1 AC068790.2 0.474597552 4.14E-28 postive

NCKAP1 LINC02603 0.433178627 3.41E-23 postive

NCKAP1 SGMS1-AS1 0.561214912 7.53E-41 postive

SLC7A11 AC022973.5 0.403355434 4.75E-20 postive

NCKAP1 AL139383.1 0.405373864 2.98E-20 postive

NCKAP1 AC092164.1 0.42309392 4.28E-22 postive

NCKAP1 AC023632.5 0.478722842 1.23E-28 postive

NCKAP1 AC009948.1 0.421069586 7.04E-22 postive

NCKAP1 TMEM9B-AS1 0.430968838 5.98E-23 postive

NCKAP1 AL390957.1 0.408211329 1.54E-20 postive

NCKAP1 AL391834.1 0.4644269 7.71E-27 postive

NCKAP1 AL355075.2 0.418912327 1.19E-21 postive

NCKAP1 AC004492.1 0.456463394 7.12E-26 postive

NCKAP1 LAMTOR5-AS1 0.534654905 1.52E-36 postive

NCKAP1 AC022400.4 0.485017886 1.86E-29 postive

NCKAP1 DNAJC3-DT 0.451595235 2.69E-25 postive

NCKAP1 AL139082.1 0.43113733 5.73E-23 postive

NCKAP1 BCDIN3D-AS1 0.439246113 7.15E-24 postive

NDUFS1 Z68871.1 0.420175414 8.76E-22 postive

NCKAP1 Z68871.1 0.574170453 4.30E-43 postive

NUBPL AC079160.1 0.40848368 1.44E-20 postive

NCKAP1 AC079160.1 0.421440312 6.43E-22 postive

NUBPL AC064807.1 0.433765918 2.94E-23 postive

NUBPL AC012184.3 0.425205627 2.54E-22 postive

NCKAP1 MCM3AP-AS1 0.512512828 3.07E-33 postive

NCKAP1 AC005726.2 0.428783704 1.04E-22 postive

NUBPL HAS2-AS1 0.442179474 3.32E-24 postive

SLC7A11 MIR223HG 0.406955764 2.06E-20 postive

SLC7A11 AC133644.1 0.413622949 4.26E-21 postive

NCKAP1 AC018695.4 0.403197887 4.93E-20 postive

NCKAP1 AC018628.2 0.438883838 7.86E-24 postive

NCKAP1 BAALC-AS1 0.512075697 3.55E-33 postive

NCKAP1 ZNF337-AS1 0.499764581 1.92E-31 postive

NCKAP1 AC012181.1 0.427602366 1.40E-22 postive

NCKAP1 GNG12-AS1 0.452140873 2.32E-25 postive

NUBPL CASC2 0.402861369 5.33E-20 postive

NCKAP1 CASC2 0.495307145 7.85E-31 postive

NCKAP1 AC006042.3 0.423604974 3.77E-22 postive

NUBPL AC100814.1 0.435447307 1.91E-23 postive

NCKAP1 AC100814.1 0.407648138 1.75E-20 postive

NCKAP1 AC093788.1 0.462214752 1.44E-26 postive

SLC7A11 GSEC 0.450671695 3.46E-25 postive

SLC7A11 AL442067.1 0.404471772 3.67E-20 postive

NDUFS1 AC107027.3 0.456650123 6.76E-26 postive

NUBPL AC107027.3 0.408351675 1.49E-20 postive

NCKAP1 AC107027.3 0.601897271 3.02E-48 postive

NCKAP1 AC097634.1 0.422216437 5.31E-22 postive

NCKAP1 AC073073.2 0.549323219 7.11E-39 postive

NCKAP1 AC006504.7 0.426341027 1.91E-22 postive

NDUFA11 ZNF236-DT 0.4413383 4.14E-24 postive

NCKAP1 AL031770.1 0.440042283 5.81E-24 postive

NCKAP1 MACC1-AS1 0.452341913 2.20E-25 postive

NCKAP1 AC004594.1 0.405347742 3.00E-20 postive

NCKAP1 AC006017.1 0.438883559 7.86E-24 postive

NCKAP1 LINC00216 0.400541351 9.07E-20 postive

NCKAP1 AC019186.1 0.463351469 1.04E-26 postive

SLC7A11 AP000331.1 0.407800474 1.69E-20 postive

NCKAP1 AC092794.1 0.440390967 5.31E-24 postive

NCKAP1 AC006213.7 0.42412036 3.32E-22 postive

NCKAP1 AC090517.2 0.483602764 2.86E-29 postive

NDUFA11 AC008443.4 0.671647839 9.85E-64 postive

NCKAP1 AC124862.1 0.422865671 4.53E-22 postive

NCKAP1 WDFY3-AS2 0.576459188 1.68E-43 postive

SLC3A2 AC104958.2 0.403817512 4.27E-20 postive

NCKAP1 AC007384.1 0.430097439 7.46E-23 postive

NCKAP1 AC093690.1 0.413132097 4.79E-21 postive

NCKAP1 SOS1-IT1 0.460708092 2.19E-26 postive

NCKAP1 AC010422.4 0.425460478 2.38E-22 postive

NCKAP1 ALMS1-IT1 0.439715171 6.33E-24 postive

NCKAP1 AL596223.1 0.413202505 4.72E-21 postive

NCKAP1 INO80-AS1 0.405183165 3.11E-20 postive

NCKAP1 TMC3-AS1 0.430939721 6.03E-23 postive

NCKAP1 AC243773.2 0.412610561 5.43E-21 postive

NCKAP1 AL132989.1 0.419543686 1.02E-21 postive

NCKAP1 LDLRAD4-AS1 0.406047593 2.55E-20 postive

NCKAP1 ITFG1-AS1 0.467718104 3.02E-27 postive

NCKAP1 AC004837.4 0.471385854 1.05E-27 postive

NCKAP1 AC024075.1 0.506724127 2.05E-32 postive

NCKAP1 AL590101.1 0.42959045 8.48E-23 postive

NCKAP1 LINC00539 0.435390924 1.94E-23 postive

NCKAP1 AL161891.1 0.49164788 2.45E-30 postive

NCKAP1 AL158163.2 0.479621983 9.40E-29 postive

NCKAP1 AC010834.3 0.530678119 6.21E-36 postive

SLC7A11 RDH10-AS1 0.401523192 7.24E-20 postive

NCKAP1 AC026356.2 0.43521314 2.03E-23 postive

NCKAP1 B4GALT4-AS1 0.445750148 1.29E-24 postive

NCKAP1 AC139019.1 0.477422535 1.80E-28 postive

NCKAP1 FTX 0.469467203 1.83E-27 postive

NCKAP1 TMEM202-AS1 0.43221669 4.36E-23 postive

NCKAP1 RASA2-IT1 0.40955406 1.12E-20 postive

NCKAP1 AC093535.1 0.401508372 7.27E-20 postive

NCKAP1 GLYCTK-AS1 0.423103237 4.27E-22 postive

NCKAP1 AC021078.1 0.479199172 1.07E-28 postive

NCKAP1 AC104984.2 0.413932141 3.96E-21 postive

NCKAP1 RNASEH2B-AS1 0.443434609 2.39E-24 postive

SLC7A11 LINC00622 0.450837651 3.30E-25 postive

NCKAP1 PABPC4-AS1 0.434501353 2.43E-23 postive

NCKAP1 FMR1-IT1 0.423341293 4.03E-22 postive

SLC7A11 AL596325.2 0.422321323 5.18E-22 postive

NCKAP1 AC019080.5 0.534065827 1.87E-36 postive

NCKAP1 AC005052.2 0.51530961 1.21E-33 postive

NCKAP1 AC034139.1 0.528815613 1.19E-35 postive

NCKAP1 AC007128.1 0.494047358 1.16E-30 postive

NDUFA11 LINC01106 -0.406334915 2.38E-20 negative

NUBPL LINC01106 0.40212265 6.31E-20 postive

NDUFS1 OIP5-AS1 0.50332941 6.16E-32 postive

NDUFA11 OIP5-AS1 -0.423438016 3.93E-22 negative

NCKAP1 OIP5-AS1 0.649217509 2.62E-58 postive

SLC7A11 OIP5-AS1 0.419043339 1.15E-21 postive

NCKAP1 ACSL3-AS1 0.467570263 3.15E-27 postive

SLC7A11 AC010542.5 0.401224892 7.76E-20 postive

NCKAP1 AL355488.1 0.415471757 2.74E-21 postive

NCKAP1 AL161430.1 0.432183893 4.40E-23 postive

NCKAP1 AC106845.1 0.435120839 2.08E-23 postive

NCKAP1 MED4-AS1 0.420234479 8.64E-22 postive

NCKAP1 RNF32-AS1 0.464517941 7.51E-27 postive

NUBPL AC034229.4 0.400858055 8.44E-20 postive

NCKAP1 AC034229.4 0.408803198 1.34E-20 postive

NCKAP1 AL353804.2 0.466106005 4.79E-27 postive

NCKAP1 AC025188.1 0.423784462 3.61E-22 postive

NCKAP1 AC084782.3 0.410830618 8.28E-21 postive

NCKAP1 STAM-AS1 0.400127875 9.97E-20 postive

NDUFS1 AC011477.2 0.429668421 8.31E-23 postive

NCKAP1 AC011477.2 0.52163966 1.43E-34 postive

SLC7A11 GK-AS1 0.415461875 2.74E-21 postive

NCKAP1 AC099565.1 0.461371504 1.82E-26 postive

NUBPL AC093227.3 0.429923798 7.79E-23 postive

NCKAP1 AC093227.3 0.474028116 4.89E-28 postive

NCKAP1 LMO7-AS1 0.444545788 1.78E-24 postive

NCKAP1 MIR133A1HG 0.408666302 1.38E-20 postive

NCKAP1 ZRANB2-AS1 0.481005344 6.23E-29 postive

NCKAP1 AL390195.2 0.446733859 9.95E-25 postive

NCKAP1 AC113139.1 0.449478298 4.77E-25 postive

NCKAP1 PSMA3-AS1 0.503760634 5.36E-32 postive

NCKAP1 AL110114.1 0.40772274 1.72E-20 postive

NCKAP1 AC092376.2 0.472200967 8.31E-28 postive

SLC7A11 HCG17 0.401794675 6.81E-20 postive

NDUFS1 SMG7-AS1 0.402245555 6.14E-20 postive

NCKAP1 SMG7-AS1 0.443611627 2.28E-24 postive

NCKAP1 AL080317.1 0.445193356 1.50E-24 postive

NCKAP1 AL020997.2 0.40813692 1.56E-20 postive

NCKAP1 AC018648.1 0.400303914 9.58E-20 postive

NCKAP1 PHACTR2-AS1 0.406035728 2.55E-20 postive

NCKAP1 AC069243.1 0.419047789 1.15E-21 postive

NCKAP1 AC099811.5 0.403249375 4.87E-20 postive

NCKAP1 LINC01409 0.433968751 2.79E-23 postive

NCKAP1 AC022150.4 0.428882584 1.01E-22 postive

NCKAP1 FAM13A-AS1 0.437036792 1.27E-23 postive

LRPPRC SNHG16 0.508808375 1.04E-32 postive

NCKAP1 SNHG16 0.430451239 6.82E-23 postive

NCKAP1 AL929236.1 0.418658116 1.27E-21 postive

NCKAP1 TNFRSF10A-AS1 0.454466305 1.23E-25 postive

SLC7A11 TNFRSF10A-AS1 0.434257123 2.59E-23 postive

NCKAP1 CHRM3-AS2 0.428723414 1.05E-22 postive

NUBPL AC021321.1 0.444646198 1.73E-24 postive

NCKAP1 AC021321.1 0.435753509 1.77E-23 postive

NCKAP1 AL096701.3 0.426813153 1.70E-22 postive

NCKAP1 AL139274.2 0.462438528 1.35E-26 postive

NCKAP1 PAXBP1-AS1 0.462070832 1.50E-26 postive

NCKAP1 Z99289.3 0.44137766 4.10E-24 postive

NCKAP1 AL137003.1 0.487326906 9.24E-30 postive

NCKAP1 AL359922.2 0.414687176 3.31E-21 postive

NCKAP1 AL109984.1 0.50713605 1.80E-32 postive

NUBPL AC073140.2 0.415685656 2.60E-21 postive

NDUFS1 FGD5-AS1 0.50201243 9.39E-32 postive

OXSM FGD5-AS1 0.441002119 4.52E-24 postive

LRPPRC FGD5-AS1 0.440474228 5.19E-24 postive

NUBPL FGD5-AS1 0.428684996 1.06E-22 postive

NCKAP1 FGD5-AS1 0.625093294 5.81E-53 postive

NCKAP1 AC017116.2 0.408119575 1.57E-20 postive

NCKAP1 AC004076.2 0.421298061 6.66E-22 postive

NCKAP1 AC079142.1 0.49402666 1.17E-30 postive

NCKAP1 AC004943.3 0.456302008 7.44E-26 postive

NCKAP1 AC108463.3 0.403777396 4.31E-20 postive

SLC7A11 AL592148.3 0.439357294 6.95E-24 postive

NCKAP1 AL596202.1 0.450338449 3.78E-25 postive

NDUFS1 AC098656.1 0.46701094 3.70E-27 postive

NUBPL AC098656.1 0.514175729 1.77E-33 postive

NCKAP1 AC098656.1 0.507980696 1.36E-32 postive

NCKAP1 AC073349.4 0.411092216 7.79E-21 postive

NCKAP1 AC004593.1 0.410088209 9.87E-21 postive

NCKAP1 AC124283.3 0.423555576 3.82E-22 postive

SLC7A11 AC121761.1 0.412427901 5.67E-21 postive

NCKAP1 CCAT2 0.403903557 4.19E-20 postive

SLC7A11 AC009682.1 0.403021601 5.13E-20 postive

SLC3A2 VPS9D1-AS1 0.462802769 1.22E-26 postive

NCKAP1 AL049539.1 0.426310792 1.93E-22 postive

NCKAP1 LAMC1-AS1 0.439411045 6.85E-24 postive

NCKAP1 Z99572.1 0.551905146 2.69E-39 postive

NCKAP1 AL117344.2 0.400558156 9.04E-20 postive

NCKAP1 AC087501.4 0.437573561 1.10E-23 postive

SLC7A11 AC087501.4 0.440976152 4.55E-24 postive

NCKAP1 AL161663.2 0.41059736 8.75E-21 postive

NCKAP1 AP000704.1 0.416603963 2.08E-21 postive

NCKAP1 AP002449.1 0.508658553 1.09E-32 postive

SLC7A11 AP002449.1 0.427140478 1.57E-22 postive

NCKAP1 AC001226.1 0.419686801 9.87E-22 postive

NCKAP1 AC005034.5 0.452805276 1.94E-25 postive

NCKAP1 AL442128.2 0.454231983 1.31E-25 postive

NCKAP1 AC078846.1 0.412198334 5.99E-21 postive

NCKAP1 AL157400.4 0.512554764 3.03E-33 postive

NCKAP1 AC009812.3 0.540156418 2.09E-37 postive

NCKAP1 ADNP-AS1 0.506382331 2.30E-32 postive

NCKAP1 AL031775.2 0.414932348 3.12E-21 postive

NCKAP1 LINC00630 0.506782046 2.02E-32 postive

NCKAP1 FAM66C 0.458315892 4.27E-26 postive

NCKAP1 AC087284.1 0.412570074 5.48E-21 postive

NDUFS1 AC006504.5 0.430439636 6.84E-23 postive

NUBPL AC006504.5 0.439435216 6.81E-24 postive

NCKAP1 AC006504.5 0.511741827 3.97E-33 postive

LRPPRC KTN1-AS1 0.516538154 8.02E-34 postive

NUBPL KTN1-AS1 0.602680278 2.12E-48 postive

NCKAP1 AC093752.2 0.450735997 3.40E-25 postive

NDUFA11 AL023803.1 0.439891899 6.04E-24 postive

NCKAP1 KANSL1L-AS1 0.477012441 2.04E-28 postive

NCKAP1 ALG13-AS1 0.457618967 5.17E-26 postive

SLC7A11 POLH-AS1 0.40256571 5.70E-20 postive

NCKAP1 AC017071.1 0.464563498 7.42E-27 postive

NCKAP1 GARS1-DT 0.450858125 3.29E-25 postive

NCKAP1 AC006525.1 0.444899224 1.62E-24 postive

NCKAP1 AC009812.4 0.462867059 1.20E-26 postive

NCKAP1 AC005332.7 0.42914779 9.48E-23 postive

NCKAP1 AC004943.1 0.459871303 2.77E-26 postive

NDUFS1 AC007637.1 0.410424166 9.12E-21 postive

NCKAP1 AC007637.1 0.476860876 2.13E-28 postive

NCKAP1 AC107068.1 0.525104881 4.33E-35 postive

SLC7A11 MANCR 0.426725349 1.74E-22 postive

NDUFA11 ATP2A1-AS1 0.504923497 3.68E-32 postive

NCKAP1 LINC01545 0.409137605 1.24E-20 postive

NCKAP1 AC007390.1 0.442731597 2.87E-24 postive

NCKAP1 AC096741.1 0.418143683 1.44E-21 postive

NCKAP1 HYMAI 0.45620725 7.64E-26 postive

NCKAP1 ZNF790-AS1 0.402924206 5.25E-20 postive

NCKAP1 AC100821.2 0.47269096 7.21E-28 postive

NCKAP1 Z98884.2 0.40996621 1.02E-20 postive

SLC7A11 AC024909.1 0.45663749 6.78E-26 postive

SLC7A11 AC002550.2 0.429403161 8.89E-23 postive

NCKAP1 AC104964.3 0.429600063 8.46E-23 postive

NCKAP1 MIATNB 0.447156213 8.89E-25 postive

NCKAP1 AL354993.1 0.448767939 5.77E-25 postive

NCKAP1 AL133243.3 0.465865182 5.13E-27 postive

NCKAP1 AC068620.2 0.451813755 2.54E-25 postive

NCKAP1 AL138962.1 0.409594997 1.11E-20 postive

NCKAP1 AC008906.1 0.453065895 1.80E-25 postive

SLC3A2 AC109322.1 0.423851793 3.55E-22 postive

NDUFA11 SNHG9 0.520914568 1.83E-34 postive

NCKAP1 AC093157.1 0.435298063 1.98E-23 postive

SLC7A11 AC010618.3 0.409358579 1.17E-20 postive

NCKAP1 AC010260.1 0.438926548 7.77E-24 postive

NCKAP1 LINC00339 0.408599708 1.40E-20 postive

NCKAP1 AC105339.2 0.451135224 3.05E-25 postive

NCKAP1 THUMPD3-AS1 0.479459717 9.87E-29 postive

SLC7A11 AC005632.3 0.417751491 1.58E-21 postive

NUBPL AF241728.2 0.456880235 6.34E-26 postive

NCKAP1 AF241728.2 0.423131178 4.24E-22 postive

NDUFS1 LINC01806 0.476252231 2.55E-28 postive

LRPPRC LINC01806 0.41036623 9.25E-21 postive

NUBPL LINC01806 0.407114185 1.99E-20 postive

NCKAP1 LINC01806 0.522217672 1.17E-34 postive

SLC3A2 MRPL20-AS1 0.421169883 6.87E-22 postive

NDUFA11 AP001160.2 0.434461095 2.46E-23 postive

NCKAP1 AC012313.5 0.417573951 1.65E-21 postive

NCKAP1 AL590723.1 0.415659473 2.62E-21 postive

NCKAP1 AL355312.2 0.430839858 6.18E-23 postive

NDUFA11 SNHG25 0.545892977 2.55E-38 postive

NCKAP1 AC130650.2 0.454713797 1.15E-25 postive

NCKAP1 AC079915.1 0.43077673 6.28E-23 postive

NCKAP1 AC110611.1 0.490560262 3.43E-30 postive

NCKAP1 AC019080.1 0.527109512 2.16E-35 postive

NCKAP1 AC040904.1 0.443273353 2.49E-24 postive

SLC7A11 AC011092.3 0.406587687 2.24E-20 postive

NCKAP1 AC010168.2 0.484366579 2.27E-29 postive

NCKAP1 AC079907.1 0.472155358 8.43E-28 postive

NUBPL AC007255.1 0.428109443 1.23E-22 postive

NUBPL AC239584.1 0.479058337 1.11E-28 postive

NCKAP1 AC021205.3 0.426896752 1.67E-22 postive

NCKAP1 AC110813.1 0.40071096 8.72E-20 postive

NCKAP1 AC005070.3 0.452723379 1.98E-25 postive

NUBPL AC011773.3 0.412991903 4.96E-21 postive

NCKAP1 AC099811.1 0.435380392 1.94E-23 postive

NCKAP1 TRAF3IP2-AS1 0.481120877 6.01E-29 postive

OXSM AC083855.2 0.450161909 3.97E-25 postive

NCKAP1 AL596247.1 0.419665146 9.92E-22 postive

NCKAP1 AC131971.1 0.409769898 1.06E-20 postive

NCKAP1 MANEA-DT 0.494879219 8.97E-31 postive

NCKAP1 AL732509.1 0.511307296 4.58E-33 postive

NUBPL WARS2-AS1 0.439314066 7.03E-24 postive

NCKAP1 WARS2-AS1 0.515674399 1.07E-33 postive

NCKAP1 AC138393.3 0.461126713 1.95E-26 postive

NCKAP1 AL731563.3 0.449752301 4.43E-25 postive

NDUFS1 IQCH-AS1 0.424981377 2.68E-22 postive

NUBPL IQCH-AS1 0.421672445 6.07E-22 postive

NCKAP1 IQCH-AS1 0.57324542 6.26E-43 postive

NCKAP1 AL035448.1 0.403687474 4.40E-20 postive

NCKAP1 AC004908.2 0.446807646 9.75E-25 postive

SLC7A11 AC114760.2 0.405204668 3.10E-20 postive

NCKAP1 AL359643.2 0.458847293 3.68E-26 postive

NCKAP1 LINC01473 0.502424789 8.23E-32 postive

NCKAP1 MIRLET7A1HG 0.406544586 2.27E-20 postive

NCKAP1 AC073896.3 0.439398949 6.87E-24 postive

NCKAP1 AC110769.2 0.410069544 9.92E-21 postive

NCKAP1 AL354813.1 0.401517504 7.25E-20 postive

NCKAP1 AC009090.6 0.463355139 1.04E-26 postive

NCKAP1 AF230666.1 0.492260653 2.03E-30 postive

NUBPL PCAT1 0.423062279 4.31E-22 postive

NCKAP1 PCAT1 0.425388572 2.43E-22 postive

NCKAP1 AC012213.3 0.469676681 1.72E-27 postive

NCKAP1 MRPS9-AS1 0.439831489 6.14E-24 postive

SLC7A11 AF124730.1 0.427795504 1.33E-22 postive

NDUFA11 AC020663.2 0.483879999 2.63E-29 postive

NCKAP1 AC007535.1 0.401558785 7.18E-20 postive

NCKAP1 AF129075.1 0.495169931 8.19E-31 postive

NCKAP1 ZKSCAN7-AS1 0.507026592 1.86E-32 postive

NCKAP1 SAMD12-AS1 0.445316922 1.45E-24 postive

NCKAP1 MALINC1 0.432689685 3.87E-23 postive

NDUFS1 NORAD 0.417725692 1.59E-21 postive

NUBPL NORAD 0.455920124 8.26E-26 postive

NCKAP1 NORAD 0.639992397 3.31E-56 postive

NCKAP1 AL121583.1 0.406142555 2.49E-20 postive

NCKAP1 Z94721.3 0.473203299 6.21E-28 postive

NCKAP1 RAB30-DT 0.493876128 1.23E-30 postive

NCKAP1 AL355073.1 0.464758289 7.02E-27 postive

NCKAP1 AC007066.2 0.430281055 7.12E-23 postive

NCKAP1 UGDH-AS1 0.429910245 7.82E-23 postive

NCKAP1 CRTC3-AS1 0.48049654 7.25E-29 postive

NCKAP1 AC109992.2 0.482647697 3.81E-29 postive

NCKAP1 AL157392.4 0.413873927 4.02E-21 postive

NUBPL AC005280.1 0.479983009 8.45E-29 postive

NDUFA11 SNHG21 0.576837582 1.44E-43 postive

NCKAP1 AL132780.1 0.42544673 2.39E-22 postive

NCKAP1 AC022079.1 0.469787851 1.67E-27 postive

LRPPRC FAM222A-AS1 0.544214427 4.74E-38 postive

NDUFS1 LINC00662 0.405810751 2.69E-20 postive

NUBPL LINC00662 0.499468714 2.11E-31 postive

NCKAP1 LINC00662 0.594928336 6.64E-47 postive

NCKAP1 AC016405.1 0.413633585 4.25E-21 postive

NCKAP1 AC000123.1 0.52680907 2.40E-35 postive

NCKAP1 SBF2-AS1 0.438815893 8.00E-24 postive

NUBPL LINC02175 0.424582658 2.96E-22 postive

NCKAP1 LINC02175 0.441938121 3.54E-24 postive

NCKAP1 AP001442.1 0.442903321 2.75E-24 postive

NCKAP1 LINC02163 0.43085046 6.17E-23 postive

NCKAP1 AL162734.1 0.453052095 1.81E-25 postive

NCKAP1 AL590282.2 0.441873874 3.60E-24 postive

NCKAP1 MAGI2-AS3 0.423908878 3.50E-22 postive

NCKAP1 AC004832.4 0.430236258 7.20E-23 postive

NCKAP1 AC025917.1 0.428091579 1.24E-22 postive

NCKAP1 AC008537.2 0.471233922 1.10E-27 postive

NDUFA11 AC010997.5 0.447697312 7.69E-25 postive

NCKAP1 LINC-PINT 0.42818854 1.21E-22 postive

NCKAP1 AC008456.1 0.456747229 6.58E-26 postive

NCKAP1 SMYD3-IT1 0.404780863 3.42E-20 postive

NDUFA11 AC020765.2 0.419072546 1.15E-21 postive

NCKAP1 AC016394.1 0.448728716 5.83E-25 postive

NDUFS1 AC073254.1 0.420671964 7.76E-22 postive

NCKAP1 AC073254.1 0.500559036 1.49E-31 postive

NCKAP1 AP001893.1 0.444615153 1.75E-24 postive

NCKAP1 AC053513.2 0.450619536 3.50E-25 postive

NCKAP1 AC011773.4 0.407820287 1.68E-20 postive

NCKAP1 CNOT10-AS1 0.457767186 4.97E-26 postive

NCKAP1 AL034405.1 0.402777565 5.43E-20 postive

NCKAP1 AC007336.1 0.43631075 1.53E-23 postive

NCKAP1 LINC01376 0.462349177 1.38E-26 postive

SLC7A11 AC107308.1 0.402747578 5.47E-20 postive

NCKAP1 INTS6-AS1 0.421375562 6.53E-22 postive

NCKAP1 LINC02615 0.431847588 4.79E-23 postive

NCKAP1 AC012676.3 0.427725072 1.35E-22 postive

NCKAP1 AC025682.1 0.433424144 3.21E-23 postive

NCKAP1 AC024060.2 0.412434128 5.66E-21 postive

NCKAP1 AC007881.4 0.430855556 6.16E-23 postive

NCKAP1 AC087854.1 0.439854817 6.10E-24 postive

NCKAP1 AP000866.6 0.435062999 2.11E-23 postive

NCKAP1 AL121895.1 0.430724766 6.37E-23 postive

NCKAP1 FBXO30-DT 0.504807381 3.82E-32 postive

SLC7A11 AP001160.4 0.425913588 2.13E-22 postive

NCKAP1 AP001347.1 0.401554852 7.19E-20 postive

NDUFA11 AC069281.2 0.525083642 4.37E-35 postive

NUBPL AP000866.1 0.402237687 6.15E-20 postive

NCKAP1 AP000866.1 0.441025471 4.50E-24 postive

NCKAP1 OVCH1-AS1 0.477791725 1.62E-28 postive

NDUFS1 AC004943.2 0.404170772 3.94E-20 postive

NUBPL AC004943.2 0.484670049 2.07E-29 postive

NCKAP1 AC004943.2 0.510898385 5.24E-33 postive

NCKAP1 LINC00641 0.40142584 7.41E-20 postive

SLC7A11 LINC00641 0.413793844 4.09E-21 postive

NCKAP1 AC084824.4 0.54017087 2.08E-37 postive

NUBPL ACVR2B-AS1 0.40245432 5.85E-20 postive

NCKAP1 AC103703.1 0.463720933 9.41E-27 postive

NDUFS1 SUCLG2-AS1 0.437971982 9.96E-24 postive

NUBPL SUCLG2-AS1 0.510975006 5.11E-33 postive

NCKAP1 SUCLG2-AS1 0.631958895 1.96E-54 postive

NCKAP1 LYRM4-AS1 0.403756879 4.33E-20 postive

NCKAP1 FOCAD-AS1 0.417850143 1.54E-21 postive

NDUFA11 SNHG7 0.474511162 4.24E-28 postive

NCKAP1 ITCH-IT1 0.413032795 4.91E-21 postive

NCKAP1 AL008718.3 0.42670297 1.75E-22 postive

OXSM AP001372.2 0.415372618 2.80E-21 postive

NUBPL AP001372.2 0.407814867 1.69E-20 postive

NCKAP1 AP001372.2 0.461697545 1.66E-26 postive

NCKAP1 AC007285.1 0.477921921 1.56E-28 postive

SLC7A11 AL451123.1 0.405159662 3.13E-20 postive

NCKAP1 RB1-DT 0.481966939 4.67E-29 postive

NCKAP1 AC145146.1 0.410655331 8.63E-21 postive

NCKAP1 AL139081.1 0.402842916 5.35E-20 postive

NCKAP1 AC026412.3 0.402408774 5.91E-20 postive

LRPPRC THAP9-AS1 0.414883285 3.15E-21 postive

NDUFA11 THAP9-AS1 -0.40787663 1.66E-20 negative

NCKAP1 THAP9-AS1 0.45721025 5.79E-26 postive

NCKAP1 AL513534.2 0.461363138 1.83E-26 postive

NCKAP1 ZNF8-ERVK3-1 0.426963519 1.64E-22 postive

NCKAP1 AL160396.2 0.466660114 4.09E-27 postive

NCKAP1 PPP3CB-AS1 0.499276392 2.25E-31 postive

NCKAP1 AP002026.1 0.416647305 2.06E-21 postive

NCKAP1 RPL37A-DT 0.412187001 6.01E-21 postive

NUBPL AL031123.5 0.412359028 5.76E-21 postive

NCKAP1 AC012254.3 0.424143741 3.30E-22 postive

NDUFA11 AC012615.1 0.445186101 1.50E-24 postive

SLC7A11 AC069549.1 0.401975867 6.53E-20 postive

NUBPL AC036214.2 0.461771448 1.63E-26 postive

NCKAP1 AC036214.2 0.46832262 2.54E-27 postive

NCKAP1 AC008180.3 0.428480364 1.12E-22 postive

NCKAP1 AC013652.1 0.502498555 8.04E-32 postive

NCKAP1 AC103923.1 0.417767631 1.57E-21 postive

NCKAP1 Z97989.1 0.483686618 2.79E-29 postive

NCKAP1 ACBD3-AS1 0.440334905 5.38E-24 postive

NDUFS1 EBLN3P 0.454777602 1.13E-25 postive

NDUFA11 EBLN3P -0.43609268 1.62E-23 negative

NUBPL EBLN3P 0.419898341 9.37E-22 postive

NCKAP1 EBLN3P 0.640533255 2.50E-56 postive

NUBPL AL512353.1 0.408680386 1.38E-20 postive

NCKAP1 AL512353.1 0.42364772 3.73E-22 postive

NCKAP1 AC010245.2 0.471534379 1.01E-27 postive

NCKAP1 AC107071.1 0.432330964 4.24E-23 postive

NCKAP1 MCM8-AS1 0.424968397 2.69E-22 postive

NCKAP1 AC093157.2 0.49886807 2.56E-31 postive

NCKAP1 AL033381.3 0.432817245 3.74E-23 postive

NCKAP1 AL138921.1 0.448904508 5.56E-25 postive

NCKAP1 JPX 0.435246103 2.01E-23 postive

SLC7A11 AC108463.2 0.415851797 2.50E-21 postive

NCKAP1 Z83843.1 0.456858978 6.38E-26 postive

NCKAP1 AC021237.1 0.421284998 6.68E-22 postive

SLC3A2 MAFG-DT 0.45899375 3.53E-26 postive

NCKAP1 LINC00863 0.520163506 2.36E-34 postive

NCKAP1 AL354993.2 0.471921792 9.02E-28 postive

NCKAP1 AC119403.1 0.447903003 7.28E-25 postive

NCKAP1 AC097500.1 0.479581143 9.52E-29 postive

RPN1 INE1 -0.406076916 2.53E-20 negative

NCKAP1 AC008269.1 0.426888345 1.67E-22 postive

NCKAP1 AC010491.2 0.421396435 6.50E-22 postive

NCKAP1 AC139795.2 0.433966915 2.79E-23 postive

NCKAP1 AL353796.1 0.466411679 4.39E-27 postive

NUBPL AL359232.1 0.481637273 5.15E-29 postive

NCKAP1 Z99127.3 0.46511436 6.34E-27 postive

NCKAP1 AC026254.2 0.444711978 1.70E-24 postive

NCKAP1 NCK1-DT 0.506593415 2.14E-32 postive

NCKAP1 AL049646.1 0.423314711 4.05E-22 postive

NCKAP1 SLC5A4-AS1 0.417562079 1.65E-21 postive

NCKAP1 AL354833.1 0.43368474 3.00E-23 postive

NCKAP1 AC117383.1 0.42880489 1.03E-22 postive

NUBPL PLBD1-AS1 0.610570881 5.79E-50 postive

NCKAP1 PLBD1-AS1 0.415097051 3.00E-21 postive

NCKAP1 AC009754.1 0.409339248 1.18E-20 postive

NCKAP1 FSIP2-AS1 0.436954549 1.30E-23 postive

NCKAP1 AL354989.1 0.415326672 2.84E-21 postive

NDUFA11 AL117332.1 0.445986141 1.21E-24 postive

NCKAP1 AL356124.1 0.418527463 1.31E-21 postive

NCKAP1 AC078795.1 0.43350107 3.14E-23 postive

NCKAP1 AC068790.5 0.416927599 1.93E-21 postive

NUBPL AC008966.1 0.463815634 9.16E-27 postive

NCKAP1 AP003110.1 0.412778229 5.22E-21 postive

NCKAP1 NUTM2B-AS1 0.481496272 5.38E-29 postive

NCKAP1 DGUOK-AS1 0.416380744 2.20E-21 postive

NCKAP1 AL031775.1 0.468887257 2.16E-27 postive

NDUFA11 AC074212.1 0.495308771 7.84E-31 postive

NDUFA11 AC010642.2 0.666316904 2.12E-62 postive

NDUFA11 U47924.2 0.459958791 2.70E-26 postive

NCKAP1 AC096992.2 0.501148857 1.24E-31 postive

NCKAP1 AC008543.1 0.419782418 9.64E-22 postive

NCKAP1 MBNL1-AS1 0.412280857 5.87E-21 postive

NCKAP1 AC114980.1 0.429896006 7.85E-23 postive

NCKAP1 AL133243.2 0.487612108 8.47E-30 postive

NCKAP1 AL158825.2 0.441714355 3.75E-24 postive

NCKAP1 AC092645.2 0.466086752 4.81E-27 postive

NCKAP1 PTPRG-AS1 0.436926976 1.30E-23 postive

OXSM AC012640.2 0.408387012 1.47E-20 postive

NUBPL STX18-AS1 0.419541475 1.02E-21 postive

NCKAP1 STX18-AS1 0.48323378 3.19E-29 postive

NCKAP1 AL157938.3 0.423491204 3.88E-22 postive

NCKAP1 RSF1-IT1 0.445305779 1.45E-24 postive

NCKAP1 AL035071.2 0.480135117 8.07E-29 postive

NCKAP1 AC025171.2 0.451039462 3.13E-25 postive

NCKAP1 AC008114.1 0.414218184 3.70E-21 postive

NCKAP1 AC007038.1 0.52080452 1.90E-34 postive

NCKAP1 AC016737.1 0.406956695 2.06E-20 postive

NCKAP1 MIS18A-AS1 0.455499779 9.28E-26 postive

NCKAP1 SEPSECS-AS1 0.523397072 7.81E-35 postive

NCKAP1 AC009120.2 0.408086126 1.58E-20 postive

NCKAP1 AC015987.1 0.423433061 3.94E-22 postive

SLC7A11 AC015987.1 0.400443839 9.27E-20 postive

NUBPL UBR5-AS1 0.484396996 2.25E-29 postive

NCKAP1 UBR5-AS1 0.421665719 6.08E-22 postive

NCKAP1 AC025031.4 0.455776834 8.60E-26 postive

NCKAP1 AF117829.1 0.497456685 3.99E-31 postive

NCKAP1 PPP1R12A-AS1 0.490008667 4.06E-30 postive

NCKAP1 AL034422.1 0.400683197 8.78E-20 postive

NCKAP1 SNHG31 0.454432349 1.24E-25 postive

NCKAP1 AC100791.3 0.409351153 1.17E-20 postive

NCKAP1 AC092944.1 0.465655856 5.44E-27 postive

NCKAP1 AC093510.2 0.44347563 2.36E-24 postive

NCKAP1 AC092910.3 0.430579949 6.60E-23 postive

NCKAP1 AL122035.1 0.431900657 4.73E-23 postive

NCKAP1 MRPS30-DT 0.466787062 3.94E-27 postive

SLC7A11 MRPS30-DT 0.542113692 1.02E-37 postive

NCKAP1 AC024940.5 0.485176612 1.78E-29 postive

NCKAP1 AC005261.1 0.525380737 3.94E-35 postive

SLC7A11 AC005261.1 0.418066878 1.46E-21 postive

NCKAP1 AC016831.1 0.4018249 6.76E-20 postive

NDUFA11 WAC-AS1 -0.480170475 7.99E-29 negative

NCKAP1 AC073529.1 0.496672608 5.11E-31 postive

NCKAP1 AL355073.2 0.466360561 4.45E-27 postive

NCKAP1 AC131568.1 0.415701576 2.59E-21 postive

NCKAP1 AC023509.6 0.405193621 3.11E-20 postive

NCKAP1 AC005332.2 0.453408926 1.64E-25 postive

NCKAP1 AC004918.3 0.5618241 5.94E-41 postive

NDUFA11 AC009005.1 0.494707231 9.47E-31 postive

NCKAP1 AC005062.1 0.459459133 3.11E-26 postive

NCKAP1 BDNF-AS 0.49137753 2.66E-30 postive

NCKAP1 AL512343.2 0.445176998 1.51E-24 postive

NCKAP1 OSGEPL1-AS1 0.413678786 4.21E-21 postive

NCKAP1 SMC5-AS1 0.432431731 4.13E-23 postive

SLC7A11 AL157392.2 0.405708655 2.76E-20 postive

NCKAP1 FAM111A-DT 0.455494293 9.29E-26 postive

NDUFS1 AC016394.2 0.42975301 8.14E-23 postive

NCKAP1 AC016394.2 0.439635117 6.46E-24 postive

NCKAP1 CARNMT1-AS1 0.403726423 4.36E-20 postive

NCKAP1 AC016831.4 0.409900381 1.03E-20 postive

NDUFS1 COX10-AS1 0.401076878 8.02E-20 postive

NCKAP1 COX10-AS1 0.578975318 5.96E-44 postive

NCKAP1 PDC-AS1 0.457203206 5.80E-26 postive

NCKAP1 AC137770.1 0.449472279 4.78E-25 postive

NCKAP1 AC004908.3 0.479655298 9.31E-29 postive

NCKAP1 AC005899.7 0.401164678 7.86E-20 postive

NCKAP1 AL157786.1 0.448320944 6.51E-25 postive

NCKAP1 AC027237.3 0.450698485 3.43E-25 postive

NCKAP1 AC009262.1 0.402650574 5.59E-20 postive

NDUFA11 SPINT1-AS1 0.468463821 2.44E-27 postive

NCKAP1 AC008937.3 0.480027865 8.33E-29 postive

NCKAP1 PLS1-AS1 0.447210528 8.76E-25 postive

NCKAP1 LINC00449 0.442861958 2.78E-24 postive

NDUFA11 AC009065.2 0.723243877 3.13E-78 postive

NCKAP1 AC009065.2 -0.417474333 1.69E-21 negative

NCKAP1 AL138820.1 0.486187356 1.31E-29 postive

NCKAP1 AL604028.1 0.477993461 1.52E-28 postive

NCKAP1 TTC28-AS1 0.465373357 5.90E-27 postive

NCKAP1 AC011978.2 0.531735653 4.27E-36 postive

NCKAP1 AP005131.1 0.436248159 1.55E-23 postive

NCKAP1 PCBP1-AS1 0.426068182 2.05E-22 postive

NCKAP1 AOAH-IT1 0.48677512 1.09E-29 postive

NCKAP1 AC131159.1 0.454988133 1.07E-25 postive

NCKAP1 AL117339.3 0.42776663 1.34E-22 postive

SLC7A11 GK-IT1 0.407735089 1.72E-20 postive

SLC7A11 AC112496.1 0.425069359 2.63E-22 postive

NCKAP1 AC010168.1 0.401531071 7.23E-20 postive

NCKAP1 AC016597.1 0.407802634 1.69E-20 postive

NCKAP1 GAS5-AS1 0.529267598 1.02E-35 postive

NCKAP1 AC022211.1 0.400495178 9.17E-20 postive

SLC7A11 AP003555.1 0.478587394 1.28E-28 postive

NCKAP1 AC010976.1 0.410710982 8.52E-21 postive

NCKAP1 DLEU2 0.468932013 2.14E-27 postive

NCKAP1 AL162595.1 0.45244685 2.14E-25 postive

NCKAP1 AC103739.3 0.405539806 2.87E-20 postive

NCKAP1 AC009318.2 0.473257087 6.12E-28 postive

NCKAP1 AC020704.1 0.410227276 9.55E-21 postive

NCKAP1 AL607028.1 0.429364616 8.97E-23 postive

NCKAP1 AP003086.1 0.425454018 2.39E-22 postive

NCKAP1 AF230666.2 0.42889966 1.01E-22 postive

SLC7A11 AF230666.2 0.416307337 2.24E-21 postive

NCKAP1 AC114763.1 0.434081726 2.71E-23 postive

NCKAP1 AC025031.3 0.443219771 2.53E-24 postive

RPN1 Z83745.1 -0.434286927 2.57E-23 negative

NCKAP1 AC004067.1 0.475321878 3.35E-28 postive

NDUFA11 AL590714.1 0.500354875 1.59E-31 postive

NCKAP1 DLEU1 0.509535233 8.20E-33 postive

NCKAP1 AC025165.4 0.4158863 2.48E-21 postive

NCKAP1 AL158212.3 0.458124335 4.50E-26 postive

NCKAP1 CHN2-AS1 0.42278027 4.63E-22 postive

NCKAP1 EGOT 0.412702014 5.31E-21 postive

NCKAP1 AL512506.1 0.482227482 4.32E-29 postive

NCKAP1 AL390728.6 0.452222189 2.27E-25 postive

NCKAP1 TTN-AS1 0.424768261 2.83E-22 postive

NCKAP1 AL138963.1 0.461806108 1.61E-26 postive

NCKAP1 LINC01068 0.401485724 7.31E-20 postive

SLC7A11 LUCAT1 0.40046781 9.22E-20 postive

NCKAP1 CDC42-IT1 0.463685281 9.50E-27 postive
