# Supplementary material for: Disulfidptosis-related long non-coding RNA signature predicts the prognosis, tumor microenvironment, immunotherapy, and antitumor drug options in colon adenocarcinoma
Source: Apoptosis. 2024 Aug 8;29(11-12):2074–90. doi: 10.1007/s10495-024-02011-x (PMC11550253; doi:10.1007/s10495-024-02011-x)
Supplement: Supplementary file 4 — Supplementary Material 4 [file 10495_2024_2011_MOESM4_ESM.docx]

id futime fustat AC006460.2 AL031118.1 AC010198.2 AC124067.4 MKLN1-AS AL683813.1 AC007128.2 AC126696.3 AC112220.2 FRMD6-AS1 CASC9 ZEB1-AS1 FAM160A1-DT AC018695.4 AC007128.1 ATP2A1-AS1 AC010618.3 AC107308.1 AP001160.4 AC069281.2 SNHG7 AL138921.1 AC074212.1 AC025171.2 SMC5-AS1 AL604028.1 AP003555.1

TCGA-CM-6165 1.336986301 0 0.195599575 0.614945367 0.052276765 6.464165065 1.955610389 1.165558254 0.965322548 0.095586908 2.778860376 0.257855464 0 1.763581543 2.92229301 1.471395589 1.824849302 1.319964694 0.780562076 0 0 0.21238257 3.596470067 0.562083643 0.264716569 1.657822903 0.904426293 4.054162556 0.715893371

TCGA-AA-3956 2.835616438 0 0.101246657 0.596935142 0.097745622 3.77113552 1.550260419 1.047119549 0.695993813 0.53216732 1.895302621 0 3.96703946 1.636172167 2.386948865 1.547647242 1.086647913 0.981268152 0.855830275 0 0.580724376 0.472071799 3.750992315 0.373286274 0.101381142 0.570462931 0.900721928 2.810443104 1.080862286

TCGA-AA-3527 0.002739726 0 0.052694111 0.372840689 0.032806145 3.634035597 0.285224645 0.365916454 0.397364885 0.424599662 0.94927345 0 1.377512488 1.011137783 0.857025515 0.27596058 0 1.167036722 0.738983955 0 0 2.835439053 5.738135506 0 0.69099808 0.456280482 0.164657575 1.671565126 0.279649579

TCGA-CA-6716 1.016438356 0 0.388905884 0.94373383 0.103262627 5.553004087 1.995556878 2.12383171 0 0 2.707568963 0.188907267 3.66120233 2.309758271 2.782471472 1.237502534 0.059493725 0.953227984 1.429374937 0.332278283 0.267955212 0.098823768 4.793547389 0.241840184 0.704606413 1.7822408 0.405556763 3.742674569 0.851039386

TCGA-D5-6530 1.701369863 0 0.294076649 0.231801023 0.011209361 3.571409844 1.646024373 1.11789448 0.567545448 0.157819839 1.961734443 0.446891405 2.054501216 0.80182086 1.611408481 0.805375011 1.499118777 1.177726483 0.54606718 0.31556623 1.108223341 0.202260984 4.596905334 0.156784908 0.294429602 0.977829417 0.385596696 3.59391544 1.059147581

TCGA-G4-6323 1.147945205 0 0.182565182 0.593975179 0.05630606 5.60329994 1.785927131 0.559540759 0.496513481 0.373620373 2.842435834 0.182819402 3.642355006 2.153935011 1.633384709 0.819422945 0.921131611 0.718526026 0.993275822 0.807437359 0.479334051 0.095451881 3.449640605 0.160145725 0.366811773 1.127237062 0.729444007 3.563658471 1.287472295

TCGA-A6-5661 2.794520548 0 0.629146758 0.32751492 0.740539049 3.771737711 1.223546116 0.867342995 0 0 1.775977615 0.225151512 2.372938172 0.940655398 1.849619026 1.371530984 0 0.175237151 1.047154457 0.322158908 0.932439187 0.222804561 3.174981592 0.435575259 0.120219212 0.967389976 1.383441646 3.032691557 2.74898712

TCGA-AY-4070 1.35890411 1 0.269870983 0.170566057 0.064469017 6.484215485 1.100708589 1.156072961 0.74381653 0.570851483 1.696082866 0.529671048 3.592146039 2.862748926 0.757450016 1.283921772 1.222804561 1.825337895 0.982582947 0.496308945 1.843300512 1.378899989 5.688283884 1.042994464 1.464040872 0.667210912 1.092207438 2.575360742 1.586020089

TCGA-AA-3530 1.589041096 0 0.474773476 0.549422746 0.22527493 4.927247 1.865008185 1.816108144 0.413918977 0.442120759 2.790876275 0 4.135165448 1.532067552 1.82806284 1.217416858 1.891535842 0.834387615 1.701992554 0 0.845429833 0.465086379 4.084242889 0.560519322 1.300592341 0.664300785 0.251082803 3.547030237 0.923339372

TCGA-AA-3655 5.084931507 0 0.424062125 0.260146127 0.22897257 5.443759134 2.533314157 1.86904226 0 0.258820397 1.912764816 0.458591988 3.724824869 1.212693838 2.865938604 1.063088712 0.063502942 2.323543009 1.030618586 0.352871496 0.284869433 0.155360663 4.44422974 0.406318971 0.502839758 1.170822399 1.396104674 3.206111804 1.155943478

TCGA-A6-5656 2.742465753 0 1.459554024 1.267176203 0.668754533 4.902969155 2.775542246 2.801358729 1.85913469 0.390704367 3.460288238 1.393507728 4.21543528 2.248575315 3.475487017 3.001159701 3.153708072 0.533330771 2.472816998 2.37952855 3.372394965 0.116187293 3.909558716 1.726104602 0.222845769 2.201853385 2.308263784 4.532262925 1.043064479

TCGA-A6-2676 3.575342466 1 0.566084491 0.07751647 1.487023213 2.948937188 1.105879183 0.72141641 0.776188231 0 1.200692666 0.449957484 1.600269754 1.937946959 0.54181217 0.774418104 0.435308552 3.469365054 0.620023298 0 1.648833548 0.638027485 4.893604905 0.466548817 0.702746186 1.335025838 0.493237419 2.628656962 0.566181934

TCGA-CM-5862 0.419178082 1 0.200002066 0.509341141 0.02034218 4.650454392 1.969859619 1.327687364 1.899755686 0.357045213 1.400210007 0.163885117 4.508682179 2.115266347 1.905235356 0.175939705 3.238664592 0.820812077 0.745280365 0 0.553655402 0.085288675 5.273937298 0.097206246 0.141563582 0.559932264 0.639695233 3.359619558 0.178364082

TCGA-A6-6648 2.098630137 0 0.254714968 0.433920884 0.04949137 6.860014259 2.01210379 0.686253987 0.825215762 0.28936234 2.950487078 0.144960005 3.317246174 1.151014509 3.236599764 1.820076821 2.252294541 0.788769222 1.005831115 0.701593413 1.159500022 0.04558276 3.887447285 0.451646328 0.240375394 0.890485605 0.835843249 3.098655361 1.337482754

TCGA-CM-6679 0.838356164 0 0.337882322 1.247380704 0.092342769 5.551534474 2.605043866 1.867698821 0.690461795 0.135009441 3.114999939 0.22428729 2.85630849 2.168256923 2.898479256 1.667210912 1.975630186 1.192699178 1.368935932 0.698307394 0.862114111 0.295252823 3.715212486 0.619178216 0.634686193 1.115033243 0.553950241 3.568555078 0.941106311

TCGA-AA-A02K 1.167123288 1 0.317072478 0.10057404 0.146915848 4.929909324 1.986920567 1.690104161 0.949871056 0.223546116 1.511468039 0.252657864 3.35356325 2.524113838 1.316087802 1.34953513 1.844224943 1.453280513 1.27596058 0.915329676 0.983604737 0.434561512 4.543007066 0.998917573 0.848077054 0.759496755 0.390557636 2.802379359 0.632175135

TCGA-A6-3810 3.043835616 0 1.802110479 1.422789156 0.537146944 4.775654612 2.174949644 2.657159789 1.335940529 0.146698663 2.692947879 1.206184756 4.172324824 2.978842378 2.336578563 3.042013896 3.41412651 0.483226758 2.6359014 2.49931441 2.824211166 0.207643013 3.913374543 1.873695101 0.339251427 2.358161796 1.674385321 3.709228132 1.509037042

TCGA-G4-6306 3.723287671 0 0.326882449 0.098689044 0.070114503 5.057584571 2.424814621 0.926606741 0.155878733 0.409798228 2.273963399 0.129348967 4.185787274 0.961845549 0.663663412 1.146655222 1.051093629 1.883659913 0.632733526 0.430178204 1.401739675 0.13093087 4.741990037 0.860446393 0.164271398 0.545177635 0.521352314 3.92241671 0.69233792

TCGA-AA-3693 0.002739726 0 0.295370388 0.800578978 0.015782997 6.160855665 1.949983079 0.787683201 0.650488859 0.317304068 3.30487605 0 3.61407374 1.768078435 2.07282611 1.042924445 1.33393889 1.734395309 1.183200647 0 0.863700624 0.161694235 4.590374466 0.112900262 0.567253375 0.525367331 1.563353406 4.409166877 0

TCGA-AA-3712 0.002739726 0 0.23511432 0.838427422 0.449429314 4.718624658 2.0651242 2.137536312 1.65315176 0.652325868 1.543149397 0.334797075 2.653105889 1.451066011 2.504772916 1.821751026 2.591487938 0.999061943 0.896930552 0.569102173 0.652142272 0.300416628 5.220213854 0.540126488 0.189033824 0.701238528 0.240985904 2.639949859 0.280243696

TCGA-CM-6170 1.252054795 0 0.294664856 0.364684482 0.104202449 5.230802474 3.272754805 1.82130204 1.696795088 0.289834465 3.261997096 0 4.078362192 1.619366055 1.836489726 1.286171451 3.124212318 0.979440058 1.129810535 0.556306784 0 0.195095577 4.543798991 0.151209393 0.407950917 0.969970101 0.372729271 3.655866985 0.503043371

TCGA-DM-A1HA 7.123287671 0 0.770194755 0.448795255 1.941331714 2.21744788 1.47253976 0.655809754 1.078336571 0.556306784 3.079617047 0.432211149 4.679846005 2.705469276 1.87848994 1.090921161 1.647728951 0.598079306 1.580434959 0.84478735 2.251900838 0.316145742 5.889169298 0.247198389 0.569296646 1.432318067 1.334053344 4.131474248 0.52717045

TCGA-AA-3862 2.504109589 0 0.190425227 0.320311371 0.054917919 4.258850541 2.422017657 0.572307624 1.265256848 0.194465332 1.579904211 0 3.636566622 1.439729507 1.344374033 0.616545903 1.756639046 1.364740503 0.682033871 0 0.972104429 0.058939855 4.961479792 0 0.436375082 0.682573297 0.618520588 2.773173501 0.345396375

TCGA-F4-6460 2.663013699 1 0.455228571 0.733441117 0.023752136 3.759486103 2.76037 2.243973672 0.956428433 1.234807853 1.991934472 0.43445476 3.862064505 1.94106874 1.858100386 1.941782416 2.465347637 2.81666114 1.093560176 0.60445093 1.284040264 0.583326519 4.686859055 0.632082049 0.928806145 1.443500567 0.960215134 3.26964661 0.656908183

TCGA-G4-6302 0.002739726 0 0.314290484 0.815739362 0.264236151 4.073426094 1.63254742 1.281846592 0.130008304 0 1.67644796 0.431783399 2.58004898 2.159855195 0.785843455 0.586500555 0.804136185 0.917775441 0.700883556 0 0.29419431 0.082566351 4.015702726 0.094100916 0.137110008 1.238603454 0.443288375 3.315899478 0.710437277

TCGA-AA-3511 0.580821918 0 0.130140135 0.321235435 0.071762669 5.69510577 1.799501814 1.33433944 0.086647913 0.123136427 2.011746085 0.071625394 1.047677975 1.354170026 2.263244784 1.490724147 0.280006078 1.297895714 1.133563526 0.24902051 0.374955996 0.174789893 3.215367972 0.288181352 0.204891925 1.206080538 0.411860074 3.133251149 0.322505058

TCGA-CM-6167 1.249315068 0 0.39988201 0.572501665 0.185232254 4.247183195 2.482357838 1.850919409 0.39429462 0.104873377 2.611644543 0.641268561 3.097459103 2.5958378 2.607129062 1.340277405 1.465086379 1.212133508 1.237441347 0 1.00345832 0.204891925 4.235313489 0.292310588 0.331590576 2.038436182 0.94027953 3.473734968 0.190678066

TCGA-AA-3553 2 0 0.225891862 0.29172142 0.14404637 4.974992699 1.862748926 0.568226722 0.519743173 0.604261139 2.290837217 0.161952159 2.599936639 1.11516645 1.68683665 0.469260847 1.515106871 2.063571969 0.937419727 0.526068812 0.429428502 0.164013889 3.767421745 0.270947487 0.641823444 0.683292219 0.150949541 3.42548616 0.257010618

TCGA-CK-6751 1.419178082 0 0.220701417 0.35918379 0.04558276 4.80604044 1.522407338 0.891885751 0.114900024 0.308710351 1.279114664 0.095046725 2.958657108 1.438931542 1.642147027 0.412185359 0.830823203 0.833173463 0.677891557 0 0.482951513 0.186500558 4.504080075 0.235972083 0.302991616 0.725173999 0.649937299 3.020022082 0.740798068

TCGA-DM-A1D6 1.561643836 1 0.467696836 1.262072288 0.837135914 4.508041938 1.891769124 1.607531537 1.194654434 0.354113592 2.532217202 0.770786769 3.216827326 2.505535292 2.175620405 1.812826773 2.28258807 1.190678066 1.131852846 0.47736648 0.867263911 0.259061529 5.104043058 0.515813357 0.812949959 2.081816811 0.889084099 2.487177608 2.553040961

TCGA-AA-A00N 0.334246575 1 0.396049858 0.586020089 0.273575566 3.903905941 1.612069358 0.818196129 0.238297728 0 1.207330653 0.28853575 2.619553869 0.98550043 1.504976256 1.541118304 0.697329024 1.090040415 0.900103608 0 2.005292186 0.496922466 4.785173874 0.82374936 0.815001516 0.809496764 0.496615738 3.02731332 1.295017665

TCGA-AA-A00U 1.419178082 0 0.184343778 0.599127326 0.089904868 4.612859653 1.236400773 1.466914195 0.997473072 0.309176187 1.909734709 0 2.75947545 0.989139007 0.887447285 1.073134705 1.757407345 2.695949285 1.105275783 0.590146877 1.374622206 0.312665174 4.76002396 0.439357178 0.996171784 0.738205779 0.172103428 2.665028868 0.741747408

TCGA-A6-6650 1.717808219 0 0.648864219 0.883203717 0.10710755 5.385066291 2.289716448 1.992297122 0.897859974 0.394221439 3.31750668 0.259985497 2.129096141 1.260387037 3.130046756 3.029594233 1.470857862 0.77677779 1.70289391 3.060070448 2.411281607 0.152854033 4.206919277 1.026516803 0.083338198 1.699818116 1.820689561 3.93947735 1.806282807

TCGA-G4-6625 7.649315068 0 0.185105363 0.445726703 0.021621858 5.178806251 1.567983446 1.433653871 0.698485208 0.224163787 2.229157203 0.468739698 1.176195095 1.546412967 2.53126916 1.351967481 0.823504815 1.785090155 1.08841301 0.559540759 0.457751869 0.387252239 3.845028315 0.152248331 0.929866728 0.994000358 0.617674625 3.477236941 0.14404637

TCGA-CM-5348 1.915068493 0 0.386700602 0.691980751 0.075737982 5.059900339 2.728225294 1.770067863 1.688851744 0.5431989 2.84309947 0.257976116 3.501375449 2.817398139 2.651683181 1.309409049 3.177774313 1.086240276 1.212071235 0.312316654 0.817050158 1.256225675 5.085314172 0.540027269 0.615133758 1.893711677 0.955312803 3.488000771 1.74734443

TCGA-A6-2679 3.742465753 0 0 0 0 3.179558821 0.230694897 0.136585153 0.726133673 0 0.613343053 0 0.333767192 1.665756583 0 0 0 3.20518917 0 0 0 1.397200573 5.032625444 0 2.118326499 0.286053134 0 0.585923977 0

TCGA-CA-5255 1.030136986 0 0.123003954 0.160533009 1.223113589 5.125374206 1.876801774 0.889473543 0 0.285106251 2.233949398 0.044324169 4.020057652 1.419484958 0.979074161 1.077311371 0.053111336 0.674822184 0.67346597 0 0.628633634 0.19181529 4.829063067 0.076148597 0.279768422 0.505789328 0.398788148 3.192131026 1.221134668

TCGA-CA-5256 1.038356164 0 0.156655489 0.230571942 0.028993412 5.796164237 1.534609483 0.421586873 0 0.138290234 2.436854764 0 5.026299897 1.133366243 1.653335227 0 0.697595918 2.242785403 0.418999465 0 0.416947805 0.081612323 5.259264953 0.378067649 0.529271244 0.159758338 0.713343895 3.495196272 0

TCGA-AD-5900 1.01369863 0 0.460061021 0.232906301 0.424492171 4.482919255 1.343805752 0.777956188 0 0 1.806654014 0.431248532 0 1.420078116 1.290424404 0.322389683 0.065710164 1.393910378 0.540225701 0.653793793 0.293841299 0.055473336 5.337211556 0.265316867 0.136978812 0.787599627 0.238297728 3.159516168 1.100035721

TCGA-AZ-6599 0.564383562 1 0.732225777 0.281075049 0.041803688 4.524226697 1.935082523 1.424922088 1.029205495 0.321581807 2.855012957 0.09936254 4.9165483 2.748718834 1.78303751 1.822567007 1.888110029 1.154842407 1.285698126 0.841490128 1.028569152 0.10057404 4.104093394 0.580917289 1.578455722 2.21704455 1.31533436 3.437320968 3.910991633

TCGA-G4-6586 2.983561644 0 0.37784561 0.065710164 0.745366427 4.199750857 2.105845667 1.375901314 0 0.21673422 1.942270517 0.548831154 0.693765712 2.04176865 1.343123519 1.924175915 0 1.341245714 1.305153395 0.543000877 1.956391259 0.375067242 4.041689812 0.568421312 0.612682759 1.230633421 0.544188607 3.85371626 0.773236812

TCGA-AA-3518 0.084931507 0 0.37717929 0.480161699 0.075874867 3.350129454 1.284158746 0.826436626 0.2131295 0.156526059 1.759581973 1.123003954 0.979366886 1.924403979 1.784420225 1.5387368 0.899871669 1.80822028 1.039840265 0.570074274 1.101381142 0.448478121 5.430010894 0.295840551 1.594405228 0.911346444 0.313710226 3.138454078 1.194717463

TCGA-NH-A6GA 0.82739726 1 1.031677496 1.549964825 0.60757888 5.031434898 1.842295023 1.962030707 2.289067184 0.775682702 1.711715149 0.195599575 4.442107486 2.722619029 2.262373019 2.602694412 3.555987894 1.221938932 1.821791836 0.516216908 0.885496273 0.77070221 5.877592121 0.813524689 1.150689643 2.49367295 0.374622206 2.499654578 1.948787713

TCGA-F4-6461 0.926027397 1 1.220701417 0.303926836 0.023326332 1.692873507 1.219091058 1.310107409 0.741919948 0.084744621 2.311851831 0.786847244 3.85692595 1.915214929 1.750863723 1.420132027 1.787181685 0.481505617 1.212507085 0.328606717 1.143589335 0.232537969 4.691511832 0.443924858 0.894293934 1.435415241 0.5663768 3.059787883 1.093492569

TCGA-AA-A02Y 3.331506849 0 0.303459302 0.510354341 0.035483152 5.061538608 1.603311811 0.465608848 0.155878733 1.076011739 1.716639778 0 0.064055064 0.768671318 1.210077099 0.105409894 0.769687121 1.385817545 0.331017236 0.60473557 0 0.066950244 4.655128537 0 0.111565545 0.457226546 0.231432408 2.580193734 0.106884289

TCGA-CA-6717 1.063013699 0 0.236829335 0.557287542 0.118093889 3.142364421 1.507058837 0.61786266 0.111164889 0.157302466 2.644317778 0.299010149 2.852538248 2.020768865 2.836994585 0.399772661 0.161823203 2.216547989 1.060669995 0.314522522 0.253384236 0.115432826 5.423154578 0.228480098 0.154194325 1.133760781 1.438133135 2.953227984 0.979659552

TCGA-AA-3846 1.419178082 0 0.233274539 0.721941313 0.051302489 5.105108128 2.344317215 1.749233898 0.201131967 0.281431197 2.555349903 0 2.336026251 1.830295894 1.5431989 2.195662563 0.373954395 1.639695233 1.261951979 0.541415716 1.284099506 0.522055749 4.394946227 1.049073097 1.075258784 0.679874148 0.597983994 3.639336367 0.683112522

TCGA-CM-5861 1.252054795 0 0.412185359 0.097475959 0.592636429 1.475396222 1.634639731 0.630125852 0.154194325 0.216982489 1.946618584 0.405447844 4.341238596 2.055750964 1.107286135 0.378400642 0.690014739 1.939414661 0.748461233 0 1.234072066 0 3.237043569 0.215616477 0.261470639 0.917317175 0.379731849 3.104688903 0

TCGA-A6-6649 2.01369863 0 0.695281195 1.051302489 1.32308179 3.627291652 1.38708677 1.488155062 1.022758398 0.688314661 2.239856258 0.866393698 1.478764767 2.375762336 2.142870845 1.70456215 2.750713685 1.281193775 1.509797169 0.453543918 1.013497992 0.353774945 5.126853122 0.382501257 0.556208672 2.011316722 0.962734091 3.139027385 2.176546182

TCGA-CA-5797 1.049315068 0 0.026162654 0.341074883 0.037733627 7.113779665 1.802689543 0.583615357 0.209765266 0.744591687 2.048305947 0.174534255 4.108457548 1.323831197 3.537134516 0.604071324 0.888149004 1.156461339 0.602931904 0.308127844 0 0.112900262 3.356819915 0.223669671 0.254473108 0.805209896 0.237563718 3.48357074 0.396817103

TCGA-3L-AA1B 1.301369863 0 0.307778227 0.768925336 0.016924144 5.485951641 1.93402577 1.791522378 1.47736648 0.433173124 2.17979765 0.552475444 4.067957025 2.145775262 2.178905819 2.045163352 2.126972856 0.711230572 1.183518274 0.990809676 1.650488859 0.401412025 4.552555368 0.98928436 0.62564456 1.439889048 0.694746501 3.786282698 0.285461405

TCGA-CK-5914 1.832876712 0 0.209765266 0.595694607 0.016353684 6.19499515 2.083792032 1.778629126 0.305211776 0.358958826 2.628353671 0.403485883 0.029700234 1.51752767 2.771188354 1.933081585 1.701061053 1.237074171 0.870581724 1.283210618 0.461109415 0.135009441 5.049587207 0.693498109 0.320773477 0.912113305 0.730139952 3.409078601 1.114566923

TCGA-CM-6675 1.087671233 0 0.516216908 0.635986504 0.196355243 6.477625524 2.591487938 1.329468079 0 0 3.055438629 1.533114772 4.616487091 2.918538893 2.996298349 1.248291938 0 1.02467428 1.256527627 0.363114995 1.48769214 0.257976116 5.019141449 0.488720653 0.448372395 2.569175103 2.128227402 3.695860224 1.010923027

TCGA-AY-6386 1.484931507 0 0.121678557 0.718876685 0.11196609 5.410252901 1.909773104 0.490262049 0.585058677 0 2.151404251 0 3.423322685 1.241413107 3.315638682 0.73534887 1.651866835 0.60767356 0.461319002 0 0.677711186 0 4.00002705 0.162338958 0.19761381 0.640435837 0.498965654 2.90898579 2.012854681

TCGA-G4-6321 1.84109589 0 0.209016592 0.174789893 0.367035516 2.620774066 2.28856528 0.265196827 0.11516645 0.162983392 2.895981149 0.309176187 2.658120062 0.960659975 0.711759193 0.151209393 0.364572432 0 0.585827858 0 0.483880254 0.16491497 2.667460728 0.307428525 0.268913416 0.820485343 0.431462503 3.547351112 0.291839273

TCGA-G4-6320 2.202739726 0 0.257855464 0.940956022 0.159370847 4.94045808 2.258790252 1.688583227 0.354113592 0 1.85941303 0.465817783 3.813750413 2.650764559 1.128491379 1.076901088 2.057588903 1.114766793 1.02446153 1.269631651 1.688672738 0.300533772 5.161315327 0.482848283 1.143262793 1.195347598 0.646899949 3.560127976 1.170566057

TCGA-AA-A00K 1.504109589 0 0.254594043 0.140124224 0.233029057 5.725097634 0.957840327 0.806695254 0 0.961475162 1.736604875 0 2.589883307 1.09241043 2.127171015 1.339992484 0.573471479 2.207892852 0.760859637 0.585731733 1.126906797 0.269631651 4.90850551 0.556110553 1.598984459 0.442120759 0.32308179 3.054935279 1.352815012

TCGA-AA-A01I 2.583561644 0 0.299244658 0.214622201 0.546956178 4.954112539 1.421533016 1.799460369 0 0.376068071 1.804466643 0.117695043 3.647061181 1.090311471 1.640574657 0.632454358 1.480316831 0.858219766 0.78014209 0 0.31973353 0.030689204 3.047276628 0.289008146 0.196355243 0.668663778 0.43839932 3.232630061 0.355241844

TCGA-CK-6747 2.246575342 0 0.155619721 0.527770989 0.022474347 5.909401104 1.838185352 0.668845283 0.776103988 0.128557365 2.092714864 0.213751646 3.505979825 1.697551439 2.867402306 0.426962448 1.148413534 1.215927048 0.554343266 0 0 0.094100916 3.927574134 0.065296567 0.241962182 0.714135594 0.973280639 3.110914423 1.208329966

TCGA-AA-3952 0.167123288 1 0.412185359 0.343692069 0.073820233 4.806267338 1.033299651 0.866314561 0 0.554146767 2.057450272 0.849839173 2.896310604 2.70194821 2.649155563 1.2880632 0.668845283 4.074591064 1.324176946 0.411534715 1.356425559 0.444879056 5.599716503 0 0.849999259 0.428035163 1.05338942 3.054917919 0.196984664

TCGA-NH-A6GB 1.304109589 0 0.064606975 0.12551886 0.105275783 5.603721285 1.839919288 1.592923408 0.257493448 0.434561512 2.96449105 0.214870834 1.479489271 1.365300599 1.807354922 1.995556878 1.348005741 1.251991702 1.180402507 0.673285045 1.266516713 0.492314685 4.484879272 0.355354621 0.498863564 0.951214764 0.45691126 3.109861988 1.573956141

TCGA-AA-3562 1.665753425 0 0.325156118 0.180911663 0.019915368 5.780010819 2.022545365 0.687598239 0 0.601221086 2.3918227 0.161049225 1.782324685 1.453543918 1.505128742 0.466862004 0.58236331 2.340334382 0.83163407 1.45906434 1.024177814 0.162983392 4.596905334 0 0.729618025 0.567253375 0.410123978 3.387555549 0.472695713

TCGA-DM-A280 0.646575342 1 0.275126274 0.187641078 0.34619103 4.974666968 2.231831736 0.516519498 0.457646819 0.280837569 1.553360503 0.085968454 2.943790081 0.711671103 0.818932344 0.261229909 0.491596594 0.434988439 0.49067281 0.749834574 0.620211026 0.044184258 4.279107233 0.146264195 0.03745251 0.3222743 0.650213105 3.293650049 0.681943947

TCGA-AA-3522 3.087671233 0 0.08011186 0.967463758 0.296075575 5.272859292 2.363339312 0.577537593 0 0.302640751 3.213627239 0.263034406 2.23287561 2.145710058 2.243516761 0.281787257 1.466809811 1.338681125 0.752320428 0 0.830255324 0.225028083 5.30457675 0.430499386 0.5431989 0.794187097 0.851359274 3.27749385 2.304218969

TCGA-CM-6168 1.082191781 0 0.325040955 0.566474224 0.143262793 5.198006887 1.707789805 1.474462002 0.119289768 0.696171913 2.574367976 0.359858472 2.513465479 1.779595925 2.493391151 1.247927513 0.635893663 0.857980995 1.117030053 0.336054824 0.869239719 0.281312491 4.308957845 0.577924245 0.382501257 1.404303691 0.54606718 3.023095636 1.260206358

TCGA-AA-3526 1.589041096 0 0.124592825 0.308360875 0.037171338 5.770226476 1.162983392 0.8613202 0.469260847 0.221567789 2.877920896 0.430285273 3.544238074 0.989066326 2.302786955 0.494466814 1.478298822 2.246256057 0.502941568 1.265256848 0.63505783 0.132247798 3.556993385 0.077106244 0.282973489 0.907813625 0.768586635 2.990555569 1.026516803

TCGA-G4-6315 5.15890411 0 0.24902051 0.320773477 0 6.324941981 1.376790461 1.068326861 0.48707468 0.444879056 2.419565858 0.319039816 3.255364766 1.335940529 2.424089007 0.951811567 1.098621677 2.054779032 0.954643011 0.938549274 0.567058626 0.087734382 5.214831988 0.363899952 0.710789906 0.652692989 0.427177055 3.458893787 0.183200647

TCGA-F4-6854 0.043835616 0 0.217975141 0.255682003 0.028852006 5.397166338 1.686612577 1.32083123 0.875150958 1.035975744 2.357326786 0.156137698 1.748675904 1.140124224 3.141841475 1.462575888 1.386921282 2.132905812 1.133826527 0.276913483 0.415109632 0.176578094 4.890271574 0.136585153 0.477055566 1.009848767 0.308710351 3.201555452 0.248048998

TCGA-AA-A02F 3.331506849 0 0.622086971 0.307428525 0.123136427 5.017476563 1.290601338 1.97460273 0.820648719 0.412293771 2.267955212 0.088141597 1.370387504 1.584770129 0.477262849 1.945682608 1.095114259 1.425136999 0.908889747 0.950617715 0 0.252657864 4.421835936 0.149909668 2.749062231 1.00122577 0.433600463 3.240375394 0

TCGA-D5-6924 1.191780822 0 0.128029389 0.535754381 0.080657663 6.203866742 1.644179317 2.043379504 0.61117238 0.734915511 1.986920567 0.148478616 3.441695937 0.808096689 2.75195639 1.461161815 1.97585026 1.801779482 0.966430467 0 1.179447354 0.219958398 4.379087199 0.35918379 0.504061001 1.721985046 0.587749018 2.900006971 0.236217064

TCGA-D5-6928 0.969863014 0 0.508530069 0.555030801 0.116098552 2.536351358 1.234440006 1.44794941 0.100439479 0.602741913 1.653197629 0.271784214 0.995882449 1.703632307 1.982144815 1.917966341 0.776188231 0.84213408 1.296075575 0.286053134 0.898556647 0.937419727 4.145522581 0.497740089 0.543099892 2.242785403 0.440739631 2.60376282 3.181197985

TCGA-AZ-6606 0.978082192 1 0.179383654 0.3822799 0.01564029 4.372534269 1.9509909 1.850079296 0.537842721 0.572986653 2.346474729 0.086647913 0.110897723 2.093898163 2.095958167 1.202449067 1.624989873 0.670387157 0.948974554 0.297602299 0.624428477 0.150169706 4.898363161 0.215864939 0.369382725 1.322966462 1.064262056 2.080453011 0.686612577

TCGA-AA-A02W 3.416438356 0 0.333996118 0.498863564 0.030689204 3.952557223 2.134845206 1.175364914 1.473527177 0.512176309 2.361122651 0.085288675 4.483486902 1.462889938 1.330902541 0.988702862 1.21263159 1.629473197 1.332049084 0.536649754 0.774165052 0.317998618 3.704462553 0.612588407 0.833983011 1.271903706 0.970853654 3.543619608 0.262793937

TCGA-F4-6569 2.978082192 0 0.143393419 0.676628488 0.119422583 5.747266543 1.550408193 0.623679608 0.455439015 0.214497868 1.89569039 0.842616856 3.420900045 1.465452127 1.713563855 0.135797512 1.237563718 1.262974292 0.599032083 0 0.617392528 0.043904394 4.480937189 0 0.073546061 0.783917573 0.359633613 3.430378951 1.679694025

TCGA-AA-3529 0.002739726 1 0.402503901 0.226631832 0.086783766 4.025303483 0.934856141 0.473631077 1.068670811 0.480161699 2.343095086 0.460270761 3.787651861 0.760689347 1.265496907 1.298130404 1.236523232 0.655168618 0.683920981 0 1.211510663 0.340847077 4.973615867 0.606726475 1.067294521 0.605209844 0.763411574 3.357002972 0

TCGA-AY-5543 2.750684932 0 0.125783332 0.103128316 0.004177761 6.004953006 2.591918729 0.958063131 0.620680241 0.280837569 2.668436864 0.069152202 4.004708119 1.004393523 2.666483931 0.562083643 1.961067626 1.023113383 0.720803782 0.627886946 0.894759569 0.017779413 3.387279815 0.173767068 0.198242682 0.345169251 0.424062125 3.419255718 0.312316654

TCGA-AA-3534 2.416438356 0 0.40053793 0.358283723 0.012640181 6.401794276 1.56924803 1.217230716 1.064469017 0.177471364 2.738076042 0.201006466 3.675104568 1.277449215 1.734265229 0.569102173 2.172103428 0.652234073 0.990519264 0.635522241 0.522256668 0.203389117 6.023346294 0.831715132 0.656999681 0.84213408 1.028922711 3.137749417 0.316609184

TCGA-AA-3971 1.339726027 0 0.353774945 0.315913965 0.054223347 4.36357761 1.912304956 1.544930441 0.839878988 0.54072166 2.462052319 0.09071796 3.044376632 1.112233058 2.213440607 1.89929166 1.483415958 2.383911611 0.60767356 0 0.463413204 0.298892881 4.212903906 0.293605911 0.831228693 0.536152393 1.017280568 3.763719628 1.046560906

TCGA-CA-6719 1.191780822 0 0.220453786 0.94927345 0.201633861 3.844978117 1.913262832 1.407135175 0 0 2.64696905 0.300299474 2.944277504 2.399143744 2.124890544 1.583182079 0.188021051 1.023042393 1.110897723 0.651866835 0.926151273 0.475811237 3.824207771 0.417163907 0.179256247 1.372227783 0.322505058 3.131375468 1.258639521

TCGA-AA-3818 0.082191781 1 0.201884743 0.84317989 0.017779413 5.451392468 1.070458026 1.834144866 0 0.871370556 2.282113568 0.0739573 3.287738232 1.297778355 1.2083924 0.749405546 0.650580765 1.771801084 1.083723966 0 0.386369519 0.31022377 5.567244246 0.12631213 0.784085143 0.63978783 0.709202395 3.535306486 0.757961973

TCGA-QG-A5YV 3.564383562 0 0.356031095 0.353210356 0.005184376 5.957501413 1.719709156 1.36999684 0 0.215367972 2.246499298 0.126972856 3.426881962 1.497024694 2.867461614 1.878646879 0.052276765 1.523561956 1.102053383 0.749233898 1.171206827 0.16889872 5.225618131 0.400319323 0.506297265 1.145807862 0.822118275 3.325645455 1.327284963

TCGA-AA-3538 2.167123288 0 0.237196574 0.305795461 0.072586047 6.424305722 1.676808994 1.045093438 0.686522938 0.630498666 2.073203272 0.248291938 2.654091783 1.132774233 2.172615522 1.690640579 0.809908292 2.039805179 0.904965719 0.549521321 0 0.172231469 2.520170776 0.284158746 0.717298052 0.298423712 0.367930141 3.663173809 0.496820231

TCGA-AA-3544 1.167123288 0 0.133563526 0.072174417 0.011495639 4.002252452 2.269751322 0.322966462 0 0.874049364 1.589140262 0 1.461790463 1.507769274 0.884480703 0 0.408059648 2.63131386 0.676357687 0 1.756553654 0.141694362 6.860045308 0.437653878 0.813360504 0.443924858 0.52747075 2.716354432 0.290542363

TCGA-AM-5821 0.076712329 0 1.374455282 0.790521838 1.183772326 0.539233271 1.47316347 0.631895859 0.5387368 0.334110568 1.16774328 0.569199413 3.455228571 1.286408055 0.300650908 0.444773066 0.988920951 0.512783121 1.026516803 1.249566698 0.901880564 0 5.067866701 0.09044698 0 0.737946293 0.229710962 2.988175678 2.672312185

TCGA-D5-6535 1.260273973 0 0.241474126 0.401739675 1.008343459 5.013730228 2.646830844 1.240131118 1.302933144 0.45691126 2.502381525 0.098823768 3.360813732 1.781065898 1.867935989 1.616122405 2.183137113 0.96295614 0.953526 0.838346737 0.499680087 0.216237552 4.045993313 0.799170217 0.314174451 1.316203681 0.480265122 2.769200471 0.302055788

TCGA-F4-6704 0.128767123 0 0.217851097 0.435628594 0.043484498 1.477107389 1.147241564 0.778965477 0.977243283 0 1.941031169 1.027507957 3.5098225 2.525166845 2.494159564 1.045373071 2.436694888 1.758047282 0.992913418 0.469365054 0.381837084 0.155749233 3.748418295 0.311270591 0.501311749 2.268823611 1.020057652 2.577199187 1.830620415

TCGA-CM-6172 0.917808219 0 0.385044426 1.118359726 0.037171338 6.492422047 1.977316563 1.173575207 1.117163075 0.09044698 2.948563471 0.601696516 4.590715783 2.183359469 2.850699427 1.99613562 2.826029786 1.430713468 1.040752186 0.864810146 0 0.335826224 4.546554962 0.535853894 0.431462503 1.482332021 1.346815094 3.466914195 0.165172318

TCGA-A6-6781 1.638356164 0 0.940930973 0.521285302 0.931758688 2.826348488 1.502008043 1.935635752 1.104806298 0.329869872 3.225316067 1.038623472 2.431863612 2.362965431 1.761441309 0.977414263 1.58292526 0.56205107 2.310718197 2.367082125 3.299811229 0.350987492 3.964645068 0.898608239 0.206309807 2.270259814 1.18330653 3.582375354 2.116409119

TCGA-F4-6808 2.805479452 0 0.170309669 0.351628329 0.01235413 6.296853819 1.865483368 1.199876467 0.341530387 0.534559685 1.741488559 0 2.915157552 1.220887111 1.609045731 0.670749711 1.129942384 1.977463111 0.693587315 1.059286049 1.047957107 0.103128316 3.451422142 0.327112471 0.358733827 0.326192164 0.307661669 3.574295308 0.444030911

TCGA-AZ-6607 0.265753425 1 0.871212824 0.935987696 1.289185254 1.29390014 1.887213303 1.148022983 1.340220425 0.117429084 3.094202283 1.53037044 4.626176274 2.792209847 2.298570344 0.70194821 1.946506299 0.799584701 0.649201559 2.898208353 0.359071312 0.102456577 5.160262728 0 0.169283661 1.322562741 1.055542748 2.554269582 3.161661992

TCGA-DM-A1D7 1.109589041 1 0.438718677 0.825052902 0.091530593 6.400621606 1.518484782 1.764600936 0.216982489 0.25664839 2.380507819 0.180657108 2.065813545 1.610700062 1.804094873 1.105007525 0.592827755 2.915788573 1.173703117 1.212507085 1.680684423 0.266276823 4.686926269 0.887993096 0.878764571 0.787098082 0.749319725 3.484782623 0.595503661

TCGA-AD-6901 1.868493151 1 0.733093981 1.122672719 0.577440913 4.243882301 2.243151129 2.675996539 0.597698019 0 3.294826571 0.225028083 3.296339931 2.100372194 1.750263476 1.541465278 1.82277093 0.853197255 1.67676387 0.392207499 1.570220033 0.676538227 4.809115996 0.371168515 0.723427501 1.566425513 0.802317314 3.447248569 2.366000414

TCGA-AA-3848 0.838356164 1 0.169925001 0.260989139 0.014783753 4.045740007 0.785341299 0.601886645 0.889707158 1.426318435 1.251809968 0.232906301 0.646715661 1.331991778 1.898247056 2.737470449 0.507515586 2.59091335 1.309234406 0.72141641 1.016139703 1.837458899 5.640221795 0.20451637 1.829403087 0.550506701 0.573180603 1.396652728 1.446626783

TCGA-AA-3543 0.082191781 0 0.299361898 0.266876472 0.192699178 2.054813755 1.480213412 0.43103453 0 0 1.643209718 0.09396575 2.244460884 1.786303611 0.878607646 0.284395681 0.720803782 0.3305584 0.715541987 0 1.123599986 0.386259141 5.551928157 0 1.254352163 0.86710573 1.129217064 2.589859344 1.074094354

TCGA-F4-6806 3.452054795 0 0.274052884 0.271903706 0.196355243 5.14680183 2.457489231 1.373174891 0.416299305 0.111298453 2.707215546 0.1264443 3.059130272 1.976730221 1.834872989 1.222804561 1.215802828 0.766128675 0.770363927 0 0.617956668 0.273575566 3.215569886 0.483673919 0.518132236 0.835762419 1.014640947 3.676210981 1.037593076

TCGA-F4-6703 3.989041096 0 0.272978695 0.39176772 0.567253375 1.03470874 1.043414503 0.951960729 0.306612224 0 1.994615927 0.342213373 2.717210299 1.807519792 0.540523296 1.016710248 0.402722177 0.802565477 1.062605293 0.444030911 1.094776557 0.373843063 3.456819288 0.276794405 0.058939855 1.25664839 0.183327706 2.582146499 1.954419678

TCGA-AA-3972 4.249315068 0 0.170822399 0.962734091 0.052555009 4.085161181 1.853396894 1.515207819 0.545177635 0 2.474098531 0 2.554097638 1.924632007 1.903693892 1.695370292 1.193077822 1.767697168 1.55478529 0.949871056 0.794187097 0.43349364 4.336868918 0.727964007 1.055612157 0.864730922 0.814673463 3.342668518 0.270827916

TCGA-CM-6678 0.917808219 0 0.499884146 0.516015147 0.013212111 4.492449287 1.751420872 1.090243712 0.52747075 0.668845283 1.981487368 0.20851726 3.910070635 1.913530923 1.29390014 0.942683396 1.226631832 1.130140135 0.6332917 0.468635446 0.539630325 0.055750964 4.010305426 0.293135019 0.180529813 0.883933562 0.282617721 3.156962839 0.673918183

TCGA-AA-A00F 2.835616438 0 0.39429462 0.134483822 0.146524891 3.345765377 1.803723007 0.968717474 0.21126145 1.307311939 2.45132982 0.175811994 1.878450703 1.159887479 0.488926268 1.174789893 0.831390858 1.990083536 1.671746267 0.565402204 0.812375001 0.639602631 4.161065354 0.536649754 1.502330602 0.561399461 0.679153521 3.50902437 1.739675315

TCGA-G4-6628 6.64109589 0 1.138486844 0.378622596 0.672561119 0.457016363 0.969528122 0.948974554 0.853356969 0.301236441 2.259905175 0.550014095 2.353040936 1.987902921 1.615510465 0.974749554 1.543099892 0.325961996 1.593736208 0.316725022 0.826843352 0.223916751 3.764016997 0.080794082 0.155360663 1.525818322 0.31741985 3.460677045 0.985136067

TCGA-AA-3947 2.750684932 0 0.262673687 0.396926676 0.595790071 3.231601368 1.918806005 1.755998484 0 0 2.609565868 0 3.287501846 1.809373283 0 0 0 0.618332639 0.597698019 0 2.747817029 0.098823768 1.828428485 0.314290484 0.163498732 0.892429886 1.49487638 4.057580239 0.545474211

TCGA-AA-3986 1.589041096 0 0.10017032 0.424599662 0.033933911 5.395282204 1.218285205 0.920979227 0 0.527370657 2.088582617 0.264956718 2.174406419 1.57468283 1.935233425 0.860207991 0.737167557 1.10855791 0.99870099 0 0.912496582 0.467696836 4.899721856 0.524264315 1.226261894 0.929488038 0.598841578 2.921379201 1.109828565

TCGA-AA-3695 0.002739726 0 0.3068455 0.322620422 1.424115888 0.89219671 1.29713271 0.515409693 0 0.788351618 2.039138394 0.997545331 0 1.568469956 2.625527673 0.852877774 0.137372364 3.306145559 2.180529813 1.508479361 1.295076458 0.275364696 4.693085456 0.194591403 0.834468522 1.23572706 0.300065137 3.565012181 0

TCGA-F4-6807 3.58630137 0 0.327917257 0.743730376 0.074094354 3.705259139 1.370945413 1.297719672 0.539332545 0 2.160113447 0.533164621 4.037092248 1.899833009 1.513894947 1.290660312 1.735738782 0.775851231 0.819831652 0 0.283803271 0.294429602 3.738151723 0.175811994 0.468114072 1.466131129 0.535555333 3.039419183 1.070938821

TCGA-AA-3968 1.832876712 0 0.268673925 0.669208225 0.06502077 5.904329945 1.942420668 1.595790071 0.39758394 0.752748591 2.326479823 0.33433944 2.392647144 2.25226426 1.780688048 1.535306486 1.843340717 2.084370465 1.542505701 1.771589827 1.098554307 0.375400929 5.671075366 1.093357346 1.508276515 0.370164281 0.509543838 3.320860105 0.279887255

TCGA-DM-A28A 2.205479452 1 0.45132982 0.897705112 0.325156118 4.713013892 1.590482259 1.094641454 1.780855994 0.322389683 2.893459293 0.44105847 3.970881257 2.125915549 2.041558406 2.786512725 2.452410935 2.143328107 1.530220599 0.843501526 1.625971791 0.692159346 5.796782024 0.996171784 0.940805718 1.534061602 1.13389227 2.282499113 0.865285394

TCGA-4T-AA8H 1.054794521 0 0.437760394 0.654802126 0.220701417 4.280666855 1.665210831 1.107219168 0.370275897 0.097880434 2.193424824 0.111164889 3.430097898 0.996244108 1.320253597 1.679874148 1.290542363 0.545671894 1.321408631 0 0.766807151 0.266636643 4.394726772 0.431248532 0.426103701 0.974088728 0.375734539 2.948955871 0.609944032

TCGA-G4-6298 0.002739726 1 0.4379734 0.540622481 0.340619235 4.549114655 1.994253859 1.72643889 0.450591032 0.480988873 2.61845011 0.248899107 2.803557702 2.046630748 0.554932602 1.560617141 2.060669995 1.369717729 1.511518642 2.835257128 1.069702167 0.274887812 5.751035177 0.782744036 1.069014678 0.965322548 0.60814687 2.631104282 0.455228571

TCGA-CM-6163 1.169863014 0 0.385486259 0.66120233 0.046002047 4.58369959 2.126576457 1.676086834 0.618990353 0 2.372144185 0.18599337 4.254624275 2.543693838 2.447817202 1.428624817 1.578552333 1.243730004 0.753946772 0.267595722 0.214870834 0.134352387 4.499144296 0.193582525 0.486662893 1.324465006 0.595408178 3.050432042 0.445091015

TCGA-AA-A004 1.161643836 0 0.055056793 0.387913924 0.023042393 3.810371122 0.317651385 0.264716569 0.996605677 1.540870414 0.264836648 0 0.841651143 1.364068101 0 0.52717045 0.219091058 4.940697677 0.925999419 0 0 3.044429093 6.65156695 0 2.013962426 0.637934776 0.325386415 1.600602791 0.533663013

TCGA-AA-A03F 0.002739726 0 0.03562391 0.179383654 0.141955886 4.026410568 0.786345437 0.539828811 0 0.386700602 1.144568518 0 1.938398719 0.490980804 0.629006833 1.094776557 0 2.007231389 1.110229592 1.847916755 0 0.658005777 4.538513332 0 2.612729933 0.83802395 0.217727042 2.784231751 1.956502778

TCGA-A6-2686 3.084931507 1 0.103933991 1.067914014 0.02446153 3.153643226 1.414352056 1.5091891 0.993058391 0.899407681 1.265256848 0.492622329 4.150397202 2.078029088 1.665392771 1.823464053 1.964398632 0.530670075 0.954717448 0 1.680414382 0.599603448 5.469114293 0.590242708 0.76366652 0.881194737 1.181357028 2.927877496 0.966651949

TCGA-F4-6855 3.950684932 0 0.575989948 0.595408178 0.143262793 4.920841115 2.316087802 1.469990138 0.916476644 0.241840184 2.567813129 0.502636117 2.72617728 2.066433674 0.980537192 1.595885528 2.378345149 0.845429833 1.177088602 0 0.935157976 0.074231394 4.004932788 0.346871814 0.342554745 1.604166235 0.521251795 3.100035721 1.763284085

TCGA-AA-3666 0.167123288 1 0.181547856 0.082838815 0.065158675 3.398035636 1.642701572 1.849438881 0.65250944 0.866314561 2.136355469 0.108758614 2.150104701 1.994507316 0.982217846 0.815821322 1.026162654 1.630638446 0.983166916 0.660015865 0.936741574 0.308127844 4.819652857 0.184216808 0.488515009 0.655443425 0.522256668 2.645217451 0

TCGA-A6-2677 2.02739726 1 0.634035597 1.236053748 0.590817563 5.419298422 1.971013127 1.923364729 1.556110553 0.381209528 3.224014541 1.461161815 5.311067102 2.443244164 3.649193893 3.314894671 2.847141723 1.54550716 1.911244165 2.794519841 2.569564003 0.053157687 4.785808589 1.782296724 0.322658875 1.903501093 2.334596878 3.679596449 1.139622292

TCGA-A6-2675 3.619178082 0 0.161694235 0.556012427 0.089091319 3.699485004 2.126609494 1.052485453 0.382169209 0 2.781548563 0.414027259 3.498289169 1.713255902 2.037276784 1.309583671 0.485323774 1.45817199 0.959621798 0.38703161 0.978122395 0.39583057 2.815780343 0.282973489 0.099227866 1.277211136 0.470510835 3.15367565 1.322216605

TCGA-CK-5913 4.276712329 0 0.140255134 0.198116929 0.564719594 3.893954313 1.688941238 0.802978987 0 0.079975377 2.460270761 0.475396222 1.135403531 1.323197108 1.596982834 0.39583057 0.055334502 1.165815488 0.389676937 0 0.464250034 0.092072095 2.792709618 0.226138561 0.290542363 1.306028869 0.312084262 3.130799111 0.146394549

TCGA-G4-6311 3.284931507 0 0.259302621 0.689120211 0.187387707 5.239329297 1.960029743 1.286230606 0.917546326 0.099093179 2.700328737 0.217106608 2.718262977 1.73109633 2.01171031 1.364964568 1.46346552 0.551392968 0.897859974 0 0 0.341644241 3.839778232 0.358733827 0.231432408 0.908736064 0.610983471 3.311226989 0.736561583

TCGA-AA-3525 0.002739726 0 0.148088083 0.362554049 0.255077682 3.204954508 0.971663103 0.724824869 0 0.288181352 1.802482761 0.527971113 1.602884409 1.587076903 1.907390643 0 0.684818738 0.785843455 0.593879595 0.952259007 1.073820233 0.173639164 4.965128575 0.286289758 1.267296078 0.867026632 0.495797478 3.169941031 1.025808419

TCGA-D5-6536 1.487671233 0 0.372060582 0.526769951 0.461109415 3.570754355 2.005364055 1.537793034 0.503959271 0.833659246 1.508682179 0.426103701 3.427632988 1.326364767 1.745667602 1.415488271 1.557189496 1.505535292 1.005902957 1.012854681 1.139534981 0.252657864 3.850109308 0.309176187 0.641268561 0.835843249 0.563939074 2.55269676 1.234930448

TCGA-AA-A01G 1 0 0.086104371 0.814755483 0.105946212 4.578624787 0.946581156 0.92782062 0 0.462052319 2.016246697 0.28166858 2.663390165 1.365524577 2.046944996 1.628820246 0.602836912 2.356622751 1.001153695 0 0.505281211 0.149519521 5.050109844 0.700262145 0.746570751 0.665665638 0.737167557 3.132362972 0

TCGA-NH-A8F7 1.487671233 0 0.271067049 0.584144742 0.101179409 6.318548233 1.817132044 1.60345425 0.603691617 0.586548593 2.211775405 0.138093596 3.235995052 1.433386809 2.925049965 2.102254994 2.077328464 1.564085453 1.391905165 0.160145725 0.613955913 0.463727072 4.642603387 0.388299768 0.495388174 0.647130275 0.755143958 3.503526835 0.9721412

TCGA-QG-A5YX 2.747945205 0 0.432531879 0.312665174 0.024603367 5.36273638 1.9202933 1.069495954 0.438186376 0.205642742 2.286999397 0.336283388 4.393388741 1.07819992 2.767993718 1.272321853 1.634732653 0.53216732 0.733961663 0.282143229 0.749148067 0.180147861 3.680076759 0.266516713 0.137503524 1.236523232 1.218037159 3.340975222 2.089396454

TCGA-AA-3864 4.416438356 0 0.2174789 0.620492574 0.024035935 4.037461296 1.60445093 0.609944032 0 0 1.874993639 0.099227866 2.348629021 1.286703756 0.916247324 0.546956178 0.425244442 2.688896492 0.792105706 0.611266825 1.413052429 0.10057404 5.568819533 0.168513676 0.513591806 0.738465217 0 2.933459333 0.553655402

TCGA-AA-3852 0.002739726 1 0.333194716 0.453965265 0.08106688 4.07153959 1.213627239 0.445832624 0.595694607 0.575796375 1.800868846 0.189160371 3.095856924 1.670477804 1.206705731 1.370666485 1.331361267 2.074847916 1.019559595 0.602741913 0 0.615416297 5.452826026 0.572501665 1.214622201 0.728573603 0.98170655 2.135370694 1.10400111

TCGA-DM-A28H 9.756164384 0 1.077858235 0.13553487 0.022048165 5.742405107 1.189666447 1.703898038 0.869081754 0.543495883 2.561863763 0.242328116 4.245069667 1.435361897 2.999134123 1.557826675 2.643556078 2.676786432 0.740452698 0.660381035 0.937118365 0.145220937 5.214389068 0.540324906 0.682393511 0.838830781 0.661658403 3.18321653 0.425566724

TCGA-AA-3844 1.243835616 0 0.24610401 0.392757034 0.029982866 4.630160808 2.074231394 1.289421365 0 0.500802053 1.49067281 0.236094579 3.298145071 1.22292818 1.982473426 1.636357806 0.584000383 2.40729836 1.03083043 0.908966582 1.913147921 0.275126274 4.891462934 0.270349529 0.93696766 0.44625623 1.492981163 3.265211832 0.256406854

TCGA-AA-3877 2.583561644 0 0.224040274 0.799170217 0.478298822 3.945111364 1.939489888 0.9395275 0.967168608 0.156396617 1.975556821 0.543891766 0 2.752748591 1.394569017 1.041313082 1.967685081 1.907275263 1.257795134 0.977316563 1.540225701 0.300885149 4.491436258 0.295723025 1.230141516 0.737254104 0.789437136 3.694557082 0.894449162

TCGA-CM-6676 0.923287671 0 0.292428393 0.550802184 0.095721922 7.622087703 1.766849545 1.287708686 0.807190033 0.163369914 2.451646328 0.095586908 4.423988198 1.16690822 3.252021989 1.219834524 1.238236574 1.433867485 0.966651949 0.816067173 0.999206299 0.187387707 4.285831264 0.308127844 0.692784257 0.640250721 0.868765772 3.769179309 0.292546188

TCGA-AA-3548 2.832876712 0 0.300650908 0.646439186 0.021053252 3.788163657 1.568567238 1.137044411 0.382833229 0 1.949236091 0.169796756 2.037136187 1.916935177 0 0.489645688 0.745108226 2.195882997 0.58870864 0 0.44826666 0.088548697 4.567600205 0.283566239 1.012425648 0.819014122 1.03083043 2.954363839 0

TCGA-A6-2683 1.380821918 1 0.078883047 0.532067552 0 2.152735075 0.60928218 0.82887526 1.083315502 0.655443425 0.906043966 0.091801369 1.859611811 1.19496955 0.8623522 1.542307584 0.635150724 2.760540327 0.863224853 0 0 1.961438118 5.424304042 0.156008221 1.198682729 0.51470301 0.68670221 1.884949517 0.719752957

TCGA-AA-3811 0.838356164 1 0.545968369 1.015354834 0.053806444 2.091665988 0.899330335 1.280184295 0.702746186 0.537643962 1.372116318 0.40315863 2.35634104 1.10701825 0.850319378 1.416191194 1.629566451 1.984880959 1.274291484 0.967168608 1.324810603 0.886823248 4.91551612 0.642008357 0.955833538 0.559149148 0.163112244 3.158870183 0.509239782

TCGA-CK-4951 5.846575342 1 0.293605911 0.32849183 1.145286163 4.393072922 1.851918907 1.060047384 0 0.136060107 1.951363988 0.358958826 1.49758682 1.671519837 1.464145457 0.242206149 0.493442391 0.898479256 0.837862529 0 0.730661691 0.297367523 3.524301932 0.372617844 0.368041931 1.090311471 0.794187097 3.389195077 1.200629898

TCGA-A6-2684 3.087671233 0 0.955412005 0.966430467 1.600602791 4.268898449 1.582716561 3.072420266 1.630063707 0.209598928 2.38294387 0.550539535 3.47132188 2.103844494 1.668678904 2.268993237 2.400556145 1.301646173 2.152215875 2.322822289 2.936220184 0.552081911 3.531988564 1.456595905 0.911908848 1.876867244 1.953674984 3.812107977 2.688396728

TCGA-AA-3715 1.58630137 1 1.52992087 0.936289295 1.826762016 1.546215385 0.679063417 0.567253375 0 1.785383152 1.779469857 1.204391163 1.216113358 2.133760781 1.111164889 1.770321635 0 1.048724443 0.808755717 0.757023247 0 1.432318067 6.280334648 0 0.781485616 0.751892138 0.230080016 2.269063078 3.006244157

TCGA-AZ-4315 4.865753425 0 0.163885117 0.074505436 0.565792121 4.285002648 1.658599958 0.651407656 0 0.896078055 2.17960659 0.189666447 0.183962835 0.950617715 0.523461591 0 0.117030053 2.250991882 0.497944422 0.604261139 0.49559284 0.191941593 5.439164327 0 0.507515586 0.263755573 0.334225008 2.514273783 1.386369519

TCGA-A6-2671 3.646575342 1 0.215616477 0.227001675 0.056444801 4.406951569 0.982874961 1.000793264 0.631988957 0.260387037 2.031677496 0.293841299 1.969601794 0.910962861 1.791397348 0.936289295 0.860207991 3.133168933 1.165236648 0 0 0.754032318 4.237464293 0.135140816 0.704340813 0.271784214 0.274530045 2.381366443 0.800413313

TCGA-AA-A01V 0.084931507 0 0.13395801 0.330099418 0.028710586 5.341427199 0.876173114 1.202010167 1.252355101 0.48326116 1.175939705 0 2.462104684 1.146264195 2.390695197 1.504722077 1.065779085 1.562962829 1.058870606 0 0.413052429 0.264716569 5.058905231 0.135928816 0.98112199 1.078678143 0.276079728 2.500317675 0.64099104

TCGA-AA-3502 2.917808219 0 0.204265945 0.804549245 0.24902051 4.471675214 1.687150294 0.419431022 1.078404892 0 2.744634739 0 3.737319011 1.574343754 1.276317993 0.577827591 2.163627539 0.661931977 0.690908713 0 1.473579128 0.157949154 5.168998975 0.338909273 1.657228401 1.082702589 1.31254901 3.753529661 1.321754961

TCGA-AA-A01P 3.17260274 1 0.29372361 0.261711329 0.814181243 1.160533009 1.126972856 0.474565835 0.715190517 0.244521774 1.117229582 0.276198865 1.961178784 1.701815171 1.210077099 0.59636272 1.010851435 2.079395179 0.762391341 0 2.132642643 0.371614618 4.134253803 0.450802153 0.625364016 1.287590496 0.948526095 3.410422519 4.511891778

TCGA-AZ-4616 0.42739726 1 0.309176187 0.300650908 0.459536538 4.490339075 1.057068969 1.428088778 0.183835831 0.424599662 1.197299271 0.116098552 3.445514838 1.164207025 0.605494334 0.928427177 0.770025564 3.008110354 0.879392103 0.869476634 1.481298942 0.136191386 5.224572849 0.313478058 0.39088776 0.49559284 0.626766188 3.013730228 1.005902957

TCGA-AD-6963 2.284931507 0 0.10648233 0.174917695 0.064882852 5.796060381 1.409852525 0.783582374 0.725610294 0.788100998 1.899523692 0.034779158 1.555178088 1.070045788 2.660152815 1.533961965 1.081407806 2.298922199 0.894992331 0.445302942 0.513389678 0.199625236 4.226161686 0.277746757 0.416731671 0.40990682 0.294664856 3.519365771 0.401193551

TCGA-CM-6169 1.084931507 0 0.344374033 0.872946928 0.052555009 5.971398702 2.121744855 1.708319689 0 0 2.872454497 0.109293689 3.688482521 1.966799585 1.978634962 1.184915004 0 0.928351371 0.944108799 0 0.545375359 0.056444801 4.243402511 0.752320428 0.344828497 1.231862449 0.36982938 3.335926241 0.601125981

TCGA-CK-4948 12.33424658 0 0.160016608 0.125915549 0.110764122 5.943466604 1.853836004 0.71633248 0.234501321 0.420832692 1.748074745 0.394404385 2.961697406 1.318345767 2.004069868 0.833740194 0.812539298 2.203451766 0.860684757 0 0.770194755 0.182310918 4.955136083 0.27524549 0.296310561 0.427820684 0.344033092 3.187815245 1.07819992

TCGA-DM-A282 11.59726027 0 0.155360663 0.45143533 0.037171338 5.296339931 1.02446153 1.397967206 0 0.158724796 1.91494715 0.092748686 3.467592509 1.525617899 1.25011268 1.002018361 0.074916402 1.788685711 1.039559557 0.863779904 1.275722256 0.078746447 3.715816513 0.387362541 0.845831239 0.564231818 0.51752767 3.696795088 0

TCGA-AA-3861 2.504109589 0 0.085560625 0.569782712 0.149649582 5.056765588 1.262493294 0.27596058 0.604830437 0.459116814 1.8483976 0.09936254 3.782010091 1.186944201 2.03885755 0.547548539 1.653243496 1.241107975 0.556699167 0.611927767 0 0.051163253 4.81991338 0.168642036 0.939151337 0.368489001 0.261350279 2.883992194 0.55424502

TCGA-CA-5796 1.032876712 0 0.200253232 0.866235421 0.016068369 3.318649454 2.804322077 0.097745622 0.299596349 0 2.094877876 0.130799111 3.842968778 1.815288501 2.183931085 0.386148755 1.439197579 0.240497517 0.273456211 0 0.352532557 0 4.361992886 0.114367025 0.266996372 0.587845009 0.73135705 3.422784672 1.439782689

TCGA-CK-4950 7.120547945 0 0.156396617 0.282380495 0.003314389 4.344871096 1.487949337 0.565499693 0.188400925 0.138028045 1.766001425 0.297837036 1.517426885 1.174917695 1.99088227 0.638212885 0.655077003 0.449217992 0.356481902 0.35952117 0.416299305 0.133300476 2.851719063 0.092883966 0.29795439 0.522557993 0.399663304 2.64878754 0.393635856

TCGA-AA-A029 4.331506849 0 0.135009441 0.428464026 0.266156864 6.141101672 1.183645305 0.326422296 0.354903461 0.679874148 2.017530012 0 0 0.732573121 0.776946192 0 0.869555597 0.524163999 0.667574266 0 0 0.301236441 6.185022084 0 0.576473765 0.189286907 0 2.614898265 0

TCGA-AA-3549 1.750684932 0 0.382169209 0.236462004 0.097071371 4.50719207 1.898092236 1.50156653 0.193708673 0 2.138683428 0.161049225 1.357608304 1.219896462 1.68063942 0.466757616 0.889317778 0.746054735 0.96547032 0 1.44498504 0.504467853 5.431763345 0.269392279 1.179765808 0.635893663 0.680504401 2.748890543 0

TCGA-G4-6309 7.123287671 0 0.309409049 0.13066734 0.236094579 3.560397037 1.510506259 1.498812516 0.385044426 0.19761381 1.672923127 0.170822399 0.79110557 0.958657108 2.752769996 1.535057595 1.076559095 1.69737351 1.074847916 0.389897162 1.152248331 0.117562069 4.241359714 0.52326084 0.480471945 0.660928617 0.390777727 3.087055435 0.63254742

TCGA-A6-6142 2.090410959 0 0.556797247 0.335826224 0.12035194 3.842717414 2.098891126 0.71501475 0.397364885 0.752234781 1.886081854 0.176705738 3.811152452 1.538836108 2.679964201 1.086851688 1.205455074 1.581447665 0.532067552 0 0.325846898 0.150559677 3.252627591 0.612210935 0.334110568 0.64791311 0.529371205 3.751195896 1.714223534

TCGA-F4-6570 0.515068493 1 0.807931885 0.287472295 1.087666501 4.414588842 1.36485254 0.916476644 0 0.295723025 1.324292177 0.333194716 2.69884077 1.965728884 1.146199013 1.242389096 0 0.696795088 1.000360629 0.972325042 1.095586908 0.199122642 4.521333467 0.358733827 0.415866811 1.52656966 0.882760416 3.449772646 1.873970646

TCGA-AU-3779 1.208219178 0 0.3154503 0.231309516 0.065710164 5.132013308 1.579469817 1.049770152 0.5663768 0.42867841 2.085866507 0.299244658 2.369298962 1.408222729 3.295326302 0.713079899 1.36999684 0.324004081 1.173831016 0 0 0.243059706 4.320946729 0.65315176 0.154323965 0.607768235 0.660563586 3.261982057 0.721328908

TCGA-G4-6626 0.002739726 1 0.303108551 0.107420059 0.017351842 6.396823951 2.096666669 1.399936681 1.219834524 0 2.48339016 0.660289751 3.009383006 1.365524577 2.60916396 1.409635325 2.409282306 2.808240877 0.888616627 0.814263291 0.48377709 0.272023189 4.072380247 0.374955996 0.610511092 1.241596155 0.789687524 2.855072777 1.006262113

TCGA-A6-2678 3.523287671 0 0.466444406 0.194213156 0.042644337 4.440141617 2.348374075 0.847756437 0.207892852 0 2.982582947 0 3.582291043 1.283329168 2.191910018 0.867263911 0.619459965 0.572404647 0.916553077 0 0.80182086 0 4.568524678 0.5290713 0.076695902 0.788602195 0.869871406 4.233098103 0.273933569

TCGA-AA-3713 1.58630137 0 0.868370697 0.158466295 0.120484657 2.459116814 1.232783534 0.807519792 0.458906907 0.517225293 1.990010902 0.577730931 2.557704163 2.14133469 3.207970917 2.065710164 1.026233491 1.404630684 1.207518077 0 0.781065898 0.442014566 4.807483728 0.459011864 0.73092249 1.012139555 1.060324133 2.751977807 1.007482576

TCGA-AA-A01X 2.167123288 0 0.244278199 0.36120689 0.088820033 5.086032167 1.657685732 0.745452483 0.727354154 0.886823248 1.772223506 0.147697444 1.215181565 0.697862762 3.067053536 1.150689643 1.071694033 1.613578799 1.047538389 0 0.394404385 0.726831217 4.545084942 0 0.547844628 0.943358763 1.070389328 2.541068729 0.614097305

TCGA-D5-6898 0.62739726 0 0.356369214 0.223051788 0.0528332 6.676332296 1.783917573 1.101784524 0.885730534 0.473215434 2.320398027 0.13093087 2.121976877 1.527670916 2.468687573 1.343066652 2.070458026 1.144633773 0.999206299 0.554932602 0.453754607 0.273217473 4.120716881 0.350836665 0.492929907 0.986738576 0.435841916 3.22983399 0.391657754

TCGA-D5-6533 2.123287671 0 0.030265443 0.153027043 0.025028794 5.393244572 1.703942322 0.752406071 0.624147697 0.540424105 2.112633417 0.152118504 2.722597173 1.205204813 1.72617728 1.863502405 1.588324868 2.138224691 1.099093179 1.546956178 0.721591399 0.399663304 4.10187695 0.255077682 0.212756083 1.041663532 0.389236386 2.155425432 0.449006639

TCGA-AZ-4614 0.471232877 1 0.495183478 1.254231207 1.153351383 5.454856932 1.808590988 1.384547202 0 0.138159145 1.147957881 0 2.164400135 2.486946009 0.440845918 1.417487999 0.924175915 3.223252629 1.007482576 0 2.005112498 2.008379317 5.097876221 0.37784561 1.12498977 0.970706433 0.512075149 3.156364254 1.213440607

TCGA-DM-A28G 5.065753425 1 0.175684271 0.738292263 0.036327492 4.337047385 1.00252251 1.303342394 0.50650039 0.249991368 1.708143082 0.090582476 4.048820331 0.924556002 1.458801942 1.261771495 1.761710784 1.215181565 0.86710573 0.847996907 1.176769556 0.10648233 4.266434255 0.498965654 0.859969548 1.165107985 0.679423798 3.001135675 0.437866901

TCGA-AA-3514 0.084931507 0 0.056860943 0.340277405 0.035483152 4.710001881 0.368600747 0.333881659 0.599127326 0 0.761200156 0 2.096767855 0.687687811 1.21238257 0 0.747258486 1.776735687 0.499578047 0 0 1.947553953 5.216695424 0 0.8366513 0.193834811 0.335483256 2.305299344 1.389787054

TCGA-AA-3977 2.084931507 0 0.110496882 0.144307467 0.410015403 4.082370486 1.369215194 0.867026632 0 0 2.337340025 0.763156584 2.971092857 1.737556978 0.901648911 1.234746552 0.878686111 2.53565486 0.38603836 2.847195187 1.782869818 0.098419558 3.770490792 0.313245852 0.877273089 0.798589739 0.332049084 3.85941303 0

TCGA-G4-6297 6.865753425 0 0.71606903 0.921588666 0.653335227 4.401917121 1.939076093 1.490262049 0.253747285 0.096531743 3.083383588 0.260146127 2.255530946 2.012211084 2.504467853 1.978232246 0.651132078 1.492058265 0.94665601 0.370275897 0.547054922 0.263154625 3.40083846 0.185739709 0.420940456 1.47134356 0.79360461 2.874442887 1.199813663

TCGA-AA-3980 0.663013699 0 0.157173094 0.33433944 0.267475872 3.769041746 1.717999879 1.015354834 0.189286907 0 2.053563194 0 3.031889185 1.898285759 2.501795794 1.420994335 0.69937395 1.950057757 1.28504705 0 1.593258146 0.268194823 4.902657294 0.379177329 0.671565126 0.384602458 0.715190517 3.161371766 1.434241233

TCGA-AA-3556 1.917808219 0 0.800993057 0.347665656 0.048375705 4.963275277 1.994000358 1.047817548 0 0.171847314 2.712375673 0.283092058 4.90078954 2.471733734 2.572647178 0.553360503 0.120219212 0.351967481 0.509949146 0 1.179447354 0.242450074 4.053928053 0 0.168513676 1.236155823 0.740366343 3.82758533 0.560127976

TCGA-DM-A1HB 11.30410959 0 0.139993302 0.216113358 0.460375619 4.602064868 1.370108469 0.525668007 0 0.115565996 1.931683057 0.757364672 0.043624477 2.047363887 1.128161401 0.94425876 0.486353976 0.789186704 0.703632307 0.435735259 1.624615633 0.034356597 3.761636329 0.456806149 0.45143533 0.644502372 0.20739313 3.017690346 0.303809967

TCGA-AA-3531 2.835616438 0 0.325040955 0.263515224 0.044464067 5.386186701 0.919301941 1.628960189 0.281549893 0.948825083 2.640574657 0.235236889 0.979513226 0.385927957 2.575868968 2.053563194 0.803805652 2.4333601 0.883151571 0 1.023964991 1.527921084 5.656442025 0.547844628 1.535057595 0.624615633 0.655535016 3.26034187 1.60164898

TCGA-AA-3845 0.002739726 1 1.151858817 1.453807274 1.006692983 1.256346463 0.983677695 1.75364732 0.263515224 0 2.825012184 0 1.806736492 2.29234004 0.597125899 0.616169466 0.759922794 0.704606413 1.846914479 3.653851103 1.957208861 0.36960607 3.966873397 0.653243496 0.709643545 0.770110162 1.248291938 4.47118746 4.429334762

TCGA-AA-3660 6.506849315 0 0.148869044 0.27369491 0.023326332 5.210700563 1.796680808 1.665847521 0.945982186 0.165043649 2.28433645 0.501209824 0.237318966 1.162854528 2.57456174 0.922882871 1.718131434 1.296193073 0.79601624 0.597125899 0.85431488 0.452279135 5.231378644 0.164013889 0.53116933 0.939377047 0.658280045 3.148999163 0

TCGA-AZ-4313 6.328767123 0 0.287235865 0.473215434 0.098689044 2.394925655 1.01306915 0 0 0.808179084 1.35822745 0 1.219648693 0.136191386 0 0 0.600174586 2.787244384 0.526469505 0 0.585443319 0.430392334 6.159506077 0 0.527270557 0.188907267 0.2131295 2.947535252 0

TCGA-G4-6588 2.180821918 0 0.518333702 0.554637964 0.710525442 3.165284893 1.58731698 0.827575168 0.558463573 0.42234066 2.095249317 0.662387819 1.156267163 1.310456462 1.293488202 1.528621325 1.697329024 0.919225654 1.153935011 0.655626601 1.492314685 0.186120184 4.072731805 0.623211368 0.047259175 1.378678078 0.481815574 3.017048902 2.270768126

TCGA-AA-3875 1.504109589 0 0.155878733 0.380175312 0.104873377 5.765773408 1.595408178 1.407787806 0.474150461 0.50589093 1.983422328 0.310107409 3.012640181 1.461476173 1.580676144 0.828550347 1.639602631 2.125122061 1.195410597 1.473059537 0.552377071 0.684728987 4.782665417 0.502839758 1.2864672 0.574440641 0.455228571 3.224040274 0

TCGA-AA-3662 0.504109589 0 0.547449829 2.132017423 0.161823203 4.235650482 1.655122811 2.203389117 0.455439015 0.458382004 1.94721729 0.204891925 3.156704022 1.76366652 1.713563855 1.834508974 1.281787257 1.035553533 1.54823932 0.746656736 0.617392528 0.579662897 5.071333641 0.277627748 0.477573719 1.041102772 0.647360565 3.088514776 0.486147994

TCGA-AA-A01C 1.252054795 0 0.291957116 0.374510926 0.245130531 5.287065907 2.09713881 1.403049529 0.399116393 0.755400369 2.206049271 0.177598929 2.876074862 0.931607422 2.091598292 0.885574364 1.072380247 2.702391584 1.040401515 0 1.7193587 0.179765808 3.926682639 0.295958068 1.795143529 0.401084302 1.297837036 3.668754533 0.516015147

TCGA-A6-6652 2.057534247 0 0.3305584 0.353097411 0.055750964 5.255500733 2.480084127 1.844666853 1.816026201 0.082021269 2.327054969 0.047398789 3.457935687 1.720716243 2.532541389 1.88869455 2.702147745 1.266336799 1.16998912 0.319039816 0.475084883 0.266756562 5.315544494 0.367930141 0.193204014 1.46383168 0.63988042 3.243501529 0.150169706

TCGA-AA-3831 1.498630137 0 0.139076515 0.341871921 0.008630305 5.390461336 1.323485365 1.059770581 0.45385994 0 2.333967504 0.13920752 2.69859633 1.265016749 2.436721535 0.727092709 0.981560432 1.349874774 0.531768207 1.087462841 1.471395589 0.299596349 4.383365608 0.435308552 0.998917573 0.540424105 1.40310408 3.247000854 0.221815228

TCGA-A6-5659 2.536986301 0 0.349704962 0.860446393 0.11196609 4.418111221 1.714677803 1.56488215 0.904015166 0.238175419 2.43805327 0.313903671 3.456753591 2.450415074 3.070933099 2.142587705 1.35663214 1.185951096 1.41705586 1.422430371 1.005208334 0.01259251 2.855644259 0.912036637 0.080202841 0.762759842 0.92425194 3.74682869 0.191183609

TCGA-CK-5912 4.090410959 1 0.663116867 0.26447638 0.022474347 5.622655135 2.446388581 1.40135741 1.130799111 0.390997784 3.059528335 0.062812492 4.478402378 2.141792439 3.593425486 1.368433124 2.591272495 2.080350674 0.476329838 0 0.332736572 0.124460486 1.947254701 0.47134356 0.29713271 1.355692898 1.428249611 3.710977206 0.369382725

TCGA-D5-6537 0.4 1 0.056444801 0.109828565 0.075327251 5.853501694 1.620914791 0.948526095 0.674189443 0.243303487 2.223515225 0.315913965 2.902922541 1.27100727 1.763539053 0.929866728 1.948899821 1.768459602 0.669026766 0.829443681 0.267955212 0.050188223 3.798081629 0.38294387 0.124195772 0.455123338 0.603406772 3.68513282 1.023823091

TCGA-DM-A0XF 3.183561644 1 0.277032552 0.413918977 0.050327554 5.530002053 2.154485998 1.771632081 1.516922851 0.951363988 2.233029057 0.216982489 4.083579315 1.25821739 2.248899107 1.523662314 2.589787452 3.222263604 1.153416242 0.894138688 1.662023157 0.190172345 3.570851483 0.569199413 0.482848283 0.741057041 0.516317779 3.550420507 0.465295389

TCGA-CM-6171 1.169863014 0 0.715190517 0.701593413 1.042714369 2.659056854 1.025454096 1.187514398 0.668391477 0.084880654 2.018384921 0.230203013 2.813524689 1.096599207 1.657228401 1.360083296 1.771378539 1.131260215 1.132708439 0.822607794 0.489337409 0.166972472 4.27799962 0.679423798 0.374733478 0.840120773 0.47019844 3.325818122 0.749662978

TCGA-AA-3870 2.498630137 0 0.36960607 0.132905812 1.106415327 3.976317806 1.660700483 0.603596675 0.208642109 0.153156788 2.331418598 0.173639164 1.576038337 2.659056854 1.760859637 0.271306144 0.299596349 2.497075806 0.728312379 0.559344967 0.804301423 0.473734968 4.450676804 0.152118504 0.076969476 0.392866916 0.083111227 2.52789607 1.176067406

TCGA-CM-6161 1.252054795 0 0.19887128 0.874678949 0.011638756 7.361404307 1.42932137 1.309991039 0.741833681 0.084744621 2.76964481 0.186627327 4.253981705 1.45132982 2.150689643 0.640250721 1.887642241 0.841731643 1.164271398 0 0.488515009 0.049630768 5.509094066 0.443924858 0.122738972 1.199059805 1.230203013 3.360139496 0.42234066

TCGA-D5-6539 1.04109589 0 0.123798608 0.275483893 0.058108649 4.348607777 1.64316353 1.144307467 0.593688409 0.166201253 2.858677301 0.097206246 1.586548593 1.327572404 3.209422172 1.366979584 1.591344313 0.847916755 0.735435526 0 0.687419077 0.212756083 3.53792967 0.313245852 0.343692069 0.955015156 0.662114331 2.906602028 0.652876515

TCGA-CM-4743 1.920547945 0 0.151858817 0.197488002 0.846713941 2.056167306 1.484653748 1.119820952 0.211386062 0.226878404 2.153772916 0.256406854 1.264236151 2.40206725 1.00662118 0.608241513 1.009992048 1.746269764 1.098486934 1.137700241 2.009526333 0.113833829 3.885925722 0.225398337 0.44498504 1.310805431 0.624334889 3.447341152 2.479204688

TCGA-AA-3554 1.495890411 0 0.212507085 0.274768566 0.372952098 2.588828548 1.425727838 0.9053509 0 0 2.200692666 0.187894405 0.182183769 1.166265537 2.291633025 1.232415171 0.739070393 2.983969487 0.977389839 0 0.491288732 0.397145797 4.331891488 0.312432837 0.619647767 0.699462794 0.255319441 2.566595994 0.542505701

TCGA-AA-3872 0.002739726 0 0.350836665 0.265556916 0.08841301 3.510253053 1.744591687 1.048724443 0.935761456 0.357833479 1.766764756 0.075327251 2.789917009 1.417271945 1.415974946 1.146459722 1.552918042 2.026304324 0.595503661 0 0.700794799 0.747258486 4.493006791 0.246590503 0.451646328 1.388244653 0.376734905 3.102842865 0.768671318

TCGA-AA-3678 3.917808219 0 0.118093889 0.104470858 0.058108649 5.577304947 2.070148859 0.70061727 0.4473147 0.335254565 2.501107892 0.320773477 2.171046661 1.250840334 2.035694283 1.194528369 1.320426911 2.08280476 1.402394751 0.452806263 1.455859809 0.636729013 4.552444704 1.027791019 0.986811375 0.722378586 0.501719377 3.623094284 0.408059648

TCGA-AA-3672 0.002739726 0 0.280481274 0.191309967 0.235481995 1.998267726 0.705579862 0.353662045 0.543594864 0 0.7212414 0.645332754 0.57104572 1.269811154 0 0.685536541 0.538438835 3.155182533 1.250476553 0 0 1.389071144 6.450718038 0 0.976216976 0.468322644 0.431890348 1.927403465 0.693587315

TCGA-AA-3812 2.920547945 0 0.243547227 0.49067281 0.053806444 3.531917887 0.321235435 0.39758394 0.335025838 0 0.843019046 0.808920427 1.413594082 1.123136427 0 0.430285273 0.601030869 1.158337027 0.479127064 0 0 1.916247324 5.485745602 0 0.634593268 0.178109076 0.262192588 1.37928825 1.041102772

TCGA-AA-3680 0.917808219 1 0.135666197 0.833092483 0.428571222 5.502400621 2.430017587 1.014569539 1.035975744 0 2.49487638 0.428035163 4.670557116 2.229126433 1.053180862 0.355354621 2.394569017 0 1.097543379 0 1.005471851 0.33616911 4.552579959 0.378733559 0.799253124 1.483312761 1.288181352 4.22497408 1.577247536

TCGA-NH-A6GC 1.065753425 0 1.154064674 0.597507338 0.028569152 3.866294777 1.925087955 1.825175049 0.696706079 0 3.367622677 0.42234066 2.831938028 1.389566812 2.654045942 1.853317042 1.700572884 1.096194373 1.552573811 0.704606413 1.57758593 0.255561159 4.369229156 0.373731722 1.783289011 1.740279983 2.160887927 3.280377339 2.590841511

TCGA-A6-4105 1.210958904 1 0.202386375 0.483364361 0.116897018 5.280143456 1.02219024 1.56007905 0.350723534 0.178873958 1.665756583 0.461423785 1.706110557 1.637239266 2.567618457 1.675770776 1.182374488 1.448530982 1.093222111 0.355129058 1.464406886 0.228726355 4.701022227 0.783163266 0.255682003 0.859810565 0.355918372 3.029488224 0.168256923

TCGA-AA-3867 2.002739726 0 0.177854025 0.366364183 0.051163253 5.721728094 1.684818738 0.698307394 1.852358467 0.488103633 2.060704577 0.10648233 2.265856921 1.162790092 2.117761525 0.581013735 0.797012978 1.463674765 0.743730376 0 0.533264313 0.25592366 3.670795024 0 0.838669451 0.672561119 0.649845352 2.97379493 1.004609253

TCGA-5M-AAT6 0.794520548 1 0.311968051 0.224040274 1.155749233 2.774291583 1.331590576 1.190741268 0.148478616 0.303225478 2.002126407 0.236462004 0.983604737 1.86710573 0.873261995 1.724693923 0.893051503 2.394102512 1.245982361 0.992260861 2.130271955 0.063640993 5.140684599 0.472071799 0.879627357 1.417379976 0.456175325 2.454623375 1.739416093

TCGA-AA-3955 1.747945205 0 0.152897287 0.529271244 0 5.748445131 1.808096689 0.956279732 0.562279064 0.228356954 2.689813518 0.258096758 2.45638563 0.971663103 0.686791838 0 1.528471302 1.086308223 0.78098194 0 1.440580184 0.200127654 3.908226874 0 1.421748432 0.74760223 0.240985904 3.286984616 0

TCGA-AA-3860 2.589041096 0 0.253263199 0.244156396 0.070938821 5.280147168 1.880332889 0.893595198 1.225768496 0.146655222 1.579083579 0 2.679423798 1.331991778 0.816558749 0.840765337 1.923795728 1.116763971 0.393745671 0.930018176 1.279055216 0.610038557 5.235022387 0.14574266 0.557483614 0.763921421 0.539828811 2.413918977 1.140124224

TCGA-G4-6293 11.09863014 0 0.066399229 0.201006466 0.01678155 5.854457016 1.858179974 0.982509934 0.487383443 0 2.564500116 0.260868739 2.882858215 1.22342255 2.465974465 1.048724443 2.181643261 0.463517834 0.767824268 0 0.973648008 0.32308179 4.104277944 0.487589248 0.422125333 0.968348847 0.633942631 3.130519083 0.624989873

TCGA-AD-A5EK 1.369863014 0 0.532067552 0.842375488 0.023894043 6.711877556 2.206236862 2.224194664 0 0.320542442 2.591176732 0.040401515 0.026162654 2.146785541 2.657045428 1.595646873 0.184851546 0.815985227 1.346077535 0.506703487 1.111432005 0.247441471 6.064469017 0.758388465 0.369941022 1.222062625 1.338738165 3.306918391 0.457436698

TCGA-CM-6166 1.832876712 0 0.145220937 0.275007047 0.084200362 6.429610943 1.127765328 1.421694581 0.537643962 0.112633417 2.586428496 0.188021051 5.160702413 2.155457815 2.062639828 0.926454935 1.119024103 2.127699305 1.218533207 0 1.058593577 0 3.192241518 0.488823464 0.308710351 0.402394751 0.992913418 3.768078435 0

TCGA-D5-6540 1.345205479 0 0.619459965 0.353887836 0.677440588 0.843260306 1.876566059 1.222557291 0.23793077 0.09017595 1.85379609 0.288181352 1.672832634 2.13874895 2.358902579 1.903771004 0.656542133 1.427981546 1.448795255 0.628353671 1.324868195 0.376957114 4.272165062 0.534559685 0.559638645 1.689030727 0.969012308 3.48080797 1.919263798

TCGA-F4-6809 1.104109589 1 0.271545198 0.555128994 0.615039565 5.219714511 1.675454648 2.022722894 0.589475878 0.347552277 2.754417214 0.390777727 2.593855699 1.528921324 1.904580435 0.878686111 1.473786912 1.527670916 1.418621747 0.65617599 1.413594082 0.177981556 4.799828155 0.892507603 1.315624192 1.316667104 1.007482576 3.478467097 1.387858796

TCGA-NH-A50T 1.515068493 0 0.130535557 0.170181458 0.004753055 6.027501322 1.597698019 0.985791854 0.09396575 0.312781328 2.728399459 0.077653186 2.087089389 1.187261005 2.541291802 1.8812339 0.584000383 1.113033665 1.262733813 0.69099808 0.403485883 0.097610797 5.706129902 0.783414745 0.250597823 0.306728867 0.329525485 2.547128975 1.124460486

TCGA-D5-5540 4.673972603 0 0.33330923 0.343464676 0.039840265 3.564743979 1.299303279 0.742092468 1.171591153 0.272620455 2.957858895 0.109962253 3.16997309 1.557287542 2.072071491 1.482022175 2.177822141 1.641268561 1.276853945 1.46430232 1.107821756 0.111298453 5.183192705 0.350949786 0.226138561 0.601030869 1.071968558 2.905735978 0.475084883

TCGA-CK-5915 0.002739726 0 0.34879896 0.886511129 0.007051943 7.087262335 1.188843984 0.516922851 1.598460492 0.196481149 2.856368255 0.058939855 0 1.705535629 2.629799561 1.146264195 2.295752407 0.399116393 0.915635623 0 0 0.030124161 4.483235359 0.195221593 0.280243696 0.658005777 0.826517981 2.969878033 0.6296597

TCGA-D5-6926 0.753424658 0 0.419970287 0.830255324 0.118359726 5.188614559 1.654847942 1.691042761 0.245982361 0.679874148 2.665688375 0.506094112 3.451224302 1.729269968 2.583687557 1.942007714 1.07231164 1.382169209 1.552524629 0.359408719 0.290306435 0.301236441 4.465961408 0.483880254 0.668573016 1.39835037 0.915482658 3.142625824 0.586500555

TCGA-AA-3685 3.087671233 0 0.303809967 0.270827916 0.054779032 5.672948578 1.772307975 0.902575299 0.552180304 0.468322644 2.002882509 0.113833829 0.256286071 0.890252115 1.685222546 0.339935493 2.113067014 0.274172189 0.913416033 0.685446835 0.213751646 0.077926579 4.809882575 0.192572941 0.531967777 0.576570509 1.011638756 2.374010058 0

TCGA-QG-A5Z2 2.608219178 0 0.421263701 0.741833681 0.402176425 4.360139496 2.392757034 1.70230292 1.126378217 0.412618959 2.848297437 0.313942357 3.614226902 2.042154018 2.949871056 1.890952471 1.59187087 1.094844104 1.585395244 0.263034406 1.879431314 0.564426949 4.970048354 0.600840628 0.430071128 2.041838725 1.203764966 3.806045598 1.10614728

TCGA-AA-3663 0.580821918 0 0.09328973 0.082566351 0.16889872 3.857025515 1.27482819 0.602836912 0.361656083 0.863859179 2.438771896 0.303459302 2.421559945 1.816804475 1.147111287 0.913492628 1.023326332 2.566352443 0.802482761 1.727528424 1.49646235 0.055889758 4.688678333 0.346417994 0.618426617 0.449957484 0.28368476 2.943039879 0.822607794

TCGA-AA-A01Z 3.084931507 0 0.318577154 0.434668256 0.044603951 5.139829633 1.662342242 1.56924803 0 0.159112461 2.086953565 0 0.717999879 0.914028675 2.110095929 0.719314887 0.56627937 2.268853547 0.983239895 0.578407414 0 0.224904644 5.145172016 0.300533772 0.752834209 0.600364915 0.318692833 2.239886801 0

TCGA-T9-A92H 0.991780822 0 0.253384236 0.261350279 0.004896843 5.90857516 1.514551532 1.329123596 0.439357178 0.644779219 2.484963029 0.159629186 3.563292385 1.548633903 2.565134074 1.867896464 1.369382725 3.142364421 1.08120326 0.519340608 0.890252115 0.122871469 3.879637158 0.439463568 0.984188293 0.818605184 1.270050456 2.894099874 1.168962884

TCGA-AA-A03J 3.41369863 0 0.181802255 0.390337512 0.058801354 4.477606098 0.911193023 1.119887336 0.193077822 0.599413018 1.375400929 0.234501321 1.417433988 1.338281778 1.069702167 1.334968651 0.277746757 1.087327052 1.251507027 0 0 1.151728955 5.185636647 0.268554164 2.08004362 0.565499693 0.218843151 2.879686164 0

TCGA-A6-5662 1.967123288 0 1.281787257 0.21971064 0.07231164 5.514624117 2.193330195 1.244339096 1.272142662 0.088955682 4.082268285 0.240863823 3.64893706 2.130436712 3.193330195 1.197550907 1.504061001 1.256829515 1.047119549 1.230080016 0.886276995 0.07751647 5.446434901 0.590338533 0.69884077 0.962586038 0.622649279 3.146997284 0.162596766

TCGA-CA-6718 0.838356164 1 0.535356259 1.05963216 0.888382834 1.532117437 0.76366652 1.026091814 0.414893223 0 2.325875674 0.630032634 3.665415512 2.455623127 1.830782647 1.149259365 1.133563526 3.344899494 1.600983311 0.325961996 0.262673687 0.209141398 5.11788617 0.440527032 0.160145725 1.58990727 1.034356597 2.609589506 3.867481382

TCGA-AD-6889 6.936986301 1 0.349704962 0.510961919 0.357608304 2.452094595 1.012783185 1.413973119 0.11196609 0.23155529 1.245982361 0.136716384 3.344516069 1.678342385 1.709334754 1.550999139 1.186437169 0 0.685087956 0.576183494 2.681561708 0.264836648 5.008029655 0.365356597 0.32849183 0.345282818 0.635707964 3.051285085 0.724999445

TCGA-G4-6294 2.350684932 1 0.40403114 0.709467101 0.038997979 5.535533561 2.246924872 1.529770982 0.190678066 0.732052073 2.770469649 0.120484657 3.753230123 1.799584701 2.416650612 1.783498562 1.174725988 0.736561583 1.388850793 0.516116031 0.592923408 0.216361735 3.863125715 0.589667624 0.29172142 1.416839742 1.110162762 3.50984783 0.562279064

TCGA-CM-6674 1.079452055 0 0.578310793 0.277627748 0.366140336 2.916705929 1.918004519 0.53126916 0.371168515 0.189919418 2.152183419 0.056860943 1.596124144 1.50217782 1.460165895 0.722466024 0.826517981 1.507769274 0.695102986 0.375845725 0.954494126 0.085560625 3.736063628 0.274530045 0.389676937 0.57695742 0.246712101 3.424384672 0.730835562

TCGA-AA-3984 0.002739726 0 0.36120689 0.809085118 0.59139219 4.935615324 1.182946495 1.073546061 0 0.149389449 2.478971805 0 1.62849366 0.898014819 1.113700499 1.143785225 0.292663973 2.939489888 1.068326861 0.943358763 1.669843154 0.395720913 4.288771967 0.51944126 0.513389678 0.439144376 0.30018231 3.296736375 0

TCGA-AA-3552 1.084931507 1 0.127765328 0.526769951 0.043484498 4.501572899 1.892818428 0.970043751 0.211635253 0.295487943 1.967353084 0.09071796 2.752898418 1.136978812 1.937043014 1.422233001 1.312432837 2.239642442 1.099429872 0.56627937 1.86844972 0.70017335 4.049700461 0.293605911 1.639093213 1.430017587 1.140647793 3.541787394 2.273724745

TCGA-AZ-4323 0.117808219 1 0.629566451 0.622180704 1.021550794 3.01678155 1.040191072 1.744979109 0.707171362 0.423739507 1.585539463 0.98170655 2.820035963 2.905062024 1.596028702 1.681449265 1.975079854 2.452173687 1.597316631 0 1.436375082 0.729705026 4.652624161 0.480782124 0.385596696 1.862867922 0.5383395 3.026074104 0.890096434

TCGA-F4-6805 2.868493151 0 0.29419431 0.508022917 0.178746506 4.658200055 1.623773238 1.14110576 0.155101558 0 2.392509769 0.065710164 3.16145239 1.514803986 1.96476827 0.681224354 0.356707253 0.776693582 0.460690149 0.427927928 0.626859618 0.249748715 4.466222508 0.21685836 0.163369914 0.800578978 0.282617721 2.82452348 1.153610802

TCGA-DM-A28E 9.994520548 0 0.317651385 0.480575346 0.01564029 5.520890498 1.725523046 1.34953513 0.201633861 0.81204635 2.097880434 0 1.874442887 1.03562391 2.672289553 1.482951513 0.830823203 1.199436784 1.165815488 0.54260475 1.175811994 0.228603232 4.759672512 0.619178216 0.244887059 0.930018176 0.626205483 3.863859179 0
