# Supplementary material for: Disulfidptosis-related long non-coding RNA signature predicts the prognosis, tumor microenvironment, immunotherapy, and antitumor drug options in colon adenocarcinoma
Source: Apoptosis. 2024 Aug 8;29(11-12):2074–90. doi: 10.1007/s10495-024-02011-x (PMC11550253; doi:10.1007/s10495-024-02011-x)
Supplement: Supplementary file 5 — Supplementary Material 5 [file 10495_2024_2011_MOESM5_ESM.docx]

| gene | lowMean | highMean | logFC | pValue | fdr |
| --- | --- | --- | --- | --- | --- |
| GRIN1 | 0.881649312 | 1.71622971 | 0.960965841 | 1.18E-06 | 0.000136406 |
| AC010973.2 | 0.867436086 | 1.246738986 | 0.523330091 | 6.74E-06 | 0.00044044 |
| SEMA6D | 3.313785703 | 2.303112754 | -0.524895266 | 8.64E-06 | 0.000520152 |
| PCP2 | 0.854450994 | 1.412100145 | 0.724772752 | 8.71E-07 | 0.000115844 |
| GOLGA8B | 3.041757492 | 4.457145797 | 0.551215016 | 9.56E-05 | 0.002415276 |
| DUX4L50 | 1.534866055 | 2.446301087 | 0.67248922 | 0.000410993 | 0.006243095 |
| SIX4 | 0.669073165 | 1.316788043 | 0.976787254 | 0.000457592 | 0.00667109 |
| TERT | 0.84634396 | 1.464320145 | 0.790914996 | 4.14E-06 | 0.000322212 |
| KRT20 | 580.6650878 | 404.4814983 | -0.521632583 | 6.36E-05 | 0.001903563 |
| NPC1L1 | 3.173124006 | 4.959163841 | 0.644192987 | 0.005771845 | 0.03401124 |
| MC1R | 1.248493807 | 1.847124493 | 0.565092439 | 1.06E-07 | 3.31E-05 |
| EREG | 43.57860925 | 27.92004594 | -0.64231884 | 7.88E-05 | 0.002168663 |
| SLC16A9 | 10.00608479 | 6.860168188 | -0.54456173 | 6.66E-05 | 0.001969424 |
| CDH13 | 3.118075765 | 2.153170072 | -0.534193706 | 0.008729323 | 0.044645063 |
| MAPK12 | 1.585091437 | 2.691903551 | 0.764060654 | 0.000104433 | 0.002551906 |
| UCN | 2.8657987 | 4.125874855 | 0.525762787 | 7.20E-10 | 1.28E-06 |
| CBY2 | 1.040391743 | 1.678326667 | 0.689896692 | 4.81E-06 | 0.000355497 |
| DRD4 | 1.577309404 | 2.610675362 | 0.726957384 | 2.74E-08 | 1.29E-05 |
| RPL13P12 | 185.9781477 | 288.0621572 | 0.63124703 | 0.000218333 | 0.004150315 |
| TRIM40 | 3.016200382 | 2.058905145 | -0.550854912 | 0.000788086 | 0.00955693 |
| PKLR | 4.297409251 | 2.908635362 | -0.563124728 | 0.00813143 | 0.042461558 |
| DOC2A | 1.087140596 | 1.605424783 | 0.562416542 | 0.00256755 | 0.020194125 |
| SUZ12P1 | 5.539725229 | 8.527969348 | 0.622387832 | 0.009456381 | 0.046967476 |
| MUC12 | 37.9680604 | 25.54777891 | -0.57158843 | 9.44E-05 | 0.002403419 |
| WNT10A | 2.75779656 | 5.078472101 | 0.880878482 | 0.00697489 | 0.038554187 |
| MLXP1 | 1.029345107 | 2.287313333 | 1.151927257 | 0.001049591 | 0.01134628 |
| CDKN2A | 4.353934098 | 8.939028333 | 1.037798449 | 0.001465858 | 0.014032551 |
| MIR4728 | 1.729924847 | 5.328237464 | 1.622949016 | 0.007788554 | 0.041215135 |
| LINC02253 | 3.703197095 | 5.393911667 | 0.54256056 | 0.004688352 | 0.029610966 |
| HOXC4 | 0.905770719 | 1.621515507 | 0.840125015 | 0.0006272 | 0.008195523 |
| FSCN2 | 0.847469648 | 1.237998333 | 0.546775767 | 6.25E-05 | 0.001901642 |
| AC068299.1 | 1.046852523 | 1.646088116 | 0.652983351 | 8.28E-05 | 0.002229385 |
| AFAP1-AS1 | 2.621125917 | 4.557921667 | 0.798189469 | 0.001890495 | 0.016441576 |
| AMH | 3.456917813 | 6.287791232 | 0.863067013 | 5.33E-06 | 0.000367972 |
| FKBP2 | 0.967214602 | 1.390468116 | 0.523662733 | 5.57E-05 | 0.001775235 |
| ADRB1 | 0.846414373 | 1.593028116 | 0.912335698 | 0.000312808 | 0.005279102 |
| AC135050.3 | 0.914914985 | 1.405609855 | 0.619486614 | 1.97E-05 | 0.000886625 |
| HBQ1 | 0.826903517 | 1.977182101 | 1.257654841 | 0.003260907 | 0.023486564 |
| SLC26A3 | 212.4350846 | 101.2135541 | -1.06961955 | 5.14E-06 | 0.000361626 |
| SLAIN1 | 1.719182416 | 2.694562609 | 0.648328477 | 0.002150924 | 0.017980053 |
| KRT16 | 2.342612615 | 10.46588935 | 2.159504603 | 0.009018254 | 0.045685864 |
| AMIGO2 | 15.99713456 | 23.01214949 | 0.524582238 | 0.00114819 | 0.011995756 |
| MMP17 | 2.45909159 | 3.558748623 | 0.53324456 | 0.000478743 | 0.006903801 |
| ANO1 | 14.95486911 | 23.80412384 | 0.670596245 | 5.99E-05 | 0.00184899 |
| LCN12 | 4.119359633 | 6.082036232 | 0.562134327 | 0.000205816 | 0.004006488 |
| TNIP3 | 1.488075994 | 0.929736449 | -0.678554484 | 0.001148188 | 0.011995756 |
| ZDHHC11 | 0.900721177 | 1.504258768 | 0.739900279 | 4.27E-05 | 0.001505061 |
| AREG | 239.7980139 | 167.6123196 | -0.516691518 | 4.79E-05 | 0.001605396 |
| HHLA2 | 33.71699625 | 23.79191297 | -0.503004741 | 3.61E-05 | 0.001333847 |
| CASC9 | 6.171456804 | 9.731592319 | 0.657064798 | 3.60E-09 | 4.32E-06 |
| TRIM7 | 2.63539419 | 3.793550942 | 0.525530142 | 0.000949394 | 0.010730723 |
| ZEB1-AS1 | 1.78949289 | 2.605243478 | 0.541867397 | 5.09E-10 | 1.28E-06 |
| NXPE4 | 49.48515344 | 32.97766551 | -0.585506478 | 0.009346456 | 0.046563676 |
| TMEM252 | 9.135241514 | 5.612600725 | -0.70277344 | 0.004612166 | 0.029325921 |
| SLC25A21-AS1 | 0.85838448 | 1.383969565 | 0.68911632 | 7.88E-06 | 0.000485702 |
| RPL37AP8 | 9.966177676 | 14.23500326 | 0.514330624 | 0.009911034 | 0.04846903 |
| SLC29A4 | 2.415807492 | 3.956047971 | 0.711554425 | 0.001130622 | 0.011888589 |
| BMP3 | 1.325189985 | 0.765093333 | -0.792491548 | 0.00606333 | 0.035162029 |
| AC105219.1 | 1.376215749 | 2.342318551 | 0.767230634 | 1.90E-05 | 0.000875291 |
| PLCH2 | 1.230376911 | 2.598267536 | 1.078449653 | 1.35E-05 | 0.000687721 |
| ACSL6 | 3.791262232 | 2.62228413 | -0.531854234 | 6.29E-05 | 0.001903563 |
| C16orf74 | 1.532570031 | 2.443976739 | 0.673277555 | 0.00824609 | 0.042983624 |
| IL20RB | 1.284623242 | 2.624636159 | 1.03077214 | 0.003320295 | 0.023753257 |
| NOXA1 | 34.05282102 | 48.49941848 | 0.510193129 | 1.01E-07 | 3.20E-05 |
| ARHGAP44 | 6.458984786 | 4.240631159 | -0.607028416 | 7.45E-09 | 6.81E-06 |
| AC009097.2 | 1.006047401 | 1.473539928 | 0.550587872 | 0.00110029 | 0.011704173 |
| FAM160A1-DT | 3.659278593 | 2.476330725 | -0.563355252 | 7.69E-10 | 1.28E-06 |
| IL13RA2 | 3.552037615 | 1.886392391 | -0.913017055 | 8.86E-05 | 0.00232005 |
| AC095057.3 | 0.868987538 | 1.292429928 | 0.57267867 | 0.000327343 | 0.005413917 |
| AC087379.1 | 1.69989526 | 1.006648696 | -0.755885563 | 0.010288274 | 0.049484049 |
| KIF26B | 3.016191208 | 4.40782442 | 0.547338868 | 0.004413632 | 0.028610422 |
| RAB26 | 4.89666422 | 9.218518116 | 0.912735586 | 0.001250999 | 0.012724724 |
| SYCE2 | 1.195592431 | 1.822335942 | 0.608063271 | 4.36E-07 | 7.68E-05 |
| HOXC11 | 1.453607722 | 2.194153116 | 0.594026217 | 0.001054315 | 0.011380532 |
| TFAP2A-AS1 | 1.062600153 | 1.532074638 | 0.527887757 | 0.002636359 | 0.020547614 |
| ATP2A1 | 0.999848394 | 1.502568043 | 0.587649062 | 1.69E-06 | 0.000172805 |
| SLC4A11 | 5.527488456 | 9.070258478 | 0.714519557 | 0.000293612 | 0.005061948 |
| RN7SL521P | 1.180177523 | 1.890560652 | 0.679810247 | 0.001974674 | 0.016952084 |
| TMEM121 | 1.510485168 | 2.39074058 | 0.662445573 | 0.001145244 | 0.011995756 |
| PAH | 2.284574083 | 1.486281159 | -0.62021817 | 0.00118514 | 0.012285336 |
| HUNK | 17.5616815 | 12.2521813 | -0.519392366 | 1.43E-05 | 0.000721421 |
| HSF4 | 8.316987768 | 12.80168297 | 0.622200471 | 1.28E-08 | 8.93E-06 |
| CPNE7 | 28.83844373 | 41.57735203 | 0.527804567 | 0.002785749 | 0.021270215 |
| LINC00858 | 1.241318119 | 1.825176884 | 0.556163397 | 0.000394245 | 0.006106415 |
| WDR72 | 5.89963945 | 8.542152899 | 0.533972933 | 5.96E-05 | 0.00184899 |
| LINC02474 | 1.772624083 | 2.650891667 | 0.580591094 | 0.00543502 | 0.032563399 |
| STK31 | 1.477784786 | 2.533092101 | 0.777463352 | 0.002521577 | 0.019939697 |
| AC245100.6 | 1.473033486 | 2.112197609 | 0.519954586 | 0.006187671 | 0.035475493 |
| ONECUT2 | 0.926338456 | 1.505520942 | 0.700651465 | 0.0004116 | 0.006243095 |
| LGALS9C | 2.993308486 | 1.869503623 | -0.679085701 | 0.000730805 | 0.009111429 |
| CTXN1 | 8.800770107 | 13.2413421 | 0.58934768 | 0.001744388 | 0.015731739 |
| GNG8 | 0.863362768 | 1.598288261 | 0.888488847 | 0.004510972 | 0.02898468 |
| NAMPTP1 | 7.236191131 | 4.639563696 | -0.641241374 | 2.17E-06 | 0.000208073 |
| CERNA2 | 4.121919343 | 2.318967899 | -0.829833426 | 1.56E-05 | 0.000767595 |
| PAQR6 | 3.156143272 | 4.918149928 | 0.639953018 | 3.76E-08 | 1.57E-05 |
| FAM95C | 1.034122706 | 1.534862029 | 0.569701593 | 0.000207848 | 0.00402949 |
| ERFL | 0.813539144 | 1.321668406 | 0.700076595 | 0.000132956 | 0.003007854 |
| ARHGEF4 | 0.996344878 | 1.639415507 | 0.718464437 | 0.001071073 | 0.011510496 |
| AC245100.4 | 0.846818807 | 1.348429058 | 0.671154406 | 0.007129358 | 0.039083408 |
| SNORD94 | 9.149431881 | 13.42264058 | 0.552914445 | 0.005801385 | 0.034143493 |
| AP006621.2 | 3.503718502 | 5.375526884 | 0.617519298 | 3.67E-06 | 0.000298321 |
| EFNA5 | 2.243790214 | 3.200459565 | 0.512341286 | 0.00266501 | 0.020639038 |
| QRICH2 | 1.300010627 | 1.85183442 | 0.510431691 | 6.95E-07 | 0.000105956 |
| TBC1D3L | 1.406706422 | 2.038486377 | 0.535177044 | 1.17E-05 | 0.000628121 |
| ADAMTS13 | 1.947376453 | 2.853749493 | 0.551326896 | 1.68E-07 | 4.40E-05 |
| EBF4 | 3.793063532 | 5.55939413 | 0.551564129 | 1.69E-05 | 0.000809462 |
| AP000553.6 | 1.931398624 | 2.928628116 | 0.600579053 | 0.000174089 | 0.003558856 |
| HMGA1P4 | 2.746969954 | 4.076838188 | 0.569609566 | 6.50E-06 | 0.000432527 |
| SP5 | 18.76594732 | 29.13259254 | 0.634516973 | 0.00027202 | 0.004837855 |
| AC012354.2 | 2.31813578 | 4.230431449 | 0.867839735 | 0.003043949 | 0.022471524 |
| GPR15 | 3.079532187 | 1.877661232 | -0.713774412 | 0.006730246 | 0.037642573 |
| CRYBA2 | 1.00163555 | 1.738211087 | 0.79524562 | 0.00029805 | 0.005114363 |
| TLE6 | 1.10842974 | 1.66008913 | 0.582743377 | 9.97E-05 | 0.00247244 |
| APOD | 20.04761743 | 34.70525884 | 0.7917235 | 0.003096903 | 0.022755706 |
| TAS2R38 | 0.82888318 | 1.211198696 | 0.547194863 | 0.001488671 | 0.014185988 |
| FZD2 | 4.147193043 | 5.876513261 | 0.502825205 | 3.17E-06 | 0.000276271 |
| EIF4HP2 | 1.682682951 | 2.393199493 | 0.508177291 | 1.37E-06 | 0.000150291 |
| LGALS9B | 4.376656498 | 2.767010072 | -0.661501263 | 0.000783791 | 0.009512726 |
| SYT8 | 1.195876223 | 2.016296377 | 0.753639643 | 9.10E-06 | 0.000524509 |
| BTNL3 | 20.06005313 | 14.12995514 | -0.505568541 | 0.002897627 | 0.021828395 |
| AL136295.6 | 2.24708341 | 3.274004348 | 0.543002561 | 3.84E-09 | 4.32E-06 |
| AF127577.3 | 1.182563303 | 2.066314203 | 0.805142233 | 0.002295118 | 0.018704697 |
| LINC02846 | 1.188552064 | 1.867693768 | 0.652052825 | 0.000276289 | 0.004890015 |
| AC006435.2 | 1.012770719 | 1.456020725 | 0.523723292 | 5.93E-06 | 0.00040232 |
| YJEFN3 | 1.819189373 | 2.973718188 | 0.708972202 | 8.64E-10 | 1.28E-06 |
| PILRB | 2.41843448 | 3.452568188 | 0.513596455 | 0.000285434 | 0.004985558 |
| RGS11 | 1.256127905 | 2.046590797 | 0.704239299 | 0.004293662 | 0.028144348 |
| PCDHGB2 | 0.832214985 | 1.371172971 | 0.720382405 | 0.007711228 | 0.040998906 |
| RUBCNL | 25.58517469 | 16.95767558 | -0.593369653 | 2.81E-06 | 0.000250786 |
| MIRLET7BHG | 1.125026529 | 1.657993768 | 0.559479562 | 0.00147512 | 0.014102131 |
| AC078860.3 | 1.039025612 | 1.511034565 | 0.540305446 | 0.007936089 | 0.041754326 |
| AC127024.4 | 2.534197171 | 5.04623529 | 0.9936787 | 7.68E-05 | 0.002148684 |
| HABP2 | 1.241774618 | 2.255865725 | 0.861277849 | 0.003171727 | 0.023073839 |
| PRR36 | 4.92969419 | 7.132019928 | 0.532812581 | 6.88E-06 | 0.000441729 |
| MMP3 | 110.9585276 | 75.99021623 | -0.54613496 | 0.001084983 | 0.011608837 |
| AHCYL2 | 46.09731361 | 32.43405029 | -0.507173482 | 1.04E-06 | 0.00012697 |
| CLCA4 | 39.27295336 | 14.88206051 | -1.399961804 | 0.006336898 | 0.036090731 |
| AC010653.3 | 1.823477905 | 2.594452971 | 0.508737666 | 0.000708899 | 0.008921885 |
| POU6F2-AS2 | 0.806288226 | 1.553311812 | 0.945979905 | 0.005703657 | 0.033744714 |
| AC011498.6 | 1.051747018 | 1.533788043 | 0.5443114 | 8.22E-05 | 0.002229068 |
| CEACAM1 | 144.5493648 | 94.29216478 | -0.616352469 | 7.28E-08 | 2.54E-05 |
| AC110285.2 | 3.040454664 | 4.735671449 | 0.639281916 | 2.35E-05 | 0.001000236 |
| UPK3B | 1.216518272 | 2.03403471 | 0.741586309 | 0.002836082 | 0.021508842 |
| SFRP2 | 78.75212538 | 146.5876041 | 0.896372345 | 0.009218059 | 0.046143886 |
| ATP2A1-AS1 | 1.866107722 | 2.92433558 | 0.648076607 | 1.91E-05 | 0.000878142 |
| NXF3 | 2.359776758 | 3.616101232 | 0.615784683 | 0.002629294 | 0.020536275 |
| AL360181.1 | 1.145490673 | 1.878931522 | 0.713946777 | 0.003370641 | 0.023967003 |
| NPW | 5.050643578 | 7.368478478 | 0.544899512 | 0.010055176 | 0.048750452 |
| AP000697.1 | 0.953962232 | 1.381645435 | 0.534383375 | 0.010067206 | 0.048792612 |
| SNORD104 | 57.32484534 | 86.10954145 | 0.587012548 | 3.27E-05 | 0.001250214 |
| AC156455.1 | 3.513774771 | 5.131188623 | 0.546271341 | 0.000129075 | 0.002947376 |
| AL353747.2 | 2.794575306 | 1.960197609 | -0.511629951 | 0.001815445 | 0.016124192 |
| SNHG25 | 54.73032286 | 113.0645279 | 1.046734107 | 0.004215342 | 0.027717859 |
| DIPK1B | 1.610150229 | 2.516754855 | 0.644369397 | 3.42E-07 | 6.96E-05 |
| TCAP | 1.029995336 | 4.372210652 | 2.085725106 | 0.003710081 | 0.025423433 |
| RNU7-140P | 1.533727676 | 2.22026529 | 0.533689723 | 0.009023478 | 0.045685864 |
| AP003555.2 | 0.634750765 | 1.46179529 | 1.203479157 | 0.000235283 | 0.004386882 |
| TMEM91 | 1.7768237 | 2.540804493 | 0.515984827 | 1.82E-11 | 1.33E-07 |
| FAM183A | 0.981776911 | 1.696242246 | 0.788875077 | 0.002047893 | 0.017386893 |
| FIRRE | 0.957569266 | 1.418697754 | 0.56711851 | 0.004552204 | 0.029121769 |
| AC006128.1 | 1.28540367 | 1.926633043 | 0.583860318 | 4.82E-08 | 1.77E-05 |
| MAB21L3 | 2.252906651 | 1.512035652 | -0.57529538 | 2.89E-08 | 1.32E-05 |
| UGT2A3 | 17.76874602 | 12.04806565 | -0.560540334 | 0.000265534 | 0.004763012 |
| AOAH | 30.30939014 | 21.29372058 | -0.509336775 | 0.000184111 | 0.003722139 |
| DAPK1 | 7.150976529 | 10.15279413 | 0.50566465 | 0.007983301 | 0.041927371 |
| PRRX2 | 4.845933028 | 6.978766087 | 0.52619751 | 0.009277131 | 0.046312879 |
| IGF2BP1 | 1.309032875 | 2.629982101 | 1.006551652 | 0.003892705 | 0.026208612 |
| AC138904.1 | 0.829358716 | 1.326002899 | 0.67701579 | 6.61E-05 | 0.001965625 |
| AC004884.1 | 2.603513761 | 3.991674203 | 0.616533941 | 0.009963849 | 0.048533481 |
| IGFL4 | 1.917443884 | 2.749424348 | 0.519945234 | 0.000988438 | 0.011002283 |
| CYSRT1 | 2.716451988 | 4.570336304 | 0.750576781 | 1.29E-06 | 0.000145298 |
| CILP2 | 0.989252752 | 2.16049587 | 1.126951394 | 0.003625444 | 0.025030952 |
| KREMEN2 | 2.208187309 | 3.936441594 | 0.834029519 | 6.36E-08 | 2.27E-05 |
| GAD1 | 1.065907645 | 1.894023261 | 0.829371607 | 9.06E-07 | 0.000118455 |
| EPHB6 | 1.274259098 | 9.893449783 | 2.956815014 | 0.001499457 | 0.014242411 |
| ADM5 | 1.76829052 | 2.584696812 | 0.54763974 | 1.72E-06 | 0.000174436 |
| HDAC10 | 1.132046254 | 1.65799558 | 0.550507255 | 5.00E-05 | 0.001633502 |
| AGAP13P | 0.900747401 | 1.346272609 | 0.579776085 | 0.000157846 | 0.003314769 |
| HSPB3 | 3.568946789 | 1.313386159 | -1.442207235 | 0.00488401 | 0.030480992 |
| BTNL9 | 4.587431957 | 6.622765072 | 0.529746923 | 0.0005404 | 0.007455083 |
| LYG1 | 2.065020948 | 2.963487899 | 0.52113975 | 0.000295273 | 0.005078632 |
| AC140076.1 | 2.04102867 | 3.37034529 | 0.723599955 | 0.002473637 | 0.019709647 |
| TRNP1 | 14.42340749 | 21.19248768 | 0.555140911 | 0.001724977 | 0.015633738 |
| AL590326.1 | 1.762515291 | 2.583086739 | 0.551460316 | 1.12E-06 | 0.000131295 |
| AL645608.7 | 1.110055122 | 1.794126449 | 0.692650256 | 8.58E-06 | 0.000518749 |
| ADRA2C | 6.149703135 | 9.036041739 | 0.555174166 | 0.001568798 | 0.014691297 |
| AC136475.3 | 18.54807576 | 30.69750051 | 0.726851666 | 0.001888333 | 0.01643254 |
| RPL34P31 | 6.182493502 | 8.777477609 | 0.505617593 | 0.00829339 | 0.043076776 |
| AC037487.3 | 1.850428058 | 2.617329203 | 0.500236348 | 0.001879041 | 0.016390659 |
| ERVH-1 | 1.267931575 | 0.820042464 | -0.628706368 | 0.00019646 | 0.003891186 |
| SLC51B | 19.3715344 | 13.34349428 | -0.537801717 | 6.36E-05 | 0.001903563 |
| KCNIP2 | 0.898210321 | 1.314883623 | 0.549809911 | 0.000253362 | 0.004612517 |
| AC016876.1 | 6.008082722 | 8.560555942 | 0.510799815 | 0.000153171 | 0.003253928 |
| AP001160.4 | 0.825012462 | 1.428964565 | 0.792482325 | 3.54E-07 | 7.10E-05 |
| RN7SKP80 | 2.705425765 | 4.449284493 | 0.717717694 | 5.78E-05 | 0.001818203 |
| KRT81 | 0.766019954 | 1.787906957 | 1.222817781 | 0.001579982 | 0.014783254 |
| SCT | 3.468669648 | 6.502393623 | 0.906588446 | 0.000895035 | 0.01036673 |
| SNHG7 | 21.0226013 | 30.39820913 | 0.532045135 | 4.92E-14 | 7.19E-10 |
| EGF | 2.094951988 | 1.164501884 | -0.847204207 | 0.000585676 | 0.00787194 |
| LINC01176 | 1.191944878 | 1.752343116 | 0.555967769 | 0.003471857 | 0.024384631 |
| FOXD1 | 1.884546483 | 2.822162319 | 0.582583588 | 7.79E-05 | 0.002160379 |
| DBH-AS1 | 0.810490902 | 1.242649348 | 0.616551356 | 0.003315376 | 0.023736978 |
| UNC5B-AS1 | 1.705567431 | 3.347457899 | 0.972814116 | 0.0096574 | 0.047555126 |
| AL583810.1 | 0.89144763 | 1.317784058 | 0.563892028 | 0.000945483 | 0.010697133 |
| SCX | 6.470586162 | 9.706337174 | 0.585030567 | 4.34E-06 | 0.000327373 |
| RNF207 | 3.488813609 | 5.037520725 | 0.529977346 | 1.45E-05 | 0.000726149 |
| LINC01748 | 0.961443654 | 1.522925435 | 0.663571091 | 0.00052369 | 0.007307196 |
| SNORD3A | 17.91007095 | 44.7709442 | 1.321791692 | 1.31E-06 | 0.000146055 |
| CEACAM6 | 1701.028603 | 1191.206664 | -0.51398367 | 3.15E-05 | 0.001218386 |
| ADSS1 | 1.181645948 | 1.673054783 | 0.501686855 | 0.000407077 | 0.006219631 |
| PGGHG | 41.46785543 | 63.86970109 | 0.623138261 | 1.22E-05 | 0.000641342 |
| C11orf86 | 9.695472706 | 6.084888913 | -0.672080314 | 0.005912497 | 0.034671965 |
| AC114730.3 | 1.030010627 | 1.478925725 | 0.521890376 | 1.29E-05 | 0.000671588 |
| GOLGA8A | 5.417081269 | 7.678743478 | 0.503354518 | 0.000290725 | 0.00504785 |
| FAM166C | 5.948047554 | 8.674145 | 0.544305378 | 5.06E-06 | 0.000361626 |
| ROBO3 | 1.290589985 | 1.932631957 | 0.582536187 | 0.0002559 | 0.004625093 |
| AC109446.2 | 1.46940474 | 2.540859783 | 0.790084929 | 0.001206802 | 0.012404463 |
| AC018638.7 | 1.384941131 | 2.155968768 | 0.638511625 | 0.000208167 | 0.004030354 |
| AL590560.3 | 0.841795948 | 1.250772319 | 0.571276726 | 0.001042397 | 0.011324945 |
| MIR181A2HG | 1.304597783 | 1.917830797 | 0.555870361 | 0.005795361 | 0.034121763 |
| SYCE1L | 3.566352217 | 5.451593043 | 0.612228678 | 4.46E-07 | 7.68E-05 |
| LRFN1 | 1.038870642 | 1.502087319 | 0.531952657 | 0.005981709 | 0.034882181 |
| NALT1 | 1.500826453 | 2.381399783 | 0.666052675 | 4.93E-09 | 5.16E-06 |
| COLCA1 | 2.708413532 | 3.94652471 | 0.54313475 | 0.001588573 | 0.014829076 |
| GAMT | 2.913466972 | 4.442626594 | 0.608675931 | 0.000355809 | 0.005716776 |
| GABBR1 | 5.18297737 | 7.919353188 | 0.611601509 | 7.43E-06 | 0.000467699 |
| CRABP2 | 21.71244419 | 32.73982094 | 0.592524291 | 0.010183211 | 0.04915952 |
| OTX1 | 1.884815443 | 2.69275942 | 0.514662075 | 5.85E-05 | 0.001833616 |
| TMEM236 | 6.618475765 | 4.047756304 | -0.709376567 | 5.13E-05 | 0.001666271 |
| OLFM4 | 3109.971615 | 1622.668941 | -0.938532724 | 0.009296899 | 0.046364128 |
| MIR4768 | 2.357160245 | 4.522649783 | 0.940118444 | 0.003754584 | 0.025620443 |
| ISYNA1 | 17.51988073 | 26.78596333 | 0.612484228 | 6.64E-05 | 0.001969424 |
| AC022144.1 | 1.320675994 | 1.957997174 | 0.568102114 | 0.001718472 | 0.015594099 |
| TUBB2B | 3.303040443 | 5.968057681 | 0.853466845 | 0.007636285 | 0.040748195 |
| XDH | 16.58602875 | 11.44337986 | -0.535455276 | 0.000128311 | 0.002939115 |
| DNASE1L2 | 1.368313456 | 2.200614203 | 0.68550748 | 6.58E-10 | 1.28E-06 |
| LINC02441 | 11.30106972 | 7.907286812 | -0.51520468 | 0.000141587 | 0.00313255 |
| PRKAG2-AS1 | 4.180785168 | 6.256626957 | 0.581611176 | 2.88E-05 | 0.001156632 |
| SHC2 | 8.484265979 | 12.65173862 | 0.576473901 | 0.000293612 | 0.005061948 |
| ABCA3 | 4.682994878 | 7.272631159 | 0.63504595 | 0.004868852 | 0.030429295 |
| AC072052.1 | 2.135465979 | 3.44737529 | 0.690947449 | 1.83E-08 | 1.03E-05 |
| CCDC78 | 2.31112393 | 3.76136913 | 0.702663272 | 1.38E-07 | 3.96E-05 |
| MAPK15 | 5.579864985 | 8.598762681 | 0.623898864 | 2.74E-07 | 6.09E-05 |
| CEACAM7 | 266.9591212 | 129.0776782 | -1.04837931 | 3.31E-06 | 0.000283717 |
| SYT17 | 1.128508716 | 1.713831667 | 0.602807854 | 9.87E-06 | 0.000551525 |
| AP003555.1 | 0.731385627 | 1.949255942 | 1.414219352 | 2.13E-10 | 7.78E-07 |
| AL162586.1 | 1.264238914 | 2.112692536 | 0.740813697 | 8.76E-10 | 1.28E-06 |
| ATP5MC1P2 | 1.850839602 | 2.738455435 | 0.565182529 | 0.000481995 | 0.00693022 |
| HES4 | 21.08199702 | 31.01968449 | 0.557172478 | 4.96E-06 | 0.000361426 |
| AL451050.2 | 1.534080963 | 2.257199493 | 0.557159305 | 3.84E-07 | 7.23E-05 |
| CASP5 | 15.28255757 | 9.698052464 | -0.656119038 | 1.92E-05 | 0.00087977 |
| DUOX2 | 72.77161422 | 45.62260804 | -0.673626891 | 0.000625986 | 0.008195491 |
| ASIC3 | 1.040164832 | 1.743556014 | 0.745220548 | 9.07E-06 | 0.000524509 |
| AL355987.4 | 2.533899006 | 3.760026594 | 0.569383842 | 0.000988452 | 0.011002283 |
| HOXC6 | 1.273183639 | 2.703469928 | 1.086371785 | 4.13E-08 | 1.63E-05 |
| AC007318.1 | 1.085527676 | 1.566046739 | 0.528730763 | 0.000192856 | 0.003835373 |
| HSPB1P2 | 3.070427599 | 4.654265217 | 0.600113838 | 0.006558178 | 0.036948563 |
